# Integrative taxonomy of Nearctic and Palaearctic Aleocharinae: new species, synonymies, and records (Coleoptera, Staphylinidae)

**DOI:** 10.3897/zookeys.1041.64460

**Published:** 2021-05-31

**Authors:** Adam J. Brunke, Mikko Pentinsaari, Jan Klimaszewski

**Affiliations:** 1 Agriculture and Agri-Food Canada, Canadian National Collection of Insects, Arachnids and Nematodes, 960 Carling Avenue, Ottawa, Ontario, K1A 0C6, Canada Canadian National Collection of Insects, Arachnids and Nematodes Ottawa Canada; 2 Centre for Biodiversity Genomics, 50 Stone Road East, University of Guelph, Guelph, Ontario, N1G 2W1, Canada University of Guelph Guelph Canada; 3 Natural Resources Canada, Canadian Forest Service, Laurentian Forestry Centre, 1055 du PEPS, PO Box 10380, Stn. Sainte-Foy, Québec, QC, G1V 4C7, Canada Natural Resources Canada, Canadian Forest Service, Laurentian Forestry Centre, Quebec Sainte-Foy, Québec Canada

**Keywords:** Canada, DNA barcodes, faunistics, morphology, North America, rove beetles, United States

## Abstract

A long tradition of separate Nearctic and Palaearctic taxonomic studies of the diverse aleocharine rove beetles (Coleoptera: Staphylinidae) has obscured the recognition of Holarctic species and detection of adventive species in both regions. Recently, integrated study of the two regions through detailed morphological comparisons and development of an authoritatively identified DNA barcode reference library has revealed the degree to which these two aleocharine faunas are interconnected, both naturally and through human activity. Here this approach is adopted to recognize new species, reveal Holarctic species, and recognize adventive species in both North America and Europe. The following new species are described: *Isoglossatriangularis* Klimaszewski, Brunke & Pentinsaari, **sp. nov.** from British Columbia; *Gnypetaimpressicollis* Klimaszewski, Brunke & Pentinsaari, **sp. nov.**, from Ontario, Maryland and North Carolina; *Aloconotapseudogregaria* Klimaszewski, Brunke & Pentinsaari, **sp. nov.**, from Ontario and Virginia; and *Philhygrapseudolaevicollis* Klimaszewski, Brunke & Pentinsaari, **sp. nov.** from eastern Canada. *Dasygnypetavelata* and *Philhygraangusticauda* are revealed to be Holarctic species, resulting in the following synonymies: *Dasygnypetavelata* (Erichson, 1839) = *Gnypetaminuta* Klimaszewski & Webster, 2008, **syn. nov.** and *Philhygraangusticauda* (Bernhauer, 1909) = Atheta (Philhygra) pinegensis Muona, 1983, **syn. nov.** The Nearctic species *Hylotaochracea* (and genus *Hylota*), *Thecturotatenuissima*, and *Trichiusarobustula* are newly reported from the Palaearctic region as adventive, resulting in the following synonymies: *Hylotaochracea* Casey, 1906 = Stichoglossa (Dexiogyia) forticornis Strand, 1939, **syn. nov.**; *Thecturotatenuissima* Casey, 1893 = *Athetamarchii* Dodero, 1922, **syn. nov.**; and *Trichiusarobustula* Casey, 1893 = *T.immigrata* Lohse, 1984, **syn. nov.** The Palaearctic species *Amarocharaforticornis*, *Anomognathuscuspidatus*, *Oligotapumilio*, and *Parocyusarubicunda* are newly confirmed from the Nearctic region as adventive, resulting in the following synonymies: *Parocyusarubicunda* (Erichson, 1837) = *Chiloporaamericana* Casey, 1906, **syn. nov.** and *Anomognathuscuspidatus* (Erichson, 1839) = *Thecturaamericana* Casey, 1893, **syn. nov.** The genus *Dasygnypeta*, **sensu nov.** is newly reported from North America, *Paradilacra* is newly reported from eastern North America, and *Haploglossa* is newly reported from Canada, resulting in the following synonymy: *Paradilacradensissima* (Bernhauer, 1909) = *Gnypetasaccharina* Klimaszewski & Webster, 2008, **syn. nov.** Native *Cypheawallisi* is newly reported from across Canada and *C.curtula* is removed from the Nearctic fauna. The status of both *Gyrophaenaaffinis* and *Homalotaplana* is uncertain but these species are no longer considered to be adventive in North America. Three new combinations are proposed: *Dasygnypetabaranowskii* (Klimaszewski, 2020) and *D.nigrella* (LeConte, 1863) (both from *Gnypeta*) and *Mocytascopula* (Casey, 1893) (from *Acrotona*). *Dolosota* Casey, 1910, **syn. nov.** (type species *Eurypronotascopula* Casey), currently a subgenus of *Acrotona*, is therefore synonymized with *Mocyta* Mulsant & Rey, 1874. Additionally, four new Canadian records and 18 new provincial and state records are reported.

## Introduction

Historically, taxonomic research on the hyperdiverse aleocharine rove beetle (Coleoptera: Staphylinidae) faunas of North America and better-known Europe has been conducted separately, with a few exceptions (e.g., Klimaszewski et al. 1979). More recently, a closer examination of Aleocharinae in these two regions has demonstrated that a number of species are shared between the Nearctic and Palaearctic, either naturally (Holarctic) or through human activity (adventive) (e.g., Muona 1984; Klimaszewski et al. 2007; Klimaszewski et al. in press). The interconnectedness of these assemblages, combined with the sheer diversity of the subfamily, have made it difficult to avoid describing synonyms of taxa from other regions, especially when those taxa have been described in entirely different genera (e.g., Gusarov 2003a). One strategy to broadly address this challenge is the publication of detailed illustrations of habitus and genitalia in comprehensive faunal treatments such as the recently available ‘Aleocharinae of Eastern Canada’ (Klimaszewski et al. 2018) and ‘the Danish Beetle Bank’ website (Hansen et al. 2017), the latter an online resource for the Danish beetle fauna. In the past few years, resources such as these have made it possible to efficiently cross-check Nearctic and Palaearctic aleocharines without consulting a comprehensive reference collection for each region.

In combination with careful morphological study, large-scale DNA barcoding (e.g., deWaard et al. 2019) has accelerated the discovery of Holarctic species, and the detection of new adventive species and potential synonyms in the Canadian beetle fauna (e.g., Pentinsaari et al. 2019) by algorithmically flagging potential taxonomic issues and novelties, and connecting authoritatively identified specimens to unidentifiable females, damaged specimens, or other life stages. This integrated taxonomic approach, as applied to Aleocharinae, has already resulted in the detection of adventive species of genera *Amischa*, *Atheta*, and *Myllaena* in North America (Pentinsaari et al. 2019), and has refined the classification of Holarctic species in *Atheta* (Klimaszewski et al. in press), *Boreophilia* (Klimaszewski et al. 2019), and *Gnathusa* (Klimaszewski et al. in press).

Here we broadly compare morphological and molecular data across the Nearctic and West Palaearctic Aleocharinae in order to better integrate the taxonomic knowledge of these two regions. We describe four new Nearctic species, propose revised generic concepts, report new distributional records, and propose a number of new synonyms that impact our understanding of Holarctic and adventive species.

## Materials and methods

Almost all specimens used in this study were dissected and their genitalia were subsequently examined on microslides. The genital structures were dehydrated in absolute ethanol, mounted in Canada balsam on celluloid microslides, and pinned with the specimens from which they originated. The photographs of the entire body and the genital structures were taken using an image processing system (Nikon SMZ 1500 stereoscopic microscope; Nikon Digital Camera DXM 1200F) and processed in Adobe Photoshop. Terminology mainly follows that used by Lohse et al. (1990) and Klimaszewski et al. (2018). The ventral part of the median lobe of the aedeagus is considered to be the part of the bulbus containing the foramen mediale, the entrance of the ductus ejaculatorius, and the adjacent venter (ventral part of the tubus of the median lobe) of the tubus; the opposite side is referred to as the dorsal part.

Depository abbreviations:

**CBG**Centre for Biodiversity Genomics, University of Guelph, Guelph, Ontario, Canada;

**CNC**Canadian National Collection of Insects, Arachnids and Nematodes, Ottawa, Ontario, Canada;

**cRW** Personal collection of Reginald P. Webster, Charters Settlement, New Brunswick, Canada (also known as RWC);

**LFC** Laurentian Forestry Centre, Québec, Quebec, Canada;

**MCZ** Museum of Comparative Zoology, Harvard University, Cambridge, Massachusetts, United States (C. Maier);

**NHMD** Natural History Museum of Denmark, Copenhagen University, Copenhagen, Denmark (A. Solodovnikov);

**NMNH**National Museum of Natural History, Washington D.C., United States (F. Shockley);

**UAM**University of Alaska Museum Insect Collection, Fairbanks, Alaska, United States (D. Sikes);

**ZFMK**Zoologisches Forschungsmuseum Alexander Koenig, Bonn, Germany;

**ZMHB**Museum für Naturkunde, Berlin, Germany (B. Jaeger);

**ZMUO**Zoology Museum, University of Oulu, Oulu, Finland (M. Mutanen);

**ZSM**Zoologische Staatssammlung, Munich, Germany (F. Koehler).

We have examined all DNA barcode data for Aleocharinae previously generated by a variety of projects in both Europe and North America (e.g., Rulik et al. 2017; Sikes et al. 2017; McClenaghan et al. 2019; and other studies summarized by Pentinsaari et al. 2019). Fifty-three barcode sequences, the majority of which are Canadian sequence records originating from various projects coordinated by CBG, are published here for the first time. All sequences were analyzed using the workbench tools of the BOLD platform (http://www.boldsystems.org) after applying filters to exclude those flagged as misidentifications, those with sequence lengths under 100 bp, those with stop codons, and those flagged as contaminated. Sequences were generally visualized as clusters in neighbour-joining trees (using the Taxon ID Tree tool). In addition, BIN Discordance Reports, which compare the taxonomy of the specimen records to their BIN assignments, were used to detect potential misidentifications and synonyms.

All COI barcode sequences in BOLD that fulfill quality criteria (minimum length 500 bp, less than 1% ambiguous bases) are automatically assigned into BIN clusters (Barcode Index Numbers; Ratnasingham and Hebert 2013). In addition, sequences between 300–500 bp can be assigned as members of an existing BIN, but they will not be accepted as founding members of a new BIN. BINs correspond to species at a high accuracy in northern and central European beetles (Hendrich et al. 2015; Pentinsaari et al. 2017), and we treat BINs here as provisional hypothetical species.

The DNA barcode sequences studied here, including both previously unpublished data and the sequences published in earlier studies, have been compiled into a publicly available dataset on BOLD (DS-ALEO2020, https://doi.org/10.5883/DS-ALEO2020) along with collecting data, images of the specimens (if available), and other metadata related to the specimens and sequences. The sequences are also available through GenBank (accessions provided in Suppl. material 1: Table S1).

## Taxonomic accounts

### Tribe Aleocharini Fleming, 1821

#### Amarochara forticornis

Taxon classificationAnimaliaColeopteraStaphylinidae

(Lacordaire, 1835)

C1164C98-66CD-56B0-B0BE-3DF38E0703E4

BOLD:ACF6186

Fig. 1A–H

##### Material

**(DNA barcoded specimens). Canada: Ontario**: Fergus, Centre Wellington District High School, 43.704, -80.358, Malaise trap, 3.V.2013, M. Cottrill (1, CBG); Guelph, Biodiversity Institute of Ontario, 43.528, -80.229, Malaise trap, 25.VII.2013, BIO Collections Staff (1, CBG); Rouge National Urban Park, west of Glen Rouge campground, 43.804, -79.146, marsh scrub along riverside, pitfall trap, 9.VI.2013, BIObus 2013 (1, CBG); Cambridge, rare Charitable Research Reserve, Preston Flats, 43.3908, -80.3747, grassy wetland, pitfall trap, 31.V.2015, BIO Collections staff (2, CBG); Peterborough, 44.318, -78.372, farm, malaise trap, B. McClenaghan (1, CBG).

##### Distribution.

**Origin**: Palaearctic (adventive in Nearctic). **Canada**: ON [new record].

##### Diagnosis.

*Amarocharaforticornis* may be easily recognized among the other Canadian species of the genus by the distal antennomeres, which are less than twice as wide as long. The species is also unique within the genus by having a distinct basal impression on abdominal tergite VI.

##### Bionomics.

In its native range, *A.forticornis* occurs in a variety of open and forested habitats, including forests, edges of waterways, grasslands, agricultural fields, and gardens (Assing 2002). It has been mostly collected by pitfall traps in the spring and summer, and then from flood debris in the cooler months of the year (Assing 2002). Assing (2002) suggested that beetles in flood debris were washed from some cryptic, subterranean microhabitat. Canadian specimens were collected in similar ways as in Europe.

##### Comments.

Newly reported as adventive in North America, from several localities in southern and eastern Ontario. It is native to the West Palaearctic and is known from most of Central Europe, Russian Central Territory, Armenia, and Georgia (Newton 2019).

**Figure 1.**
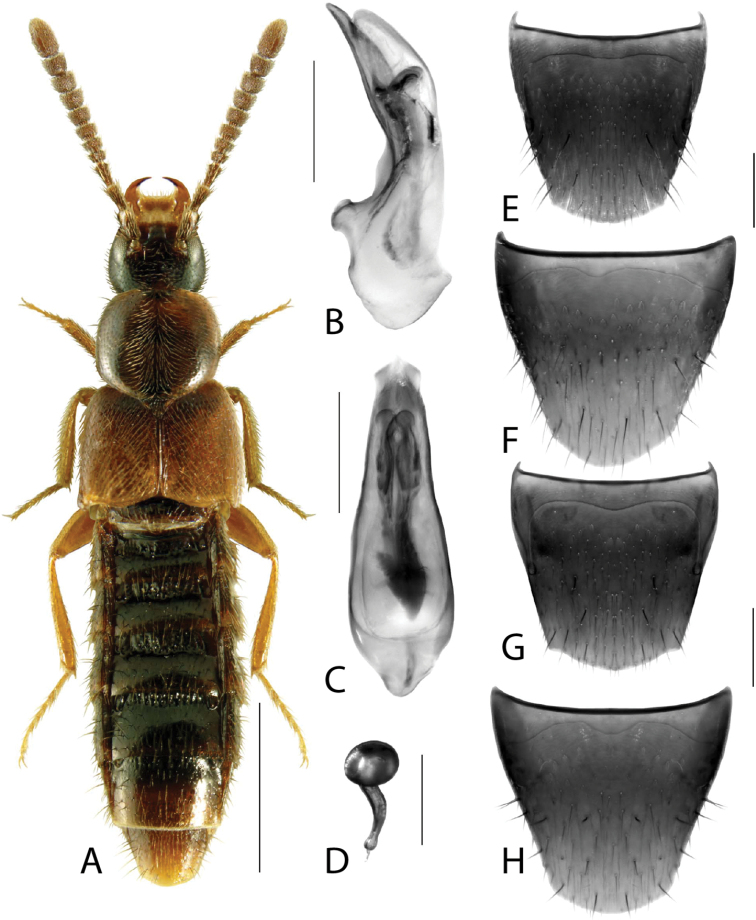
*Amarocharaforticornis* (Lacordaire) **A** habitus **B** median lobe of aedeagus in lateral view **C** median lobe of aedeagus in dorsal view **D** spermatheca **E** male tergite VIII **F** male sternite VIII **G** female tergite VIII **H** female sternite VIII. Scale bars: 1 mm (**A**); 0.2 mm (**B–H**).

### The key to Eastern Canadian *Amarochara* in Klimaszewski et al. (2018) should be modified as follows

**Table:** 

1A	Antennomere 10 only weakly transverse (Fig. 1A); abdominal tergite VI with distinct basal impression in addition to coarse punctures	***Amarocharaforticornis* (Lacordaire)**
–	Antennomere 10 strongly transverse, at least twice as wide as long (native species); abdominal tergite VI with, at most, coarse punctures at base	**1B**
1B	Pronotum with strong microsculpture and coarse, dense punctation, surface almost matte	***A.duryi* (Casey)**
–	Pronotum without microsculpture or with fine microsculpture, and with fine sparse to moderately dense punctation, surface moderately to highly glossy	**2**

### Tribe Oxypodini C.G. Thomson, 1859

#### Subtribe Microglottina Fenyes, 1918

##### Haploglossa nebulosa

Taxon classificationAnimaliaColeopteraStaphylinidae

(Casey, 1906)

FAA43CAB-1D19-5EB0-8D93-E6F624AA22F8

BOLD:ACK6454

Fig. 2A–H

###### Material

**(DNA barcoded specimens). Canada: Ontario**: Rouge National Urban Park, Toronto Zoo, 43.8223, -79.1897, forest, malaise trap, 21.V.2013, L. Attard and K. Greenham (1, CBG).

###### Distribution.

**Origin**: Nearctic. **Canada**: ON [new record]. **United States**: OK, PA.

###### Diagnosis.

*Haploglossanebulosa* may be easily distinguished from the other Nearctic species of the genus, *H.barberi* (Fenyes), by the bicolored elytra and fusiform body (Klimaszewski and Ashe 1991). Based on the shape of the spermatheca with its narrow capsule and broadly rounded apex, *H.nebulosa* may be most closely related to Palaearctic *H.marginalis* (Gravenhorst) as is suggested by barcode clustering. However, *H.nebulosa* can be readily distinguished by the pronotum, which is dark and paler only along the margins, while *H.marginalis* has broad pale areas laterally. *Haploglossanebulosa* was compared to Palaearctic *H.villosula* (Stephens) by Klimaszewski and Ashe (1991; as *H.pulla* (Gyllenhal)), but the species is quite different externally (much darker, finer pronotal punctation) and the spermatheca of the latter species is of the type with a large, rounded capsule.

###### Bionomics.

All members of *Haploglossa* are nidicolous, mostly in bird nests but also in mammal and ant nests (summarized by Staniec et al. 2010). Some species with well-known life histories appear to specialize on particular types of host nests, such as birds of prey (*H.picipennis* (Gyllenhal)) or bank swallows (*H.nidicola* (Fairmaire)) (Staniec et al. 2010). The genus is very rarely collected in North America. The Nearctic species *H.barberi* (Fenyes) was collected in long series from bank swallow nests (Klimaszewski and Ashe 1991). One specimen of *H.nebulosa* has been found in a rodent nest within a hollow tree (Klimaszewski and Ashe 1991) but bird and mammal nests have been poorly sampled in the Nearctic and more collecting is needed to determine the biology of the Nearctic *Haploglossa* (Brunke and Buffam 2018).

###### Comments.

The genus *Haploglossa* and *H.nebulosa* are newly reported from Canada, from a single locality in southern Ontario. The species is also known from Oklahoma and Pennsylvania, United States (Klimaszewski and Ashe 1991).

**Figure 2.**
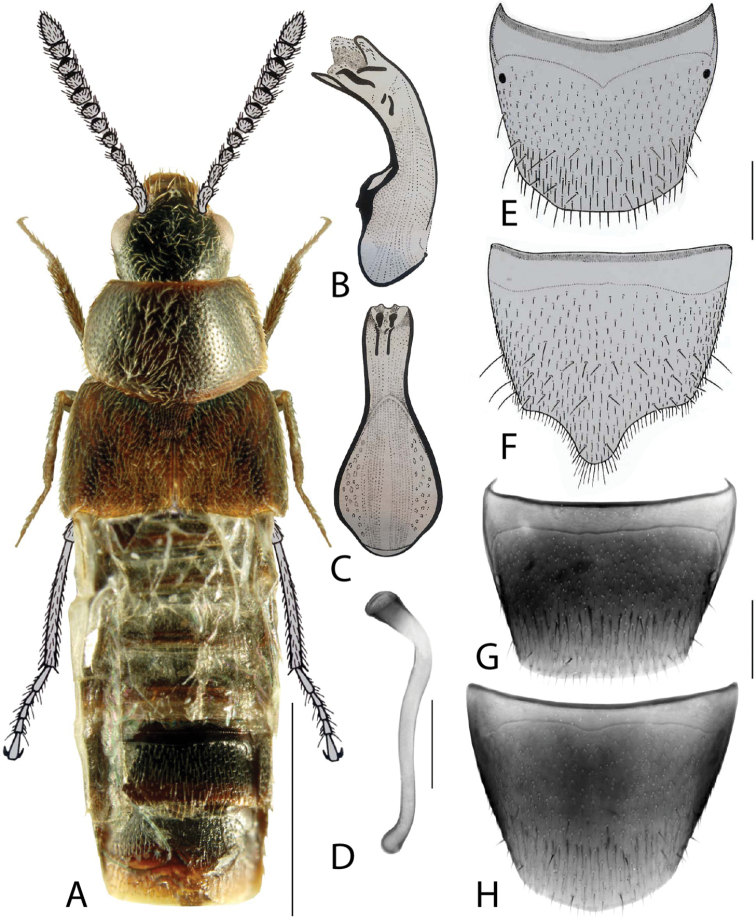
*Haploglossanebulosa* (Casey) **A** habitus **B** median lobe of aedeagus in lateral view (adapted from Klimaszewski and Ashe (1991)) **C** median lobe of aedeagus in dorsal view (adapted from Klimaszewski and Ashe (1991)) **D** spermatheca **E** male tergite VIII **F** male sternite VIII **G** female tergite VIII **H** female sternite VIII. Scale bars: 1 mm (**A**); 0.2 mm (**B–H**).

### The key to genera of Oxypodini in Eastern Canada in Klimaszewski et al. (2018) should be modified as follows

**Table:** 

8A	Pronotum strongly converging anteriad; posterolateral margin of elytra with strong sinuate emargination	**8B**
–	Pronotum not or, at most, weakly converging anteriad	**9**
8B	Pronotum with fine punctures, not clearly visible at moderate magnification, shape strongly transverse, ~ 1.5 × wider than long	***Crataraea* Thomson**
–	Pronotum with coarse punctures, clearly visible with low magnification, shape weakly transverse, no more than 1.4 × wider than long	***Haploglossa* Kraatz**

#### Subtribe Oxypodina C.G. Thomson, 1859

##### Hylota cryptica

Taxon classificationAnimaliaColeopteraStaphylinidae

Klimaszewski & Webster, 2016

05D18B83-114C-5324-8226-4A6086310487

BOLD:ACN2725

Fig. 3A–G

###### Material

**(DNA barcoded specimens). Canada: Ontario**: Guelph, Hanlon Preservation Park, 43.506, -80.213, mixed forest, dead wood and beating, 11.VI.2017, M. Pentinsaari (1, CBG); Hartington, Eel Lake Cottage, 44.5628, -76.553, Lindgren funnel, 12.VII.2017, G. Blagoev (1, CBG); Kawartha Lakes, 44.28, -78.529, farm, malaise trap, 5.V.2016, B. McClenaghan (1, CBG); Murphy’s Point Provincial Park, 44.7812, -76.2336, forest, malaise trap, 19.VI.2014, CBG Collections staff (1, CBG); **Newfoundland**: Terra Nova National Park, Blue Hill Road, 48.598, -53.9702, malaise trap, old balsam fir forest, 2.VII.2013, E. Perry (1, CBG).

###### Distribution.

**Origin**: Nearctic. **Canada**: AB, NB, NF [new record], ON [new record].

###### Bionomics.

Little is known about the microhabitat preferences of this species, but it likely occurs in in nests or cavities within trees as does *H.ochracea* (Klimaszewski et al. 2018).

###### Comments.

This recently described species, previously known from New Brunswick and Alberta (Klimaszewski et al. 2018) is newly recorded from Ontario and Newfoundland. It is likely to be widely distributed in North America east of the Rocky Mountains.

**Figure 3.**
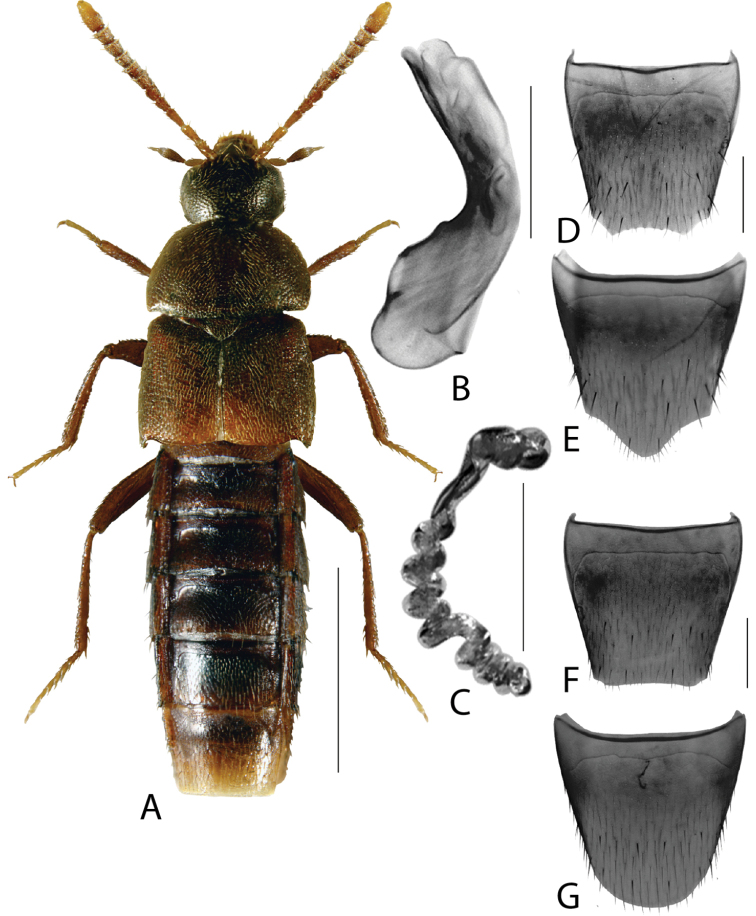
*Hylotacryptica* Klimaszewski & Webster **A** habitus **B** median lobe of aedeagus in lateral view **C** spermatheca **D** male tergite VIII **E** male sternite VIII **F** female tergite VIII **G** female sternite VIII. Scale bars: 1 mm (**A**); 0.2 mm (**B–G**). Illustrations after Webster et al. (2016).

##### Hylota ochracea

Taxon classificationAnimaliaColeopteraStaphylinidae

Casey, 1906

5F0DA124-3E2B-55A8-801C-6C32F923749D

BOLD:ABW9176

Fig. 4A–H

Stichoglossa (Dexiogyia) forticornis Strand, 1939, syn. nov.

###### Material

**(DNA barcoded specimens). Canada: Ontario**: Guelph, Dovercliffe Road, 43.51, -80.254, backyard, compost and mouldy hay piles, 6.VI.2018, M. Pentinsaari (3, CBG); Guelph, Hanlon Preservation Park, 43.51, -80.221, mixed forest, at UV light, 30.VI.2018, M. Pentinsaari (1, CBG); Whitby, Julie Payette Public School, Malaise trap, 43.886, -78.934, 22.IV.-03.V.2013, Z. Turner (1, CBG). **Quebec**: Montreal, Montreal Botanical Garden, 45.559, -73.566, Malaise trap, 24.VII-02.VIII.2014, M. Larrivee (1, CBG). **Finland**: Al: Lemland, Äspholm, 60.0675, 19.9583, 9.X.2011, M. Pentinsaari (1, ZMUO); Al: Lemland, Nåtö, 60.046, 19.981, 26.VI.2014, M. Pentinsaari (2, ZMUO); N: Sipoo, Sipoonkorpi, 60.304, 25.202, window trap, 2.VIII.2013, S. Karjalainen and P. Martikainen (1, ZMUO).

###### Distribution.

**Origin**: Nearctic (adventive in Europe). **Canada**: NB, NS, NT, ON, QC, SK. **United States**: NY, VT.

###### Bionomics.

*Hylotaochracea* is strongly associated with bird nests in forested habitats. It has also been collected from artificial analogs such as a pigeon coup, manmade nest boxes, and a plastic composter bin containing carrion and decaying vegetables (Klimaszewski et al. 2018). The specimens recently collected in Ontario, Canada were found in compost and at UV light.

###### Comments.

*Hylotaochracea*, a widespread Nearctic species (Klimaszewski et al. 2018), is newly reported from the Palaearctic region and had been previously known from Finland, Denmark, Germany, Norway, Sweden, and Switzerland (Lundberg 2006; Schülke and Smetana 2015; Newton 2019) under the synonym *Dexiogyiaforticornis*. *Hylota* is also a new genus record for the Palaearctic region.Nearctic *Hylotaochracea* and Palaearctic *D.forticornis* share a BIN and do not form separate clusters. One of the DNA barcode haplotypes is shared between Finnish and Canadian specimens. Nearctic and Palaearctic populations also have identical male and female genitalia. Based on its specialization on microhabitats in forests, we do not consider *H.ochracea* to be a naturally occurring Holarctic species. Holarctic beetles are generally those that occur north of the treeline and have crossed treeless Beringia in the last 2.8 Mya (reviewed in Brunke et al. 2020). *Hylotaochracea* may have been introduced to the Palaearctic region with the nest material of poultry or domestic pigeons, or with another form of decaying plant matter. A similar situation has occurred with the bird nest-associated staphylinid *Bisniuspalmi* (Smetana), which was originally described from Italy but later found to be a native Nearctic species (Smetana 1995).

With the above synonymy, the genus *Dexiogyia* is now known only from externally similar sister species *D.angustiventris* (Casey) (Nearctic) and *D.corticina* (Erichson) (West Palaearctic), plus Afrotropical *D.congoensis* (Scheerpeltz). As in the former *D.forticornis*, *D.congoensis* is probably misplaced due to superficial similarity. *Hylota* is readily separated from *Dexiogyia* by the shape of the pronotum, which is strongly convergent anteriad, such that its apical width is subequal to the width of the head. In *Dexiogyia*, the head is distinctly narrower than the pronotum.

**Figure 4.**
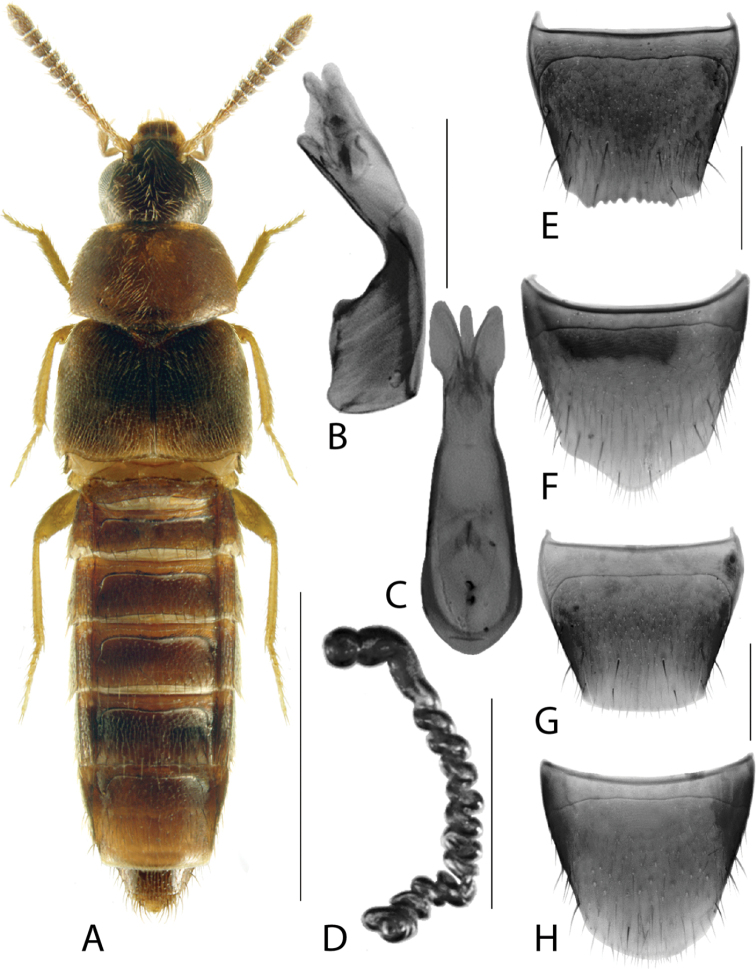
*Hylotaochracea* Casey **A** habitus **B** median lobe of aedeagus in lateral view **C** median lobe of aedeagus in dorsal view **D** spermatheca **E** male tergite VIII **F** male sternite VIII **G** female tergite VIII **H** female sternite VIII. Scale bars: 1 mm (**A**); 0.2 mm (**B–H**). Illustrations after Klimaszewski et al. (2018), reproduced with permission.

##### Isoglossa

Taxon classificationAnimaliaColeopteraStaphylinidae

Casey, 1893

AD374A1E-92D0-5E5E-8511-F4652EB0CEC0

Rheobioma Casey, 1906; Klimaszewski and Pelletier (2004), syn. of Neoisoglossa
Athetalia Casey, 1910 (in part); Klimaszewski and Pelletier (2004) syn. of Neoisoglossa
Neoisoglossa Klimaszewski & Pelletier, 2004; Gouix and Klimaszewski (2007), syn. of Isoglossa, unnecessary replacement name; Klimaszewski et al. (2020) as valid genus, incorrectly attributed to Casey (1893).

###### Comments.

In Klimaszewski et al. (2020), *Neoisoglossa* was incorrectly attributed to Casey but was actually proposed by Klimaszewski and Pelletier (2004), apparently as an unnecessary replacement name for *Isoglossa*Casey 1893. The previous treatment of these generic names and two other synonyms in the catalog of Gouix and Klimaszewski (2007) is correct and followed here. Blackwelder (1952) was wrong and there is no *Isoglossa* Newman that preoccupied Casey’s name, so *Isoglossa* Casey stands as valid with *Neoisoglossa* as a synonym.

##### Isoglossa triangularis

Taxon classificationAnimaliaColeopteraStaphylinidae

Klimaszewski, Brunke & Pentinsaari sp. nov.

0A48DBD4-50F3-56F5-813B-2D277A51D955

http://zoobank.org/A8A0402E-2950-4394-B9CF-25E3DD629804

BOLD:ACU5806

Fig. 5A–H

###### Type material.

***Holotype*.** (male): Canada, British Columbia, Prince George, Nukko Lake Elementary EQP-CLL-574, 54.0831°N, 122.988°W, 764 m asl, Holly Sapun 04/20/2015 to 05/08/2015, Barcode of life, DNA voucher specimen, Sample ID: BIOUG22036-B02, Process ID: SMTPM2682-15 (CNC). ***Paratypes*** (3, CBG): Canada, British Columbia, Prince George, Nukko Lake Elementary EQP-CLL-574, 54.0831°N, 122.988°W, 764 m asl, Holly Sapun 04/20/2015 to 05/08/2015, Barcode of life, DNA voucher specimen, Sample ID: BIOUG22036-B07, Process ID: SMTPM2682-15 (1 male, CBG); same label data except: Sample ID: BIOUG22035-H08, Process ID: SMTPM2665-15 (1 female, CBG); Sample ID: BIOUG22036-A04, Process ID: SMTPM2672-15 (1 female, CBG).

###### Etymology.

The species epithet refers to the remarkably separated triangular apex of the median lobe of the aedeagus, distinguishing it from all other members of the *Ocalea* group.

###### Distribution.

**Origin**: Nearctic. **Canada**: BC.

###### Diagnosis.

**Figure 5.**
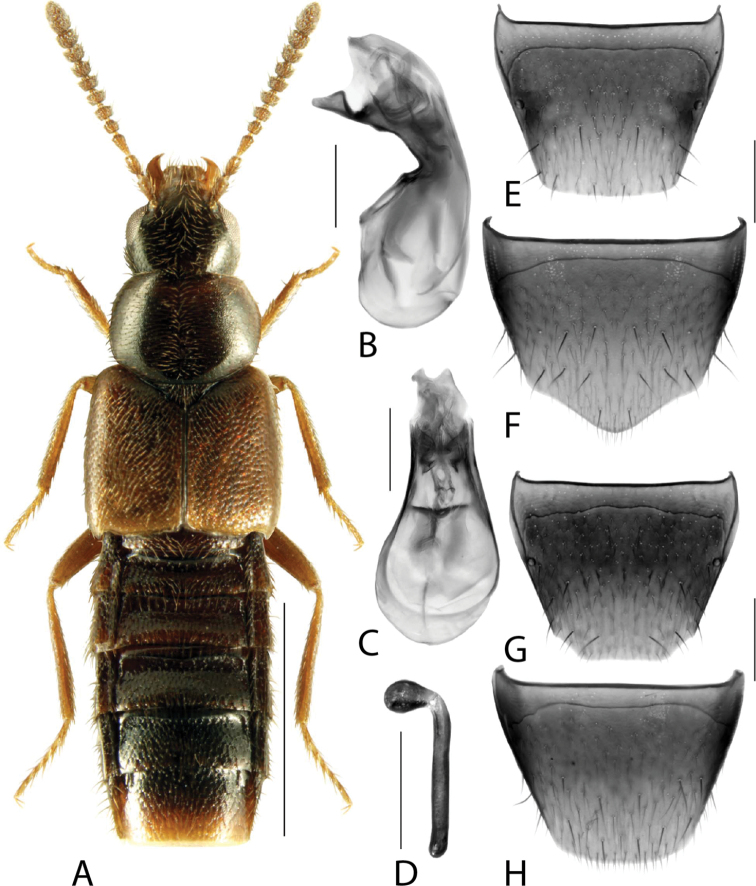
*Isoglossatriangularis* Klimaszewski, Brunke & Pentinsaari, sp. nov. **A** habitus **B** median lobe of aedeagus in lateral view **C** median lobe of aedeagus in dorsal view **D** spermatheca **E** male tergite VIII **F** male sternite VIII **G** female tergite VIII **H** female sternite VIII. Scale bars: 1 mm (**A**); 0.2 mm (**B–H**).

*Isoglossatriangularis* can be easily distinguished from all Nearctic species of the *Ocalea* group of genera by a combination of the strongly transverse and sparsely punctate pronotum, transverse antennomere 4, distinct triangular apex of the median lobe in lateral view (Fig. 5B), and distinct and simple ‘walking cane’ shape of the spermatheca (Fig. 5D).

###### Description.

Body length 3.0–3.3 mm, dark brown with elytra, antennomeres 1–2 or 1–3, legs and apical part of abdomen yellow-brown, forebody moderately glossy and abdomen strongly so (Fig. 5A); antenna moderately stout, antennomere 4 slightly transverse, antennomeres 5–10 strongly transverse, terminal antennomere ca. as long as two preceding ones combined; pronotum transverse (width/length ratio = 1.6), impressed medially at base, lateral edges evenly arcuate, length ratio of base to apex 1.2 ×, punctures fine and sparse, distance between punctures ~ 3 × diameter of a puncture, space between punctures with faint isodiametric microsculpture, pubescence directed laterad from midline of disc forming arcuate lines on both sides; elytra transverse (width/length ratio = 1.3), 1.5 × as long as pronotum; abdomen arcuate laterally and gradually narrowing toward apex. MALE. Tergite VIII broadly arcuate apically (Fig. 5E); sternite VIII with apical part broadly triangularly produced (Fig. 5F); median lobe of aedeagus in lateral view with narrowly elongate crista apicalis at base of bulbus, tubus moderately long, strongly produced ventrally, apex narrowly triangular constricted baso-dorsally in lateral view (Fig. 5B), internal sac structures not pronounced (Fig. 5B, C). FEMALE. Tergite VIII truncate apico-medially (Fig. 5G); sternite VIII arcuate apically (Fig. 5H); spermatheca with capsule approximately spherical with short neck, stem narrow, long and straight (Fig. 5D).

###### Bionomics.

The specimens were collected in a Malaise trap on an open field surrounded by mixed forest.

###### Comments.

Based on a combination of small size (< 4.5 mm), superficial, meshed microsculpture, sparse pronotal punctation, with punctures separated by more than two puncture diameters, pronotum transverse, shorter and narrower than elytra, and the transverse antennomeres 5–10, *I.triangularis* keys to genus *Isoglossa* Casey in Klimaszewski and Pelletier (2004). However, barcode sequences of this species do not cluster with *Isoglossaagnita* but rather form a cluster with *Gennadotacanadensis* and the species of *Neothetalia* which bear a spermatheca with broad, circular loops, similar to those of *Gennadota*. *Isoglossatriangularis* has a simple spermatheca with a long straight stem and is not externally similar to these taxa (see above), and the barcode divergence between these species and *I.triangularis* is 11–12%. It is likely that *I.triangularis* belongs in a separate genus, but this is outside of the scope of this study. We here place *I.triangularis* tentatively in *Isoglossa* as not to disturb the existing morphological diagnoses of the genera and identification keys (e.g., Klimaszewski and Pelletier 2004; Klimaszewski et al. 2020), pending generic revision of the *Ocalea* group.

##### Parocyusa rubicunda

Taxon classificationAnimaliaColeopteraStaphylinidae

(Erichson, 1837)

9525D491-2F0D-5740-B065-B3C850EB878D

Fig. 6A–D

Tachyusa rubicunda Erichson, 1837
Chilopora americana Casey, 1906, syn. nov.
Tetralaucopora americana : Klimaszewski et al. 2018 (as valid species)
Parocyusa americana : Assing 2021 (possible synonym of P.rubicunda)

###### Material

**(DNA barcoded specimens). Austria**: Innervillgraten, Arntal, 46.8362, 12.3348, 1580 m, 22.VIII.2010, F. Koehler and J. Koehler (2, ZSM). **Canada: Ontario**: Northumberland County, Peter’s Woods Protected Natural Area, 44.124, -78.039, under rock in streambed,12.IX.2011, A. Brunke and S. Paiero (1, DEBU); Crieff Bog, 3 km W Puslinch, sedge meadow, 26.VI.1987, D. Blades (1, DEBU). **United States: Connecticut**: East Hartford, Two Rivers Magnet Middle School, 41.757, -72.655, 4.VI.2005, J. DeWaard (1, CBG).

###### Additional non-barcoded material.

**Canada: Ontario**: Ancaster, 21.X.1967 (1, CNC); Rondeau Prov. Pk., Tulip Tree Trail, *Carex* and moss on logs in pond, 5.VI.1985, A. Davies and J.M. Campbell (1, CNC); **Quebec**: Montreal, 20.IX.1969, E.J. Kiteley, 1 (CNC); Mt. Orford Park, 20.IX.-11.X.1972, Dondale and Redner, 1 (CNC).

###### Distribution.

**Origin**: West Palaearctic (adventive in North America). **Canada**: BC, ON, QC, NB, NF. **United States**: CT, NY, PA.

###### Bionomics.

In North America, most specimens of this species have been collected from near water, including a sandy creek bank, in a dried streambed and in moss near the splash zone of a waterfall (Klimaszewski et al. 2018). Nearctic populations of this species are only known from female specimens and the species may be parthenogenetic in North America. In its native distribution, the northern and northwestern populations are also parthenogenetic (Assing 2021) and most likely represent the source population for the Nearctic introduction.

###### Comments.

*Parocyusarubicunda* is a widespread West Palaearctic species (Europe, European Russia, Turkey, Georgia, Iran, Kazakhstan, Kyrgyzstan, Tajikistan, Uzbekistan) (Assing 2021). It is confirmed as established in the Nearctic region and had been previously known from North America under the synonym *Tetralaucoporaamericana* (Casey) (Klimaszewski et al. 2018). Assing (2021) recently reported this species from BC and treated *T.americana* as a tentative synonym based on the results presented in this paper.

Although all available sequences of this species are partial (382–407 bp) and a BIN has not been established as that would require at least one founding member with a minimum sequence length of 500 bp, Nearctic and Palaearctic sequences form a distinct cluster with only a single variable nucleotide site. External morphology and that of the spermatheca are identical. As spermathecae are of generally poor diagnostic value (especially the distal part) in *Parocyusa* (Assing 2021), the barcode evidence was quite critical for the resolution of this issue. Based on this evidence and a distribution centered around populated areas in northeastern and western North America, we here consider this species to be adventive in the Nearctic region. At the moment, it is not yet possible to determine whether the population in BC is a separate introduction from the northeastern population.

Recently, Assing (2021) revalidated *Parocyusa* as a genus separate from *Tectusa* after the discovery that *Tectusa* was not a monophyletic group. The type species of *Parocyusa* was found to be congeneric with that of *Tetralaucopora*, and the latter became a junior synonym of the former.

**Figure 6.**
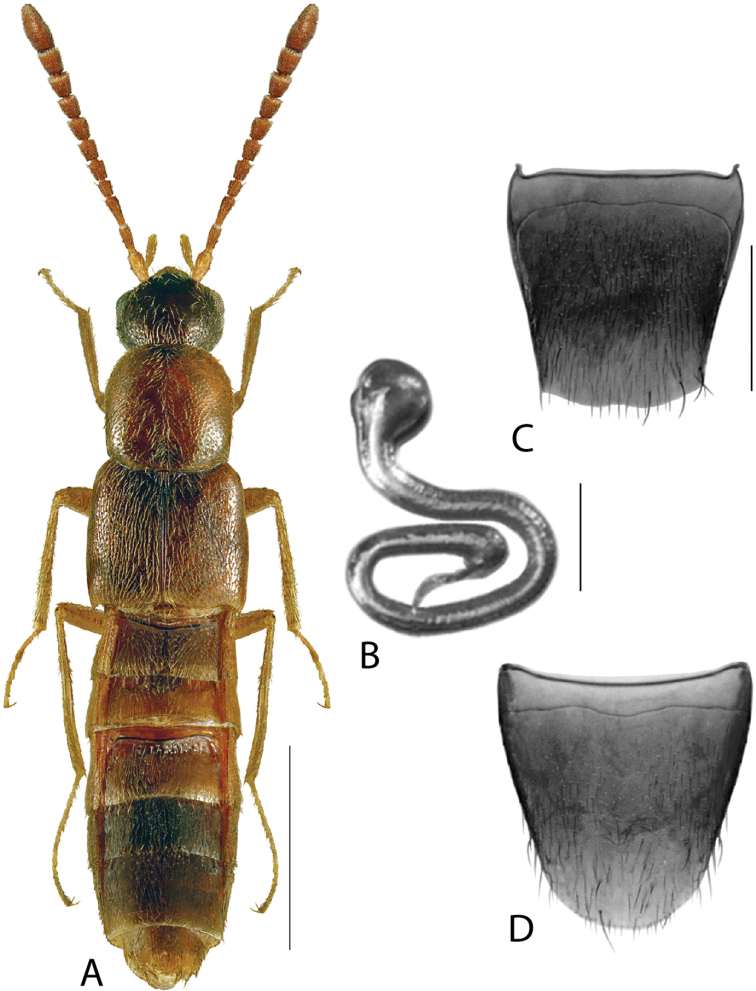
*Parocyusarubicunda* (Erichson) **A** habitus **B** spermatheca **C** female tergite VIII **D** female sternite VIII. Scale bars: 1 mm (**A**); 0.2 mm (**B–D**). Illustrations after Klimaszewski et al. (2016c).

Tribe Tachyusini C.G. Thomson

### Revised key to the Canadian genera of Tachyusini

Adapted from Klimaszewski et al. 2018.

**Table:** 

1	Elytra at humerus only slightly broader than pronotum at base (Figs 7, 8); impressions of abdominal tergites shallow, with punctation similar to that of disc (Figs 7, 8)	**2**
–	Elytra at humerus distinctly broader than pronotum at base (Figs 9–13); impressions of abdominal tergites with at least a few coarse punctures and glossy areas, punctation distinctly different from that of disc (Figs 9–13)	**3**
2	Pronotum with pubescence directed straight posteriad; hind tarsus subequal in length to hind tibia or longer (Fig. 8)	***Brachyusa* Mulsant & Rey**
–	Pronotum with pubescence directly posteriolaterad from midline; hind tarsus shorter, slightly longer than half the length of hind tibia or shorter (Fig. 7)	***Paradilacra* Bernhauer**
3	Abdomen clavate, at base distinctly narrower than head (Fig. 9); tergite III ca. as long as wide or longer; tergal impressions with median carina (Fig. 9)	***Tachyusa* Erichson**
–	Abdomen at most slightly constricted at base, subequal to or wider than head (Figs 10–13); tergite III ca. twice as wide as long or wider; tergal impressions never with median carina (Figs 10–13)	**4**
4	Abdomen at base elongate and moderately constricted, ca. as wide as head (Figs 10–12); tergite III, at most, twice as wide as long; tergal impressions deep and sharply delineated from strongly convex disc (Figs 10–12)	***Dasygnypeta* Lohse, sensu nov.**
–	Abdomen at base at most slightly constricted, wider than head (Fig. 13); tergite III strongly transverse, ~ 2.5 × wider than long or wider; tergal impressions shallower, gradually sloping to disc at base (Fig. 13)	***Gnypeta* Thomson**

#### Paradilacra densissima

Taxon classificationAnimaliaColeopteraStaphylinidae

(Bernhauer, 1909)

E571F2B1-45E2-5131-951F-93DE5877604D

BOLD:ACF7668

Fig. 7A–H

Atheta (Paradilacra) densissima Bernhauer, 1909
Gnypeta saccharina Klimaszewski & Webster, 2008, syn. nov.

##### Material

**(DNA barcoded specimens). Canada: Alberta**: Waterton Lakes National Park, Highway 6 pulloff, 49.065, -113.779, 1569 m, intercept trap, montane forest, 27.VI.2012, BIOBus 2012 (1, CBG); **British Columbia**: 10 km W Kamloops, New Afton Mine, Wetland Protected Area, 50.662, -120.504, 702 m, malaise trap, 22.VIII.2013, C. Simon (1, CBG); **New Brunswick**: York Co., Fredericton at Saint John River, 45.959, -66.625, margin of river in flood debris, 7.VII.2005, R.P. Webster [note: paratype of *G.saccharina*] (1, LFC); **Ontario**: Guelph, University of Guelph Arboretum, 43.53, -80.21, 12.VI.2019, M. Pentinsaari (1, CBG).

##### Distribution.

**Origin**: Nearctic. **Canada**: AB, BC, NB, ON [new record], SK [new record]. **United States**: CA, MT, NV, ND, OR, UT.

##### Bionomics.

This species has been collected from various wetland microhabitats including the edges of lakes, rivers, and a beaver pond (Gusarov 2003a).

**Figure 7.**
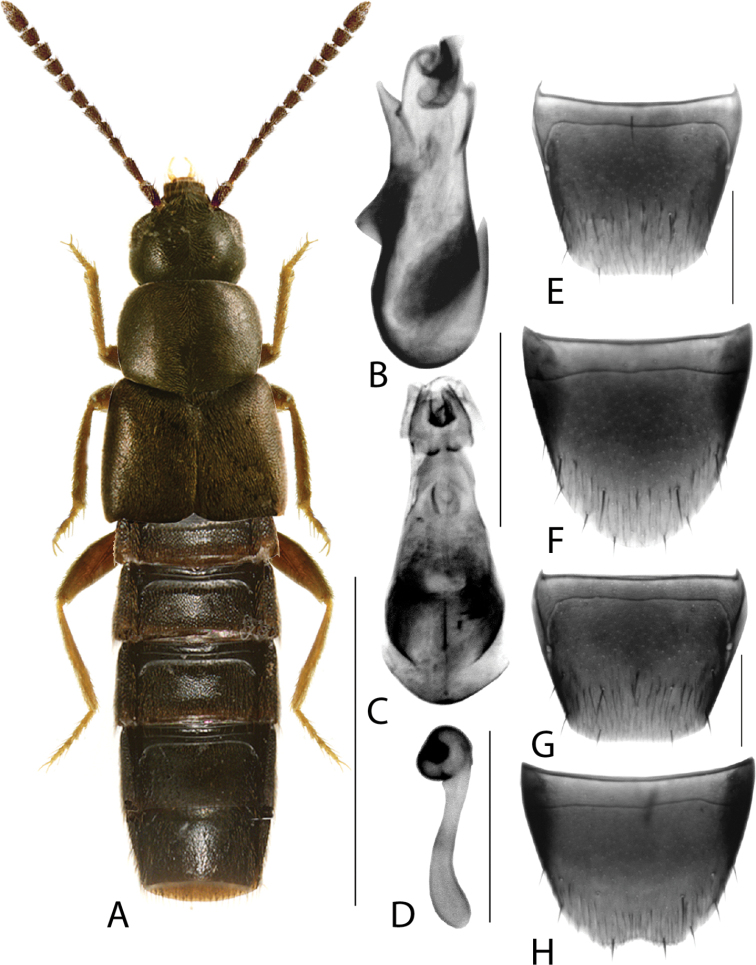
*Paradilacradensissima* (Bernhauer) **A** habitus **B** median lobe of aedeagus in lateral view **C** median lobe of aedeagus in dorsal view **D** spermatheca **E** male tergite VIII **F** male sternite VIII **G** female tergite VIII **H** female sternite VIII. Scale bars: 1 mm (**A**); 0.2 mm (**B–H**). Illustrations after Klimaszewski et al. (2018), used with permission.

##### Comments.

*Paradilacradensissima* and the genus *Paradilacra*, widespread in western and central North America (Gusarov 2003a), are newly reported from SK (records in Klimaszewski et al. 2016a, as *G.saccharina*) and eastern North America based on records from NB (Klimaszewski et al. 2008) and ON (this study), including one sequenced paratype of synonym *Gnypetasaccharina*. Under the present concept, only one widespread species of this genus is known.

**Figure 8.**
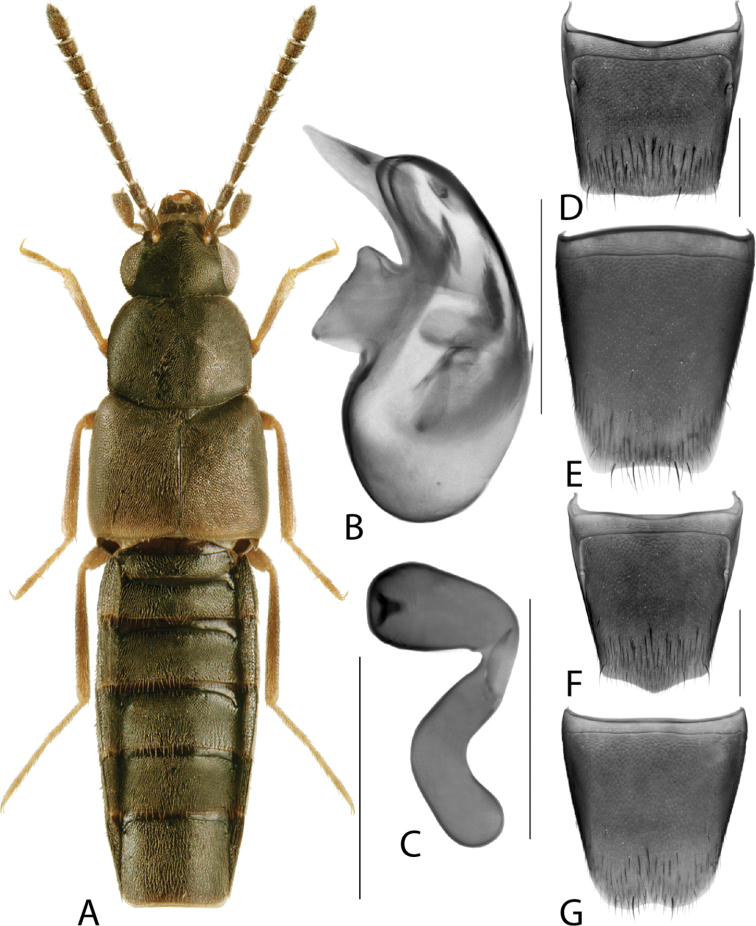
*Brachyusahelenae* (Casey) **A** habitus **B** median lobe of aedeagus in lateral view **C** spermatheca **D** male tergite VIII **E** male sternite VIII **F** female tergite VIII **G** female sternite VIII. Scale bars: 1 mm (**A**); 0.2 mm (**B–G**). Illustrations after Klimaszewski et al. (2016a).

###### *Dasygnypeta* Lohse, 1974, sensu nov.

**Figure 9.**
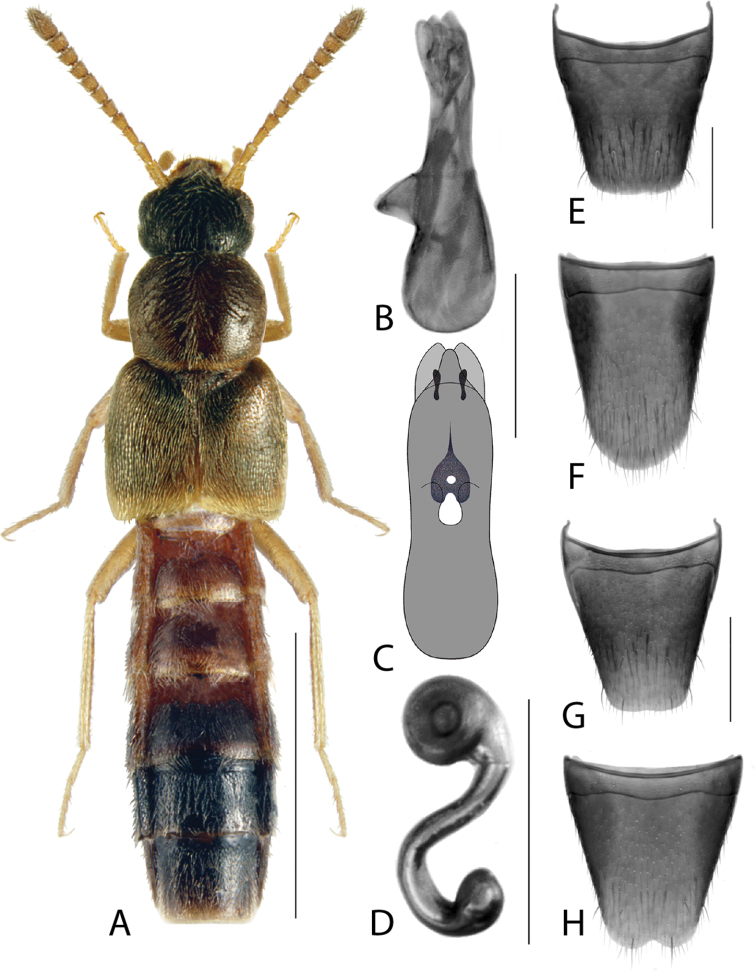
*Tachyusaobsoleta* Casey **A** habitus **B** median lobe of aedeagus in lateral view **C** median lobe of aedeagus in dorsal view (adapted from Paśnik 2006) **D** spermatheca **E** male tergite VIII **F** male sternite VIII **G** female tergite VIII **H** female sternite VIII. Scale bars: 1 mm (**A**); 0.2 mm (**B–H**). Illustrations after Klimaszewski et al. (2018), used with permission.

**Figure 10.**
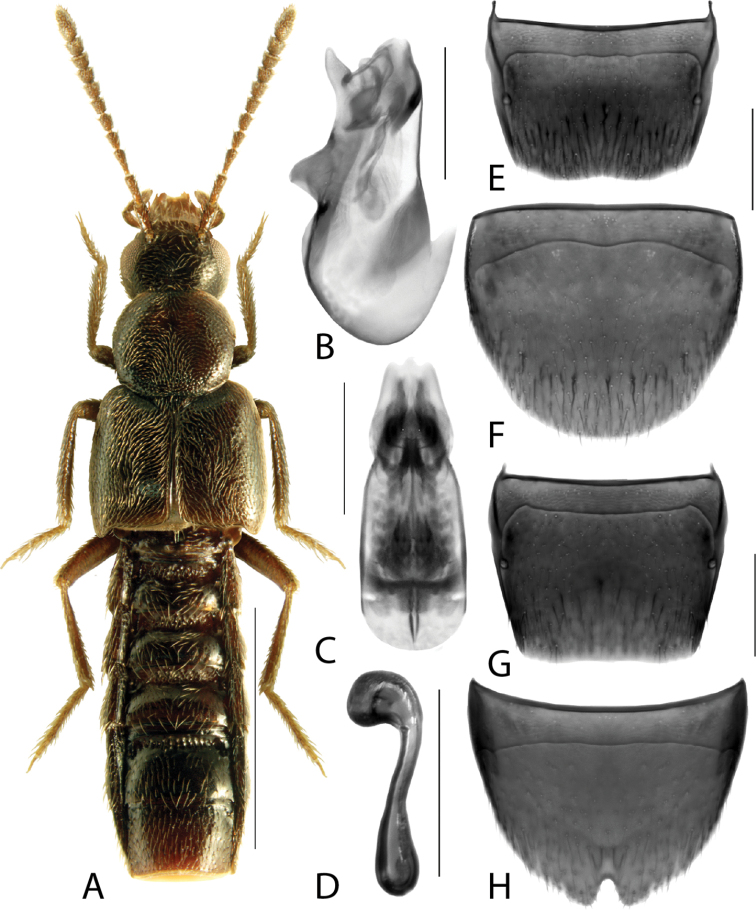
*Dasygnypetabaranowskii* (Klimaszewski) **A** habitus **B** median lobe of aedeagus in lateral view **C** median lobe of aedeagus in dorsal view **D** spermatheca **E** male tergite VIII **F** male sternite VIII **G** female tergite VIII **H** female sternite VIII. Scale bars: 1 mm (**A**); 0.2 mm (**B–H**). Illustrations after Klimaszewski et al. (2020), used with permission.

**Figure 11.**
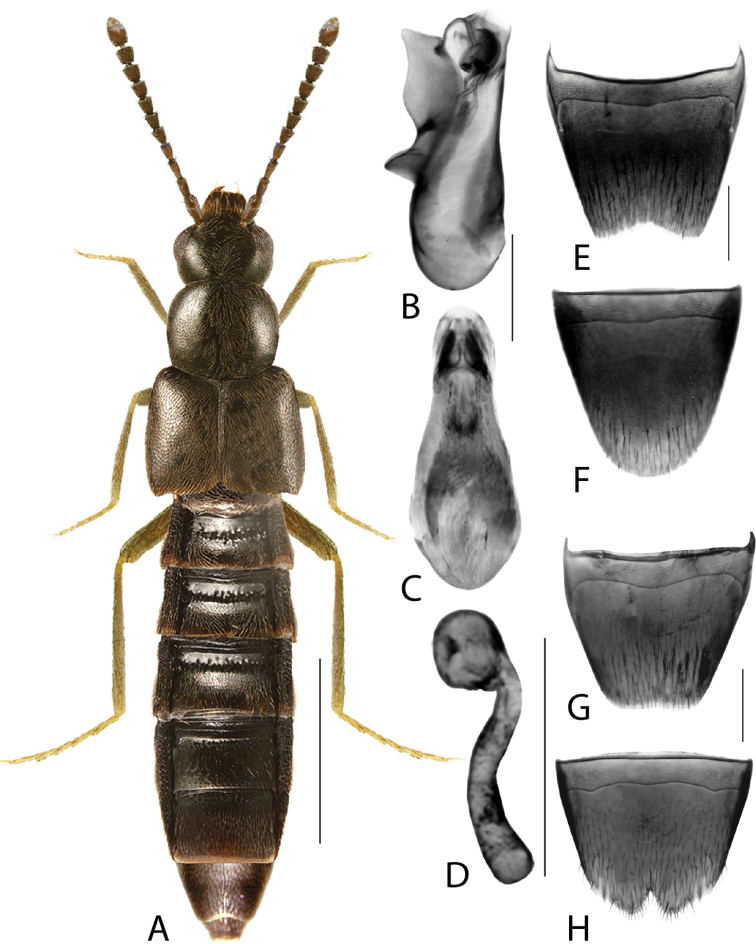
*Dasygnypetanigrella* (LeConte) **A** habitus **B** median lobe of aedeagus in lateral view **C** median lobe of aedeagus in dorsal view **D** spermatheca **E** male tergite VIII **F** male sternite VIII **G** female tergite VIII **H** female sternite VIII. Scale bars: 1 mm (**A**); 0.2 mm (**B–H**). Illustrations after Klimaszewski et al. (2018), used with permission.

**Figure 12.**
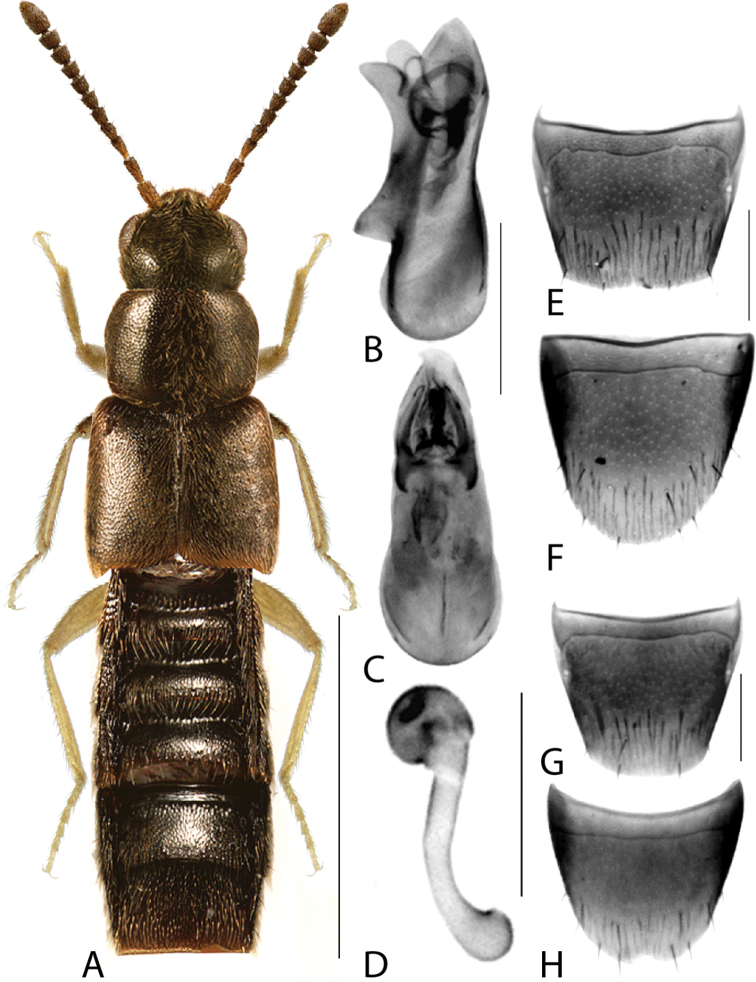
*Dasygnypetavelata* (Erichson) **A** habitus **B** median lobe of aedeagus in lateral view **C** median lobe of aedeagus in dorsal view **D** spermatheca **E** male tergite VIII **F** male sternite VIII **G** female tergite VIII **H** female sternite VIII. Scale bars: 1 mm (**A**); 0.2 mm (**B–H**). Illustrations after Klimaszewski et al. (2018), used with permission.

In his key to the genera of Tachyusini, Paśnik (2010) distinguished *Dasygnypeta*, with its single Palaearctic species *Dasygnypetavelata* (Erichson), from the Nearctic genera by the following features: the narrow and slender abdomen with base approximately as wide as head, the ‘very deep’ tergal impressions, abdominal pubescence of tergites III–V directed posteriad, basal segment of metatarsus shorter than following two segments. Through an analysis of barcode data, we have discovered that the recently described *Gnypetaminuta* Klimaszewski & Webster is a synonym of *D.velata* (see below). A re-examination of other Nearctic *Gnypeta* species revealed two others that are closely related to *D.velata*: *G.baranowskii* Klimaszewski, and *G.nigrella* (LeConte). Their morphological divergence from other *Gnypeta* was represented by an earlier placement in the ‘Nigrella species group’ of *Gnypeta* by Klimaszewski et al. (2008), together with *G.saccharina* (now a synonym of *Paradilacradensissima*). In corroboration with morphology, DNA barcodes of *D.velata* and *G.nigrella* form sister clusters (sequences of *G.baranowskii* not available). Transfer of these two *Gnypeta* species to *Dasygnypeta* required a new concept for this genus as most of the distinguishing features were apomorphies of *D.velata* or found not to be of diagnostic value due to variability or overlap with other genera. Here we distinguish members of *Dasygnypeta* by their characteristic abdomen (Figs 10–12): base of abdomen ca. as wide as head; basal half of abdomen elongate, tergite III (first visible) only moderately transverse, ~ 2 × as wide as long (at least 2.5 × in *Gnypeta*); tergites III–V with very deep basal impressions, each creating strongly convex areas on the disc. Members of *Dasygnypeta* could be confused with *Tachyusa*, which also bears an elongate basal abdomen, but in the latter genus tergites III–V are far more elongate and the abdominal base is narrower than the head (Fig. 9). *Dasygnypetanigrella* was even originally described by LeConte (1863) in *Tachyusa*, likely based on this similarity.

#### Dasygnypeta baranowskii

Taxon classificationAnimaliaColeopteraStaphylinidae

(Klimaszewski, 2020) comb. nov.

07B2AE1C-D445-5110-AA12-4E67C8ECBA3B

Fig. 10A–H

Gnypeta baranowskii Klimaszewski, 2020

##### Distribution.

**Origin**: Nearctic. **Canada**: BC.

##### Bionomics.

The type series was collected by sifting litter (Klimaszewski et al. 2020).

##### Comments.

We here transfer this species to *Dasygnypeta* sensu nov. based on morphology illustrated by Klimaszewski et al. (2020). This recently described western species is most similar to eastern *D.nigrella* based on the moderately elongate antennae, more robust body and the distinctive deep emargination of female sternite VIII. However, it is easily distinguished by the coarser pronotal punctation, and male and female genitalia (Fig. 10). The aedeagus of *D.baranowskii* is superficially similar to *P.densissima* but these taxa are externally quite different.

#### Dasygnypeta nigrella

Taxon classificationAnimaliaColeopteraStaphylinidae

(LeConte, 1863) comb. nov.

E1AE07D9-4C82-5A3F-BDBF-05959CE93F6E

BOLD:ACS6831

Fig. 11A–H

Tachyusa nigrella LeConte, 1863
Gnypeta nigrella : Klimaszewski et al. 2008

##### Material

**(DNA barcoded specimens). Canada: New Brunswick**: York Co., Fredericton at St. John River, 45.959, -66.625, margin of river in drift (mostly maple seeds), 4.VII.2004, R.P. Webster (1, LFC).

##### Additional non-barcoded material.

**Canada: Manitoba**: 5 miles SW of Shilo, 5.VI.1958, J.F. McAlpine (1, CNC); **Quebec**: Montreal, 30.VIII.1968, E.J. Kiteley (1, CNC); Montreal 14.VI.1972, E.J. Kiteley (1, CNC); Berthierville, 5.VI.1976, E.J. Kiteley (3, CNC); Kazabazua, 15.VIII.1968, R.C. Lawrence (3, CNC); Wakefield, 4.VI.1930, W.J. Brown (1, CNC); Drummondville, 18.VII.1977, river mudflat, L. LeSage (5, CNC).

##### Distribution.

**Origin**: Nearctic. **Canada**: MB [new record], NB, NF, ON, QC [new record]. **United States**: IL, MA, MD, NJ, NY, PA, VT, WV.

##### Bionomics.

Collected along the edge of a variety of running and standing water-based habitats.

##### Comments.

We here transfer this species to *Dasygnypeta* sensu nov. based on morphology and close clustering of DNA barcode sequences with *D.velata*. *Dasygnypetanigrella* is a widespread species in eastern North America and is here newly reported from Manitoba and Quebec.

#### Dasygnypeta velata

Taxon classificationAnimaliaColeopteraStaphylinidae

(Erichson, 1837)

1367A6AB-1432-5D19-BF2F-3ADF13AEBD27

BOLD:ACZ0581

Fig. 12A–H

Homalota velata Erichson, 1837
Gnypeta minuta Klimaszewski & Webster, 2008, syn. nov.

##### Material

**(DNA barcoded specimens). Germany**: Thuringia, Ufergehoelze am Speicher Loessau, 50.5665, 11.894, 460 m, 1.I.2013, GBOL-Team ZFMK (1, ZFMK); Thuringia, NE, Freibad, Werraufer, 50.9768, 10.0963, 20.X.2014, GBOL-Team ZFMK (1, ZFMK). **United States: Alaska**: Selawik NWR, Kugarak River, 66.561, -158.996, mud bank, shore washing, 23.VI.2010, D.S. Sikes (3, UAM).

##### Additional non-barcoded material.

**Canada: Manitoba**: 5 miles SW of Shilo, 5.VI.1958, J.F. McAlpine (2, CNC); **Northwest Territories**: Inuvik, 24.VI.1972, A. Smetana (1, CNC). **United States: Alaska**: Hess Creek, mi. 24 Wales Hwy, 1.VII.1978, J.M. Campbell and A. Smetana (1, CNC); Kenai Peninsula, Anchor River at Hwy 1, 450’, 4.VI.1978, A. Smetana and E. Becker (1, CNC); mi. 1259 Alaska Hwy, 7.VII.1968, J.M. Campbell and A. Smetana (1, CNC).

##### Distribution.

**Origin**: Holarctic. **Canada**: MB [new record], NB, NF, NT, SK. **United States**: AK.

##### Bionomics.

Nearctic specimens have been collected most frequently along the margins of running water but also along the margins of a forest pool (Klimaszewski et al. 2018).

##### Comments.

*Dasygnypetavelata* is newly reported from North America and was previously known in the Nearctic region under the synonym *Gnypetaminuta* (Klimaszewski et al. 2018). We here newly record this taxon from MB. *Dasygnypetavelata* is here considered a Holarctic species as it has a broad, transpalaearctic distribution (Europe to Siberia; Newton 2019), occurs along rivers and has been collected north of the treeline in Alaska.

This species has been collected together with *D.nigrella* in southern Manitoba (see above). The barcode sequences of the specimens from Alaska are all partial (386 to 407 bp), but the overlapping parts of the sequences are identical to the two German sequences.

#### Gnypeta impressicollis

Taxon classificationAnimaliaColeopteraStaphylinidae

Klimaszewski, Brunke & Pentinsaari sp. nov.

CDA6F43E-AA37-5C10-9DEA-6ABBD17D3B65

http://zoobank.org/C6DC72D8-A182-43D3-9691-A352F0AF5D0E

BOLD:ADH7347

Fig. 13A–H

##### Type material.

***Holotype*.** (male, CNC): **Canada**, Ontario, Hartington, Eel Lake, South Frontenac, Paul Hebert’s cottage property, 44.563°N, 76.549°W, 6.13.2017, Mikko Pentinsaari, Barcode of Life DNA voucher specimen, Sample ID: BIOUG34206-H01, Process ID: MPCAN465-17. ***Paratypes*** (3 CBG, 4 CNC): **Canada**, labelled as the holotype except: Sample ID: BIOUG34206-H02, Process ID: MPCAN466-17 (1 male, CBG); Sample ID: BIOUG34206-G12, Process ID: MPCAN464-17 (1 male, CBG); Sample ID: BIOUG34206-G11, Process ID: MPCAN463-17 (1 female, CBG). **United States: North Carolina**: Haywood Co., 3 mi N Dellwood, 19.VIII.1972, A. Smetana (3, CNC); **Maryland**: Patuxent Wildl. Res. Ctr., 5 km E Montpelier, treading pond vegetation, 16.VI.1982, Bousquet & Davies (1, CNC).

##### Etymology.

The species epithet refers to the longitudinal impression on the pronotum, most strongly developed in males.

##### Diagnosis.

*Gnypetaimpressicollis* can be easily distinguished from all Nearctic species of the genus (except eastern *G.baltifera* (LeConte)) by the hexagonal pronotum with a longitudinal impression in the basal half (females) to nearly entire pronotal length (males). Males also have an impression on the vertex of the head. We have examined the female type of *G.baltifera* and it is externally similar but differs by the shorter, less angulate hexagonal pronotum, reddish and longer elytra and spermatheca with an elongate stem (C-shaped in *G.impressicollis*).

##### Description.

**Figure 13.**
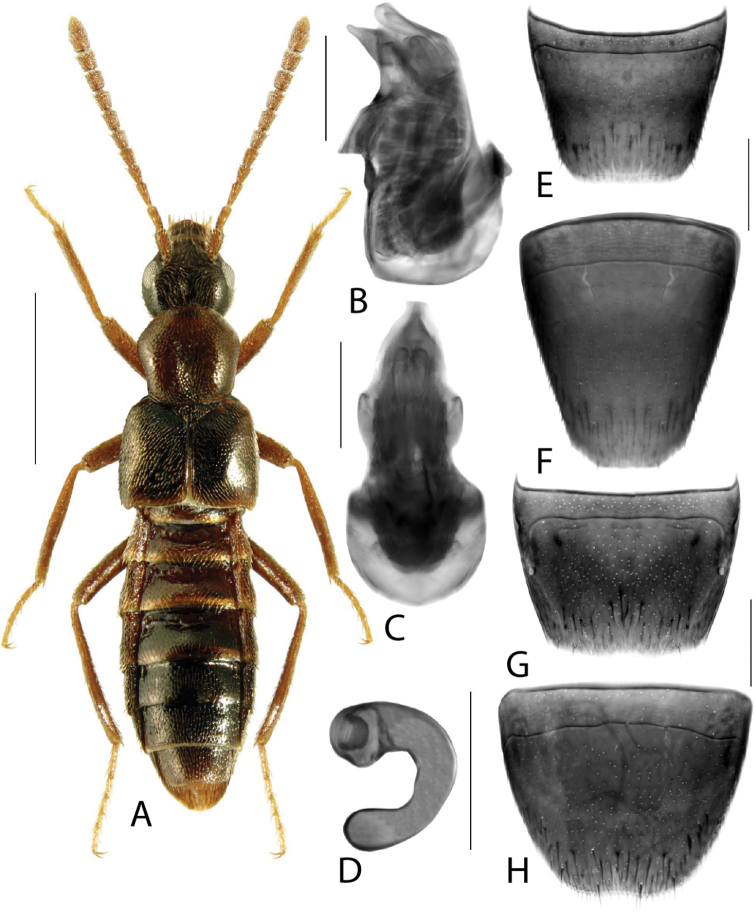
*Gnypetaimpressicollis* Klimaszewski, Brunke & Pentinsaari, sp. nov. **A** habitus **B** median lobe of aedeagus in lateral view **C** median lobe of aedeagus in dorsal view **D** spermatheca **E** male tergite VIII **F** male sternite VIII **G** female tergite VIII **H** female sternite VIII. Scale bars: 1 mm (**A**); 0.2 mm (**B–H**).

Body length 3.2–3.4 mm; colour dark brown, elytra brown with irregular rust-brown patches, first two or three basal tergites rust-brown with posterior edge yellow, apex of abdomen rust-brown, legs and antennae rust-brown; integument highly glossy (Fig. 13A); pubescence yellowish grey, moderately long and moderately sparse; all antennomeres distinctly elongate; head round with short neck (visible only when head is distended from thorax), vertex in males with broad central impression, vertex of females with much smaller and narrower median impression, maximum width of head slightly less than maximum width of pronotum; pronotum hexagonal in shape, ca. as long as head, with a longitudinal impression in the basal half (females) to nearly entire pronotal length (males), pubescence on disc directed anteriad along midline and obliquely laterad elsewhere; elytra wider than either head or pronotum, at suture shorter than pronotum along midline, pubescence directed obliquely posteriad forming wavy pattern medially on each side; abdomen arcuate laterally, broadest in apical third, at base distinctly narrower than elytra; legs very long, hind tarsus with basal tarsomere ca. as long as the two following ones combined. MALE. Tergite VIII with apical margin truncate medially and arcuate laterally (Fig. 13E); sternite VIII elongate, narrowed apically, apex truncate medially and oblique laterally (Fig. 13F); median lobe of aedeagus in lateral view with tubus very short, triangular and gradually tapering to narrowly rounded apex, ventral margin broadly curved ventrad in apical half (Fig. 13B); in dorsal view bulbus moderately large and tubus swelled basally and triangular apically (Fig. 13C); internal sac with complex membranous structures (Fig. 13B,C). FEMALE. Tergite VIII broadly arcuate apically (Fig. 13G); sternite VIII rounded apically with very shallow median emargination (Fig. 13H); spermatheca C-shaped, capsule subspherical with broad apical invagination, stem tubular and C-shaped (Fig. 13D).

##### Distribution.

**Origin**: Nearctic. **Canada**: ON. **United States**: MD, NC. *Gnypetaimpressicollis* is probably broadly distributed in eastern North America.

##### Bionomics.

Specimens were collected by sifting leaf litter along a lake margin and by treading pond vegetation.

##### Comments.

It was challenging to place this species in either *Gnypeta* or *Ischnopoda* Stephens based on the concepts of Paśnik (2010). The extremely long legs, pronotal shape, C-shaped spermatheca and superficial punctation of the pronotum and abdomen are consistent with at least some Neotropical members of *Ischnopoda* but the ligula of *G.impressicollis* is divided to the base, which is considered to be a feature of *Gnypeta* (Pašnik 2010). The C-shaped spermatheca of *Gnypetaimpressicollis* also bears some similarity to the *G.crebrepunctata* group of Klimaszewski et al. (2008) but it is rather different in external morphology. We place this species in *Gnypeta* pending future systematic research.

### Tribe Hypocyphtini Laporte, 1835

#### Oligota parva

Taxon classificationAnimaliaColeopteraStaphylinidae

Kraatz, 1862

17544666-193D-5AF3-AE5F-B1095FE817A0

BOLD:AAP9955

Fig. 14A–G

##### Material

**(DNA barcoded specimens). Germany**: Bornheim-Hemmerich, Ortslage, 50.7596, 6.93151, 30.VII.2010, F. Koehler (2, ZSM); Bornheim-Hemmerich, Ortslage, 50.7596, 6.93151, 25.VIII.2013, GBOL-Team ZFMK (1, ZFMK); Wutha-Farnroda, Wartburgkreis, 50.947, 10.4214, 25.VIII.2012, GBOL-Team ZFMK (2, ZFMK). **Canada: Ontario**: Kawartha Lakes, 44.296, -78.452, farm, malaise trap, 24.VII.2015, B. McClenaghan (1, CBG); same except 19.VII.2016 (1, CBG); Guelph, Arboretum, Urban Organic Farm, 43.5381, -80.222, compost heaps and mouldy hay pile, 17.IX.2017, M. Pentinsaari (4, CBG).

##### Distribution.

**Origin**: West Palaearctic (adventive in North America). **Canada**: NB, ON [new record], PE. **United States**: CA, MA, MO, NV, TX.

##### Bionomics.

This species is generally found in anthropogenic habitats, including compost, dung, and old hay and grass (Klimaszewski et al. 2018). In Canada, it has been collected in compost and in ocean coastline drift at the top of the littoral zone (Klimaszewski et al. 2018).

##### Comments.

*Oligotaparva* is a cosmopolitan species that is adventive in Canada. Here we newly report it from Ontario.

**Figure 14.**
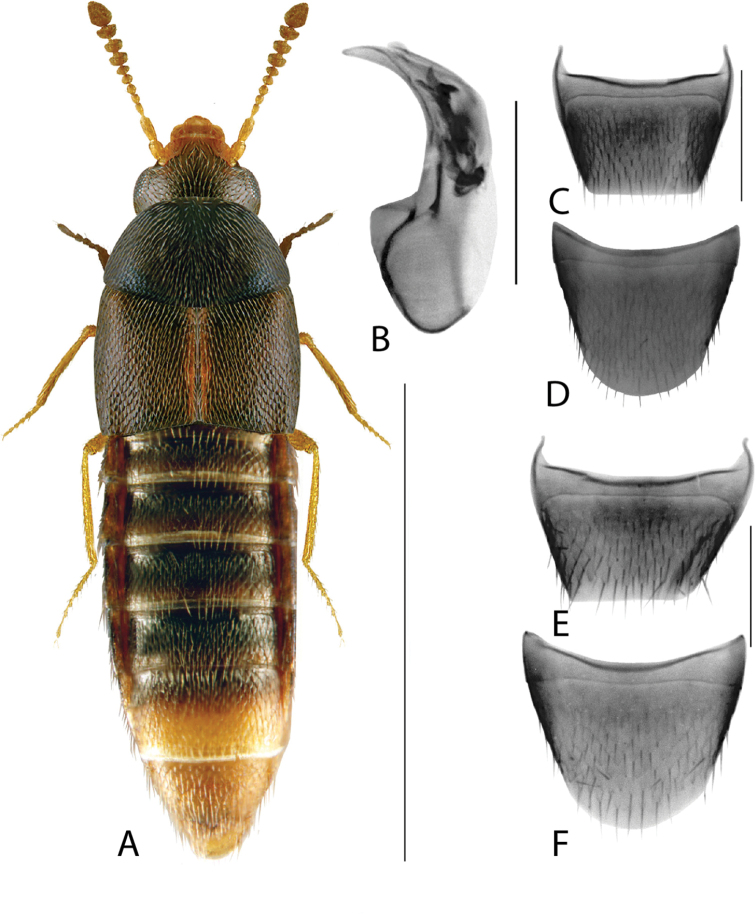
*Oligotaparva* Kraatz **A** habitus **B** median lobe of aedeagus in lateral view **C** male tergite VIII **D** male sternite VIII **E** female tergite VIII **F** female sternite VIII. Scale bars: 1 mm (**A**); 0.2 mm (**B–F**). Illustrations after Webster et al. (2016).

#### Oligota pumilio

Taxon classificationAnimaliaColeopteraStaphylinidae

Kiesenwetter, 1858

F4C78CF8-59A3-5149-AABF-7C1B68144429

BOLD:AAN4271

Fig. 15A–C

##### Material

**(DNA barcoded specimens). Belgium**: Blanden, BR Meerdaalboos, 50.7976, 4.71622, 8.V.2010, F. Koehler (1, ZSM). **Germany**: Neuburg, Altrheine, 48.9943, 8.24412, 29.IX.2011, F. Koehler (2, ZSM); Erftstadt-Bliesheim, NWZ Altwald Ville, 50.792, 6.844, 4.X.2010, F. Koehler (1, ZSM); Jockgrim, Sandmagerrasen, 49.0805, 8.26568, 14.XI.2010, F. Koehler (1, ZSM); Ochtendung, Michelsberg, 50.3631, 7.3889, 17.III.2012, F. Koehler (1, ZSM); Edenkoben-Rhodt, Villa Ludwigshoehe, 49.2767, 8.08991, 20.V.2012, F. Koehler (1, ZSM); Bad Muenster-Traisen, Rotenfels, 49.822, 7.832, 20.V.2012, F. Koehler & J. Koehler (1, ZSM); Osterholz bei Blankenburg, 51.9519, 11.0526, 18.III.2015, GBOL-Team ZFMK (2, ZFMK). **Canada: Alberta**: Waterton Lakes National Park, Red Rock Parkway, 49.088, -113.883, Moraine grassland, intercept trap, 1328 m, 11.VIII.2012, BIOBus 2012 (1, CBG). **United States: Montana**: Missoula County, Florence, MPG Ranch, 46.702, -114.034, grassland, pitfall trap, 05.VI.2019, M. Seidensticker (1, CBG).

##### Distribution.

**Origin**: West Palaearctic (adventive in North America). **Canada**: AB [new record]. **United States** (all except MT need verification): DC, IL, OH, MT [new record].

##### Diagnosis.

**Figure 15.**
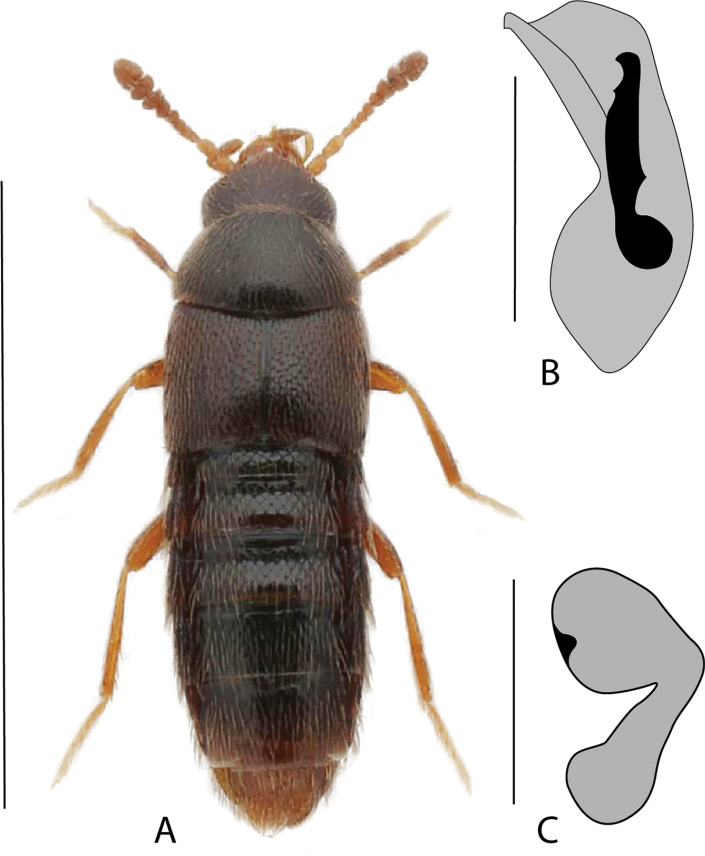
*Oligotapumilio* Kiesenwetter **A** habitus (image by A. Bogri – www.BilleBank.dk) **B** median lobe of aedeagus in lateral view (drawn from Kapp 2019) **C** spermatheca (drawn from Kapp 2019). Scale bars: 1 mm (**A**); 0.2 mm (**B, C**).

Among Canadian species of *Oligota*, *O.pumilio* is extremely similar to *O.pusillima* in the narrow, parallel body (Fig. 15A) and in male and female genitalia (Klimaszewski et al. 2018). However, it can be distinguished by the more abruptly truncate apex of the median lobe in lateral view (Fig. 15B), differently shaped sclerites of the internal sac (Fig. 15B), medially projected apex of male sternite VIII, and the transverse capsule of the spermatheca (Fig. 15C) (Kapp 2019).

##### Bionomics.

This species occurs in a wide variety of habitats across a broad elevational range, including hollow trees, plant debris, old hay in cattle barns, moldy substrates and in mushrooms (Kapp 2019). The barcoded Nearctic specimens were collected from grassland habitats by an intercept trap (Alberta) and a pitfall trap (Montana).

##### Comments.

*Oligotapumilio* is a West Palaearctic species that is adventive in Canada. Although it has been previously reported from the United States (OH, IL, DC) (Newton 2019), these records need confirmation as they are in the east, some distance away from the present records. This species’ presence in North America is thus verified here for the first time, from both Canada (AB) and United States (MT). It has also been reported as adventive from Argentina, Chile, and New Zealand (Newton 2019). The barcoded specimens of *O.pumilio* from Canada and the United States share the same DNA barcode haplotype, which is also shared by some of the specimens from Germany.

#### Oligota pusillima

Taxon classificationAnimaliaColeopteraStaphylinidae

(Gravenhorst, 1806)

EC04853E-57A2-5C81-B6BC-92C243700B56

BOLD:ABW7320

Fig. 16A–G

##### Material

**(DNA barcoded specimens). Finland**: Oba: Oulu, Hietasaari, 65.0225, 25.4247, 22.IV.2011, M. Pentinsaari (1, ZMUO); **Germany**: Edenkoben-Rhodt, Villa Ludwigshoehe, 49.277, 8.09, 20.V.2012, F. Koehler (2, ZSM); Edenkoben-Rhodt, Villa Ludwigshoehe, 49.277, 8.09, 23.VI.2012, F. Koehler (2, ZSM); Zweibruecken-Mauschbach, Monbijou-Wald, 49.2038, 7.39891, 16.X.2011, F. Koehler & W. Koehler (1, ZSM). **Canada: Alberta**: Two Hills, Two Hills School EQP-CLL-553, 53.7104N, 111.7437W, 613 m, Malaise trap, 21.IX.–2.X.2015, K. Warawa (2, CBG).

##### Additional non-barcoded material.

**Ontario**: Ottawa, Ottawa River, Deschênes Lookout, Berlese flood debris, 1.V.1985, A. Davies (1, CNC).

##### Distribution.

**Origin**: West Palaearctic (adventive in North America). **Canada**: AB [new record], NB, ON [new record]. **United States**: MA, NY.

##### Bionomics.

This species occurs in a variety of moist to dry, decaying organic matter including rotting hay, compost, hollow trees, and ant nests (Kapp 2019). Canadian specimens were collected in compost (Webster et al. 2016), and in malaise traps and flood debris in a suburban setting (present study).

##### Comments.

*Oligotapusillima* is a Palaearctic species that has been introduced to North America, South America, Australia, Africa, and southeast Asia (Kapp 2019, Newton 2019). It is here reported from Ontario and Alberta for the first time, the latter representing the westernmost record in North America. Specimens from Alberta were sequenced and their barcodes match those of Palaearctic specimens with no divergence. These specimens were also morphologically consistent with *O.pusillima*.

**Figure 16.**
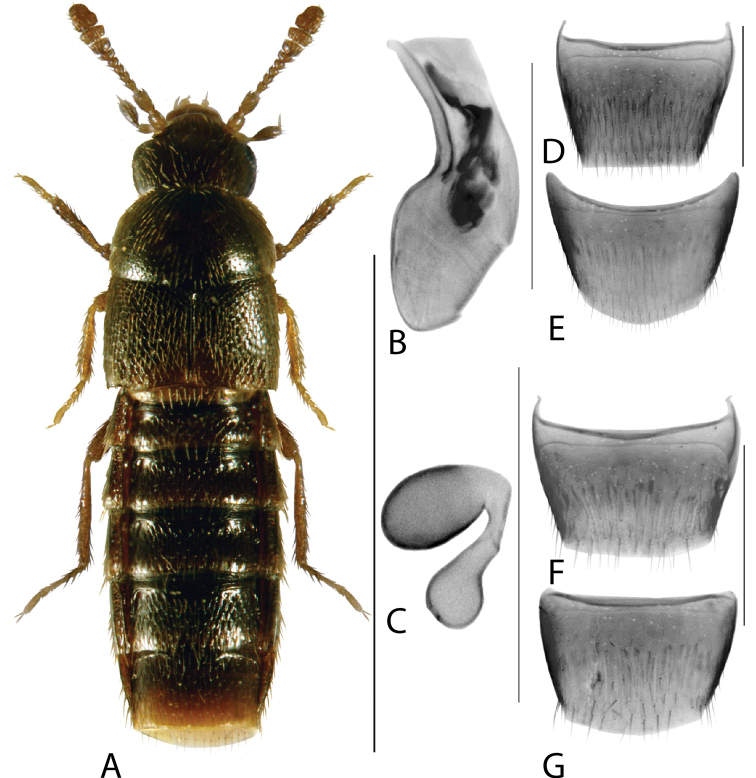
*Oligotapusillima* (Gravenhorst) **A** habitus **B** median lobe of aedeagus in lateral view **C** spermatheca **D** male tergite VIII **E** male sternite VIII **F** female tergite VIII **G** female sternite VIII. Scale bars: 1 mm (**A**); 0.2 mm (**B–G**).Illustrations after Webster et al. (2016).

### Homalotini Heer, 1839

#### Anomognathus athabascensis

Taxon classificationAnimaliaColeopteraStaphylinidae

Klimaszewski, Hammond & Langor, 2016

7735FC0D-73F4-51C0-9AA5-7B2FBE84AFE7

Fig. 17A–G

##### Material.

Non-sequenced specimens. **Canada: Manitoba**: Winnipeg, under bark of rotten ‘N. aceroides’ [= *Acernegundo*], 27.VIII.1918, J.B. Wallis (2, CNC).

##### Distribution.

**Origin**: Nearctic. **Canada**: AB, MB [new record].

##### Bionomics.

The MB specimens were collected under bark, confirming that this species lives in a way similar to other members of the genus.

##### Comments.

*Anomognathusathabascensis*, recently described from Alberta (Klimaszewski et al. 2016b), is newly reported from Manitoba. This native Nearctic species is likely transcontinental but rarely reported due to its small size and elusive habits.

**Figure 17.**
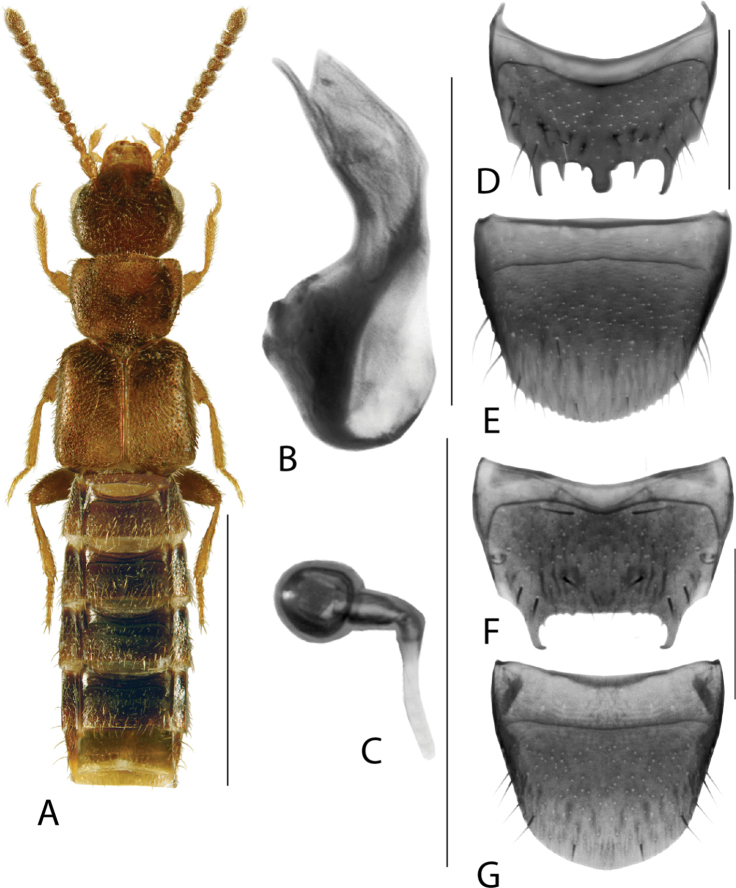
*Anomognathusathabascensis* Klimaszewski, Hammond & Langor **A** habitus **B** median lobe of aedeagus in lateral view **C** spermatheca **D** male tergite VIII **E** male sternite VIII **F** female tergite VIII **G** female sternite VIII. Scale bars: 1 mm (**A**); 0.2 mm (**B–G**). Illustrations after Klimaszewski et al. (2016b).

#### Anomognathus cuspidatus

Taxon classificationAnimaliaColeopteraStaphylinidae

(Erichson, 1839)

A7476206-A27C-52E7-8349-BDD471A204A8

BOLD:AAO0339

**Figure 18.**
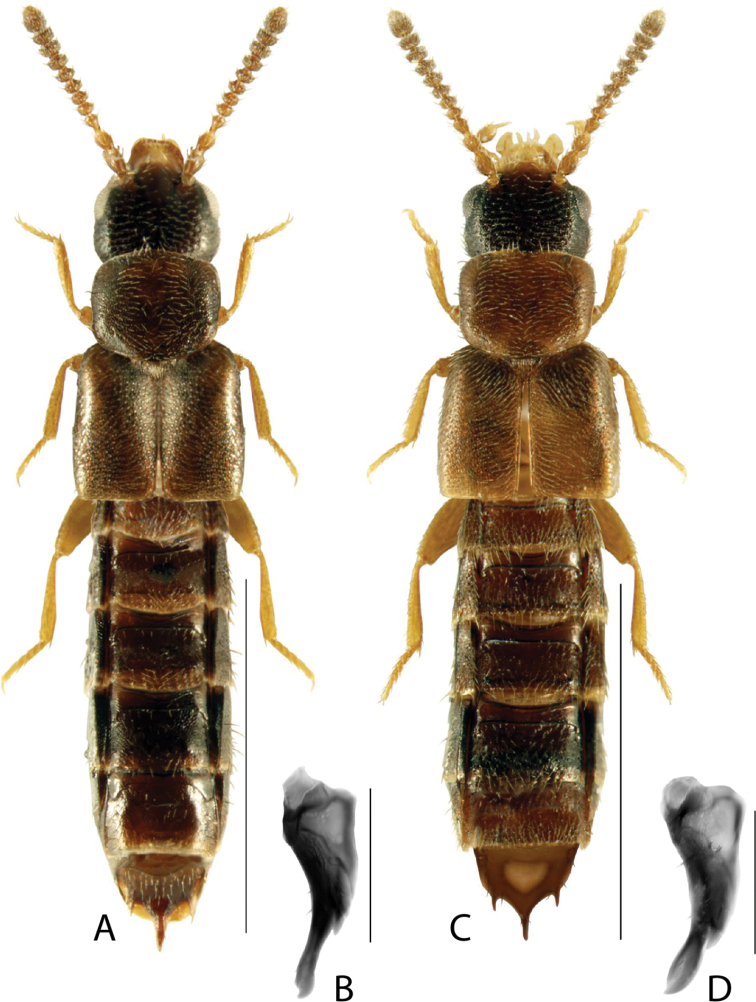
**A, B***Anomognathuscuspidatus* (Erichson), and **C, D***Anomognathus* sp., putative undescribed species (Europe) **A, C** habitus and **B, D** male tergite VIII in lateral view. Scale bars: 1 mm (**A, C**); 0.2 mm (**B, D**).

**Figure 19.**
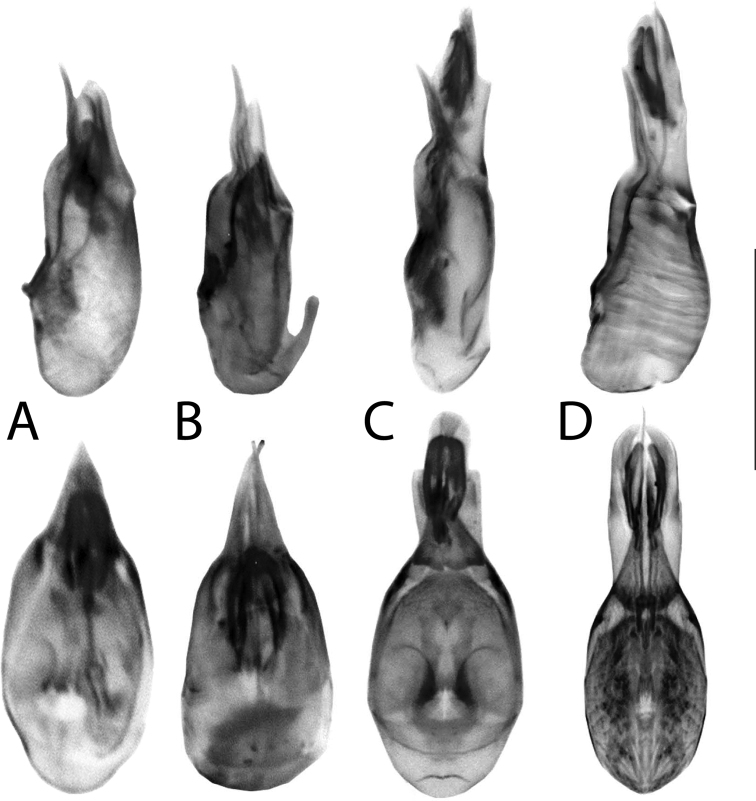
Aedeagi of **A–C***Anomognathuscuspidatus* (Erichson) and **D** potential undescribed species, in lateral view (top row) and dorsal view (bottom row) **A** sequenced non-type (Ontario, Canada) **B** lectotype of *A.cuspidatus* (‘Europe’) **C, D** sequenced non-types (Finland). Scale bar: 0.2 mm.

**Figure 20.**
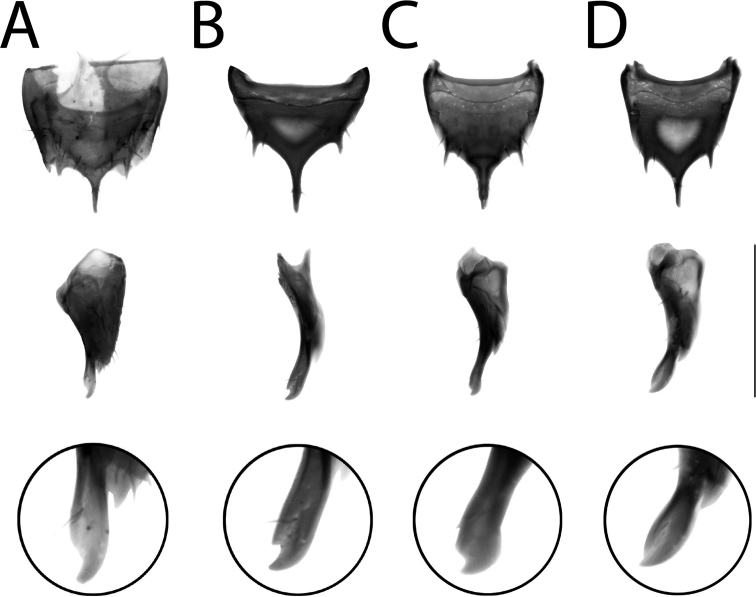
Male tergite VIII of **A–C***Anomognathuscuspidatus* (Erichson) and **D** potential undescribed species, in dorsal (top row) and lateral (middle and bottom rows) **A** lectotype of *A.cuspidatus* (‘Europe’) **B** holotype of *A.americanus* (Casey) (= *A.cuspidatus*) **C, D** sequenced, non-types (Finland). Scale bar: 0.2 mm.

Figs 18A–D , 19A–D , 20A–D.

Homalota cuspidata Erichson, 1839
Thectura americana Casey, 1893, syn. nov.
Anomognathus americanus : Seevers (1978) (as valid species)

##### Type material.

*Homolotacuspidata* Erichson, 1839. ***Lectotype***, male, here designated (ZMHB): *cuspidata* Er: [handwritten label] / 5387 [typed label] / Hist.-Coll. (Coleoptera), Nr. 5387, *Homalotacuspidata*, Erichs., Europa, Zool. Mus. Berlin [typed white label] / Lectotype *Homalotacuspidata* des. J. Klimaszewski 2019 [white printed label]. ***Paralectotypes*** (3, ZMHB, without original labels): Hist.-Coll., (Coleoptera), Nr. 5387, *Homolotacuspidata* Erichs., Europa, Zool. Mus. Berlin; Syntype *Homolotacuspidata* Erichson, 1837, labelled by MNHUB 2010; Paralectotype *Homalotacuspidata* des. J. Klimaszewski 2019 [white printed label] [1 female, spermatheca and terminalia dissected in Canada balsam on microslide attached to specimen]; same labels except: SYNTYPUS, *Homalotacuspidata* Erichson, 1837 [typed red label, added by MNHUB 2010] [1 female, spermatheca and terminalia dissected in Canada balsam on microslide attached to specimen]; same labels as before [1 damaged specimen, sex undetermined].

Males and females of the syntype series were morphologically consistent with the specimens forming molecular cluster BOLD:AAO0339, including those sequenced from Ontario, Canada. As the most obvious difference between *A.cuspidatus* and the potential new Central European species (see Diagnosis) was the shape of the median process on male tergite VIII (in lateral view) (Fig. 18B, D), a male syntype (see above) was designated as the lectotype of this species to fix its identity. Morphology of the aedeagus itself was difficult to study due to its small size and obvious differences between molecular clusters (see below) were not observed (Fig. 19).

*Thecturaamericana* Casey, 1893, syn. nov. Holotype (male) (NMNH): NY/ TYPE USNM 39614/ *Thecturaamericana* Casey (handwritten by Casey).

Casey (1893) gave numerous characters to distinguish *A.americanus* from *A.cuspidatus* but all of these were observed to be highly variable within populations in the material studied, including the shape of apical antennomeres, shape of the pronotum, position of the abdominal tubercles in the male, and the type of dorsal expansion of the median process of male tergite VIII. We could not find the depression at the base of tergite VIII on the holotype of *A.americanus* mentioned by Casey (1893). Although the aedeagus of the holotype was not studied (not extracted from partly damaged and fragile pygidium), male tergite VIII was intact and its median process in lateral view bears an apical hook, matching the present concept for *A.cuspidatus* (Fig. 20). Therefore, in corroboration with Fenyes (1918), we treat *A.americanus* as a synonym of *A.cuspidatus*.

##### Non-type material

**(sequenced specimens indicated in square brackets). Canada: Alberta**: Peace River, 25 km NW Peace River, 17–23.VIII.1993, J. Hammond (2, CNC); **Ontario**: Wellington County, Guelph, Eramosa River Trail, 43.539, -80.236, deciduous forest, 14.IV.2017, M. Pentinsaari (4, CBG [4 barcoded]).

A photo record of this species from Ontario is available on bugguide.net (/view/1816108): Toronto, 19.V.2020, under bark, O. Strickland.

**Belgium**: Sint-Genesius-Rode, BR Zonienwoud, 50.7505, 4.423, 28.IV.2010, F. Koehler (1, ZSM [1 sequenced]). **Czech Republic (all CNC)**: Bohemia, Poděbrady 50 km, Smetana, 1959, car net trap (1); Bohemia, Chvojno, Smetana (1); Moravia, Drnholec, Smetana (1). **Denmark (all NHMD)**: Staksrode, EJ, 24.IX.1983 (1); Æbelø F, 18.V.1997 (1); Faested Mose, SJ, 12.IV.1986 (1); Dyrehaven, 14.4.1934 (1); same except 21.3.1923 (1); same except 21.10.1932 (1); 30.4.1922 (1); same except 19.5.1911 (1); Lyng Huse, 29.3.1997 (1).

**Germany**: Nationalpark Mueritz, Babke-Zartwitz-Speck-Schwarzenhof, 53.4125, 12.8463, car net, 20.VI.2015, GBOL-Team ZFMK (2, ZFMK [2 sequenced]); Hoenningen bis Insul, Ahrtal, 50.45, 6.942, 24.IV.2010, F. Koehler (1, ZSM [1 sequenced]); Oberheimbach, Franzosenkopf, 50.004, 7.805, 27.V.2012, W. Koehler (1, ZSM [1 sequenced]). **Finland (all ZMUO)**: N: Espoo, Saunalahti, 60.1643, 24.6263, 17.IX.2012, fungusy aspen logs, E. Helve (1) [barcoded]; Al: Bjoerkoe, 59.9769, 20.1879, sifting, 24.IX.2014, M. Pentinsaari (1) [barcoded]; Ta: Lammi, R. Linnavuori leg. (1); Ab: Naantali, R. Linnavuori leg. (1); Kb: Lieksa, R. Linnavuori (1); Rynmattyla, 24.VI.1945, Karvonen (2); same except 14.VIII.1945 (2). **Slovakia (all CNC)**: Cenkov, Smetana, 1963 (11); Nová Sedlica, Smetana, 1961 (2); Ruská Poruba, Smetana, 1956 (2). **United Kingdom (all CNC)**: Essex (6).

Putative undescribed *Anomognathus* (corresponding to BIN BOLD:ACA9191):

**Finland (all ZMUO)**: N: Espoo, E. Helve, 1978 (1); same except 1976 (1); same except 1977 (1); same except 1979 (1); same except 1981 (1); same except 1982 (1); Ks: Taivalkoski, 728.53 Window trap, 2003, E. Hurme (2); same except *Polyporus* trap (1); Kb, Kitee, 23.05.2016, M. Pentinsaari leg., [1 sequenced]; Obb: Rovaniemi, Rovajärvi, 16.6–8.7.2010, M. Pentinsaari and E. Kuusela [1 sequenced]. **Germany**: Schleiden-Wolfgarten, Dachsloecher, 50.6098, 6.42237, 26.VII.2012, F. Koehler (1, ZSM [1 sequenced]).

##### Diagnosis.

*Anomognathuscuspidatus* is distinctive for its trident-shaped apex of male and female tergite VIII (Fig. 20A–C) and can be distinguished from all described species by this feature alone. However, in the course of this study, specimens representing a remarkably divergent barcode cluster (BOLD:ACA9191; 9.63% uncorrected p-distance to *A.cuspidatus*) were investigated and found to likely represent an undescribed species of *Anomognathus* in Europe (confirmed specimens from Finland and Germany). Although most morphological characters of *A.cuspidatus* and the putative new species are highly variable, including the median lobe of the aedeagus, males can be dependably separated based on the shape of their median process of tergite VIII in lateral view: *A.cuspidatus* bears a minute to distinct hook at the apex (Fig. 20A–C), while in the undescribed species, the median process converges evenly to a single point, creating an elongate, turnip-shape (Fig. 20D). The shape of tergite VIII in females was observed to be extremely variable and no features were deemed to be diagnostic. Externally, most specimens can be recognized as either species (especially males) by the relative proportions of the head versus the pronotum, with *A.cuspidatus* generally bearing a small pronotum, narrower than the head (Fig. 18A) and the undescribed species bearing a wider, longer pronotum, wider than the head (Fig. 18C). The limits of this taxon need further investigation and should include morphological study of a much wider range of sequenced material.

##### Distribution.

**Origin**: West Palaearctic (adventive in North America). **Canada**: AB, NB, ON. **United States**: NY.

##### Bionomics.

This species occurs under the bark of dead trees. One specimen (NB) was collected from a Lindgren funnel.

##### Comments.

*Anomognathuscuspidatus* is a widespread West Palaearctic species that is known from Europe, European Russia and Algeria (Newton 2019) and has been previously known in North America under the synonym *A.americanus*. The record from Beijing, China should be verified. The species has become introduced in North America (before 1893) and it is unclear whether the population in Alberta represents a separate introduction from Europe, a secondary introduction from eastern North America or a broad adventive distribution across Canada.

After the results of the present study, two species of *Anomognathus* are known to occur in North America: native *A.athabascensis* Klimaszewski, Hammond & Langor and the adventive *A.cuspidatus*. These are easily separated by the drastically different shapes of male and female tergites VIII (Figs 17D, 20A–C). Previously, only females of *A.cuspidatus* (as *A.americanus*) were available from Canada (Klimaszewski et al. 2016b; Webster et al. 2016). Here we demonstrate that all available Nearctic *Anomognathus* specimens with a trident-shaped tergite VIII correspond to Palaearctic *A.cuspidatus*.

#### Cyphea wallisi

Taxon classificationAnimaliaColeopteraStaphylinidae

Fenyes, 1921

0EBFFF0B-32F6-5C94-89D4-73616E174085

BOLD:ACK1459

Figs 21A–G , 22A–D

Cyphea wallisi Fenyes, 1921
Agaricomorpha vincenti Klimaszewski & Webster, 2016, syn. nov.
Agaricomorpha vincenti : Klimaszewski et al. 2018 (as synonym of C.curtula)

##### Type material.

*Cypheawallisi* Fenyes, 1921. ***Paratype***, male (MCZ). Winnipeg, Man. [handwritten label] / Wallis [handwritten label] / 25490. / Cyphea, Wallisi, Feny [handwritten label] / Type., 9989, 9983 [typed red label].

*Agaricomorphavincenti* Klimaszewski & Webster, 2016, syn. nov. ***Holotype***, male (LFC). Canada, New Brunswick, Carleton Co., Jackson Falls, “Bell Forest”, 46.2200°N, 67.7231°W, 7–21.VI.2012, C. Alderson & V. Webster, coll. [white typed label] / Rich Appalachian hardwood forest, Lindgren funnel trap in canopy of *Fagusgrandifolia* [white typed label] / Holotype *Agaricomorphavincenti* Klimaszewski & Webster, 2016 [red typed label] / *Cypheacurtula* (Erichson) det. Klimaszewski 2017 [white typed label] / *Cypheawallisi* Fenyes det. A. Brunke 2020.

**Figure 21.**
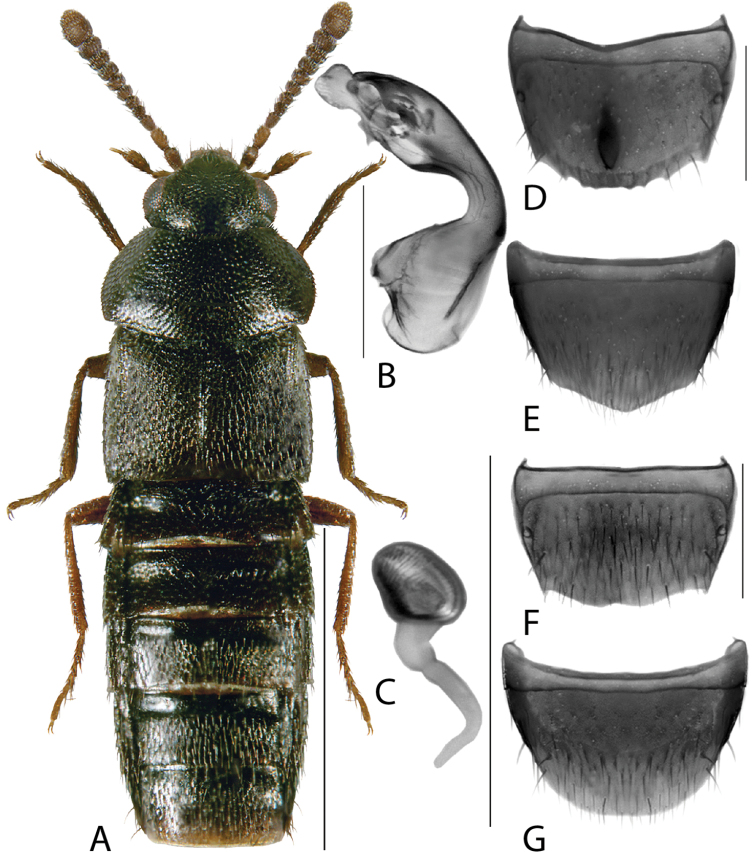
*Cypheawallisi* Fenyes **A** habitus **B** median lobe of aedeagus in lateral view **C** spermatheca **D** male tergite VIII **E** male sternite VIII **F** female tergite VIII **G** female sternite VIII. Scale bars: 1 mm (**A**); 0.2 mm (**B–G**). Illustrations after Klimaszewski et al. (2018), used with permission.

**Figure 22.**
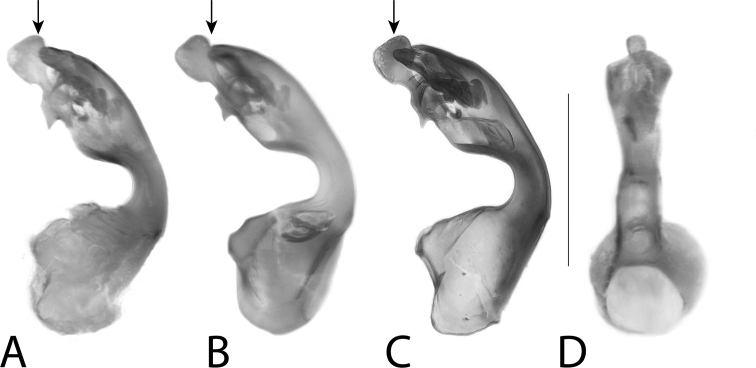
Aedeagi of *Cypheawallisi* Fenyes (**A, B, D**) and *C.curtula* (Erichson) (**C**), in lateral (**A–C**) and dorsal (**D**) view. Paratype of *C.wallisi* (**A, D**); holotype of *Agaricomorphavincenti* Klimaszewski and Webster (= *C.wallisi*) (**B**); non-type, *C.curtula* (image by V. Assing) (**C**). Scale bar: 0.2 mm.

The aedeagi of the male paratype (holotype in collection of the California Academy of Sciences) of *C.wallisi* and holotype of *A.vincenti* are identical and both differ from that of Palaearctic *Cypheacurtula* (image by V. Assing) by the broader distal lobe in lateral view, which only slightly extends beyond the distal plate (Fig. 22A–C). Therefore, we transfer *Agaricomorphavincenti* from synonymy with *Cypheacurtula* to synonymy with *Cypheawallisi*.

##### Other material

**(DNA barcoded specimens). Canada: Ontario**: Rouge National Urban Park, Toronto Zoo, 43.8223, -79.1897, forest, malaise trap, 25.VI.2013, L. Attard and K. Greenham (2, CBG); Hartington, Eel Lake Cottage, Lindgren funnel trap, 44.5628, -76.553, 25.VII.2017, G. Blagoev (1, CBG); **Nova Scotia**: Clyburn Valley Road, near golf course, Cape Breton National Highlands NP, forest, Malaise trap, 46.6553, -60.4285, 28.VI.2013, CBH staff (1, CBG).

##### Non-barcoded specimens.

**Canada: Quebec**: Mont St. Bruno Prov Park, 45.541, -73.319, Lindgren funnel, trap 5, tree 2, beech-maple canopy, 21.VII-3.VIII.2005 (1, CNC); Oka Prov Park, Lindgren funnel, trap 3, tree 1, beech-maple canopy, 27.VII.30.VIII.2005 (1, CNC).

##### Distribution.

**Origin**: Nearctic. **Canada**: AB, MB, NB, NS [new record], ON [new record], QC.

##### Bionomics.

Specimens have been collected in Malaise traps, window traps and Lindgren funnels placed in forests. Both the closely related West Palaearctic *C.curtula* and *C.latiuscula* Sjöberg have been consistently collected under bark, where they occur in the larval burrows of various longhorn beetles (Cerambycidae), bark beetles (Curculionidae: Scolytinae) and the carpenter moth (*Cossus* L.) (Palm 1968).

##### Comments.

*Cypheawallisi* is a broadly distributed native Nearctic species, reported from AB east to NS. Here we treat Nearctic records of *Cyphea* as *C.wallisi* (previously treated as Palaearctic *C.curtula*, e.g., Klimaszewski et al. 2018) and newly report the genus from ON and NS. *Cypheawallisi* is probably far more broadly distributed in North America than currently known and has been underreported due to its small size.

Sequenced Nearctic specimens of *Cyphea* from ON and NS formed a barcode cluster that was nearly 5% divergent from those of Palaearctic specimens of *C.curtula* (BOLD:AAO1175, one published sequence record from Belgium and three unpublished records from the Netherlands). Northern European *C.latiuscula*, the only other species of the genus, has a broader body outline, different male genitalia and is quite differently colored (bicolored pronotum and pale elytra). No barcode sequence data are currently available for *C.latiuscula*. Based on the study of one paratype of *C.wallisi*, described from Manitoba and not reported since, it was discovered that Nearctic specimens of *Cyphea* correspond to this species and differ from Palaearctic *C.curtula* by the broader distal lobe of the median lobe of the aedeagus in lateral view, which only slightly extends beyond the distal plate (Fig. 22A–C). The shape of the median lobe of the aedeagus in dorsal view may also be diagnostic (Fig. 22D) but a preparation in this view was unavailable for *C.curtula*. The illustration in Palm (1968) of the aedeagus of *C.curtula* in dorsal view appears to be less angulate than that of *C.wallisi* but this needs verification. Based on these differences in male genitalia (Fig. 22A–C) and the COI barcodes, *Cypheawallisi* is morphologically and genetically distinct from Palaearctic *C.curtula*, and the latter species does not occur in North America as far as known. Both of these species have a median tubercle on male tergite VII, mentioned earlier by Fenyes (1921) but this structure was omitted from the illustrations in Klimaszewski et al. (2018), though it was present in the original description of synonym *Agaricomorphavincenti* (Webster et al. 2016). Previous differences between the two species given by Klimaszewski et al. (2018) (e.g., projecting pronotal angles, lighter/darker body) proved to be highly variable.

#### Gyrophaena affinis

Taxon classificationAnimaliaColeopteraStaphylinidae

Mannerheim, 1830

C933A12D-0B8F-51BE-8FAE-7A344E583D3C

BOLD:ACF7981 [Nearctic]; BOLD:ABW9049 and BOLD:AAO0291 [both Palaearctic]

Fig. 23A–G

##### Material

**(DNA barcoded specimens). Belgium**: Sint-Genesius-Rode, BR Zonienwoud, 50.7505, 4.423, 135 m, 16.VI.2010, F. Koehler (1, ZSM). **Finland**: Oba: Oulu, Linnanmaa, 65.0633, 25.4712, 7.VI.2011, M. Pentinsaari (1, ZMUO); Obb: Tornio, Kalkkimaa, 65.9014, 24.4711, 10.VII.2012, M. Pentinsaari (1, ZMUO); Al: Lemland, Herrövägen, 59.9796, 20.1954, car net, 5.VII.2012, M. Pentinsaari (1, ZMUO). **Germany**: Brohl-Luetzing, Brohltal, 50.4727, 7.31272, 22.V.2010, F. Koehler (1, ZSM), Riedlhuette, Diensthuettenstrasse, 48.937, 13.412, 09.VII.2011, F. Koehler & M. Koehler (1, ZSM), Waldhaeuser, Lusen- und Boehmstrasse, 48.93, 13.492, 09.VII.2011, F. Koehler & M. Koehler (1, ZSM). **Canada: Alberta**: Waterton Lakes National Park, Highway 6 pulloff, 49.065, -113.779, 1569 m, intercept trap, montane forest, 27.VI.2012, BIOBus 2012 (2, CBG).

##### Distribution.

**Origin.** Uncertain. **Canada**: AB [new record], BC, MB, NB, NF, NS, ON, QC, SK. **United States**: AZ, DC, IL, IN, IA, KY, MA, ME, MI, MN, MO, NC, NH, NJ, NM, NY, OH, PA, TN, WA, WI, WV.

##### Comments.

*Gyrophaenaaffinis* is newly reported from AB based on barcoded material.

**Figure 23.**
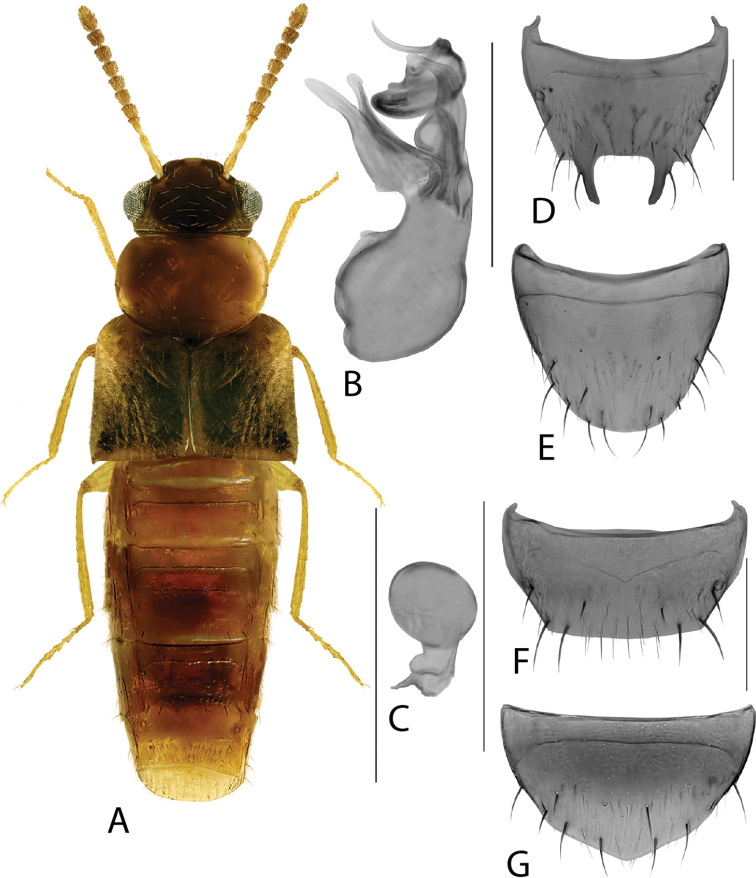
*Gyrophaenaaffinis* Mannerheim **A** habitus **B** median lobe of aedeagus in lateral view **C** spermatheca **D** male tergite VIII **E** male sternite VIII **F** female tergite VIII **G** female sternite VIII. Scale bars: 1 mm (**A**); 0.2 mm (**B–G**). Illustrations after Klimaszewski et al. (2018), used with permission.

Sequenced Nearctic specimens from ON, AB, NB, and QC form a distinct barcode cluster, separate from all sequenced Palearctic specimens and divergent by 4.65%. This pattern is inconsistent with a species that is adventive in North America and we remove *G.affinis* from the list of adventive species in Canada. In comparing images between those of Nearctic specimens (Fig. 23B) and those of Enushchenko and Semenov (2016) for Palaearctic specimens, there appear to be slight differences in the median lobe of the aedeagus in lateral view. In the Palaearctic illustration, the apex of the median lobe is more acute and its secondary lobe is evenly rounded at apex, while the Nearctic illustration shows a more rounded apex of the median lobe and knob-like apex of the secondary lobe (Fig. 23B). More research is needed to determine the status of the Nearctic and Palaearctic populations, though the level of genetic divergence between discrete Nearctic and Palaearctic populations suggests that two sister species are involved.

#### Gyrophaena gracilis

Taxon classificationAnimaliaColeopteraStaphylinidae

Seevers, 1951

A955BCE4-D571-59B3-A8DA-CCF2E57F884F

Fig. 24A–H

##### Material

**(non-sequenced material). Canada: Quebec**: Gatineau Park, wolf trail, near trail start, 45.541, -75.912, hardwood forest, *Polyporussquamosus* on large beech log, 8.VI.2019, A. Brunke & J. Smith (1, CNC).

##### Distribution.

**Origin.** Nearctic. **Canada**: NB, QC [new record]. **United States**: WI.

##### Bionomics.

Specimens have been collected from a partly dried *Pleurotus* mushroom, from within the pores of a *Trametes* polypore, and from the nest contents of a Barred owl (*Strixvaria* Barton) (Klimaszewski et al. 2018). The specimen from QC was collected from *Polyporussquamosus* on a beech tree.

##### Comments.

The new record from QC, near the ON border, bridges the wide gap between previous records in NB and WI.

**Figure 24.**
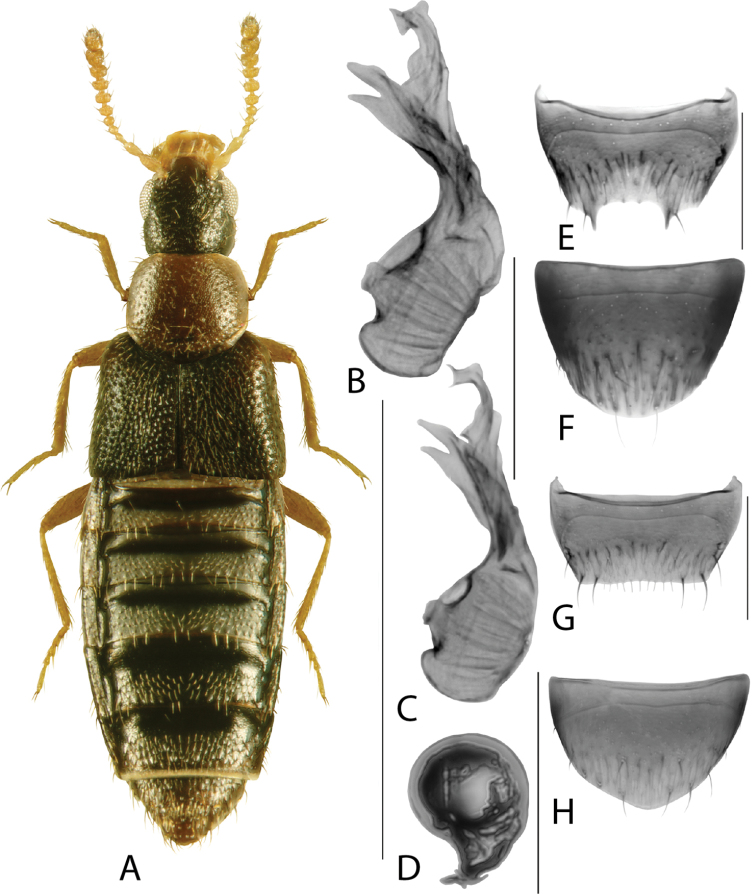
*Gyrophaenagracilis* Seevers **A** habitus **B, C** median lobe of aedeagus in lateral view **D** spermatheca **E** male tergite VIII **F** male sternite VIII **G** female tergite VIII **H** female sternite VIII. Scale bars: 1 mm (**A**); 0.2 mm (**B–H**). Illustrations after Klimaszewski et al. (2018), used with permission.

#### Gyrophaena simulans

Taxon classificationAnimaliaColeopteraStaphylinidae

Seevers, 1951

7FD8B947-6FC3-5A0E-BDDE-4943A6B64484

BOLD:ACY8004

Fig. 25A–G

##### Material

**(DNA barcoded specimens). Canada: Ontario**: Hartington, Eel Lake Cottage, 44.563, -76.549, deciduous forest, mushrooms, 4.X.2017, M. Pentinsaari (2, CBG).

##### Distribution.

**Origin.** Nearctic. **Canada**: ON [new record]. **United States**: IL, MD, PA.

##### Diagnosis.

**Figure 25.**
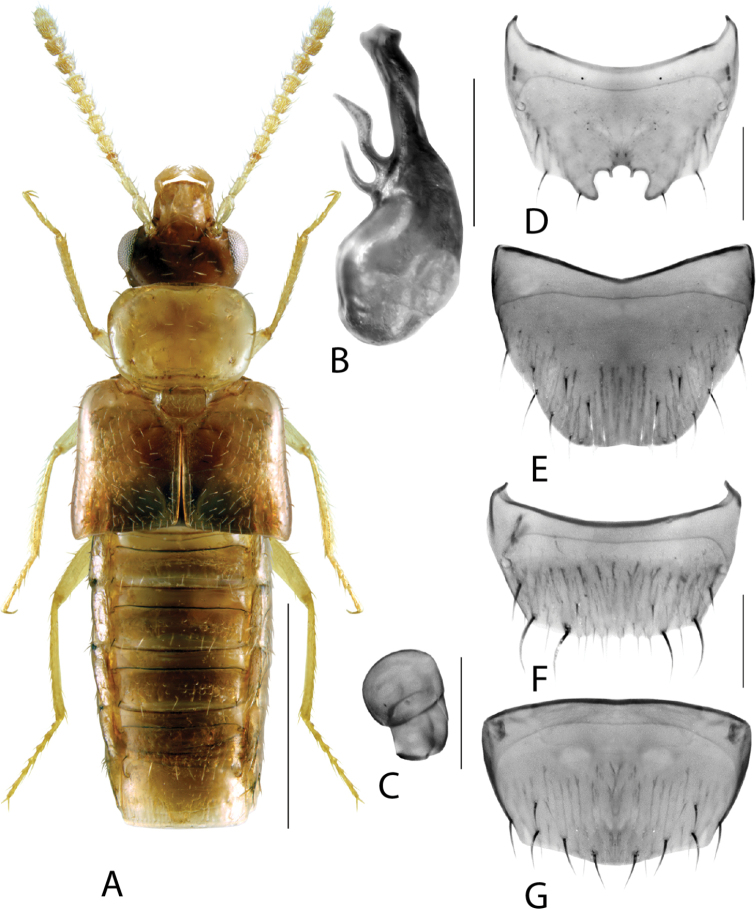
*Gyrophaenasimulans* Seevers **A** habitus **B** median lobe of aedeagus in lateral view **C** spermatheca **D** male tergite VIII **E** male sternite VIII **F** female tergite VIII **G** female sternite VIII. Scale bars: 1 mm (**A**); 0.2 mm (**B–G**).

*Gyrophaenasimulans* is extremely similar to *G.criddlei* and *G.pseudocriddlei* but has a slightly more transverse and flatter pronotum, with straighter apical and basal margins, and differently shaped upper process of the median lobe in lateral view (Fig. 25B): longer than that of *G.pseudocriddlei* but shorter and broader than that of *G.criddlei*. The emargination of male tergite VIII in *G.simulans* appears to be shallower and broader than that of *G.criddlei* but more specimens are needed to confirm this.

##### Bionomics.

The Canadian specimens were collected by sifting mushrooms in a deciduous forest. No detailed data on the host fungus were recorded.

##### Comments.

*Gyrophaenasimulans* is a native Nearctic species distributed in eastern North America and is newly reported from Canada. The barcode cluster BOLD:ACY8004 also contains specimens identified as related species *G.criddlei* (female) and *G.pseudocriddlei* but more research, with broader sampling of sequenced, identified males, is needed to determine whether these species share a BIN or these specimens are misidentified. As we were unable to verify the identifications at this time, these records are not published here.

#### Homalota plana

Taxon classificationAnimaliaColeopteraStaphylinidae

(Gyllenhal, 1810)

8C64540B-A2E8-5F40-A4E4-4F1EF11047F0

BOLD:ADH5714 [Nearctic]; BOLD:AAO0434 [Palaearctic]

Fig. 26A–G

##### Material

**(DNA barcoded specimens). Belgium**: Sint-Genesius-Rode, BR Zonienwoud, 50.7505, 4.423, 28.IV.2010, F. Koehler (1, ZSM). **Germany**: Arnsberg-Breitenbruch, NWZ Hellerberg, 51.446, 8.135, 30.V.2011, F. Koehler (2, ZSM); Heimbach-Blens, Linkheld, 50.648, 6.468, 29.VIII.2012, F. Koehler (2, ZSM); Erftstadt-Bliesheim, NWZ Altwald Ville, 50.7917, 6.84384, 03.VI.2011, F. Koehler (1, ZSM); westl. Klein-Quenstedt, 51.9239, 11.0478, 20.III.2015, GBOL-Team ZFMK (1, ZFMK). **Finland**: Al: Finström, Norrö, 60.2458, 19.822, 5.VII.2012, M. Pentinsaari (1, ZMUO); Ka: Joutseno, Kuurmanpohja, 61.071, 28.75, 3.VIII.2012, M. Pentinsaari (1, ZMUO). **Canada: Ontario**: Guelph, Eramosa River Trail, 43.539, -80.236, deciduous forest, 14.IV.2017, M. Pentinsaari (2, CBG).

##### Distribution.

**Origin.** Uncertain. **Canada**: AB, MB, NB, NF, NS, ON. **United States**: AZ, CA, CO, IA, ID, IN, MT, NY, OH, PA, TX.

##### Bionomics.

Specimens occur under bark of dead trees.

##### Comments.

Sequenced Nearctic specimens from ON form a distinct barcode cluster, separate from all sequenced Palearctic specimens and divergent by 7.58%. This pattern is inconsistent with a species adventive in North America and we remove *H.plana* from the list of adventive species in Canada. Preliminary comparisons between images of Palaearctic and Nearctic specimens revealed that there may be some slight differences in the shape of the spermatheca. More research is needed to determine the status of the Nearctic and Palaearctic populations, though the level of genetic divergence between discrete Nearctic and Palaearctic populations suggests that two sister species are involved.

**Figure 26.**
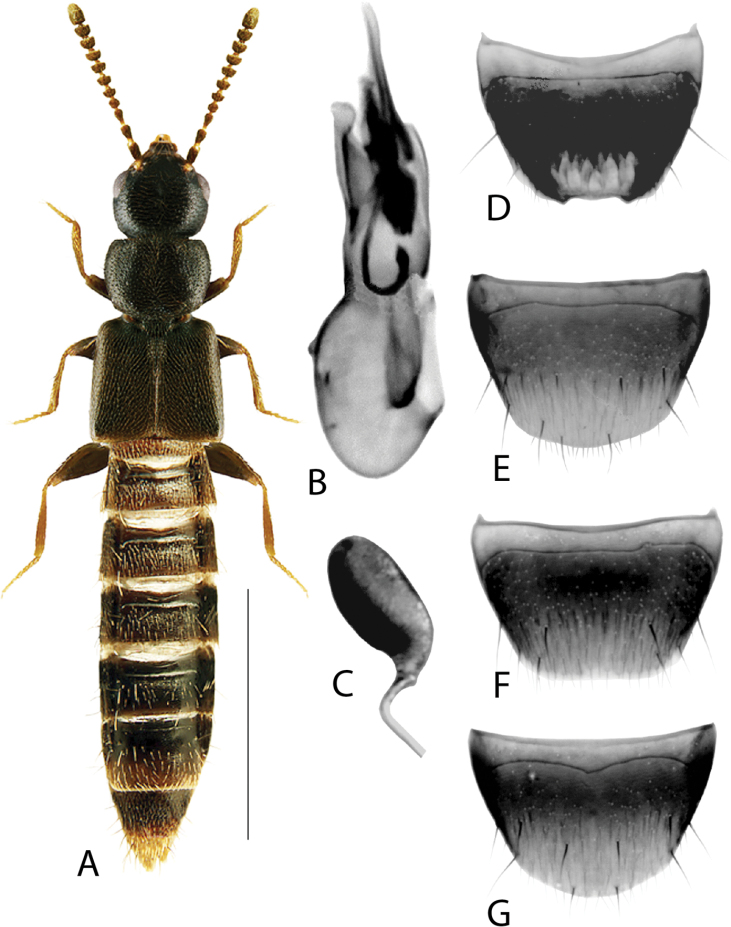
*Homalotaplana* (Gyllenhal) **A** habitus **B** median lobe of aedeagus in lateral view **C** spermatheca **D** male tergite VIII **E** male sternite VIII **F** female tergite VIII **G** female sternite VIII. Scale bars: 1 mm (**A**); 0.2 mm (**B–G**). Illustrations after Klimaszewski et al. (2018), used with permission.

#### Thecturota tenuissima

Taxon classificationAnimaliaColeopteraStaphylinidae

Casey, 1893

CB885C22-DF9B-587F-8888-136D80C98EA5

BOLD:AAO0406

Fig. 27A–G

Thecturota tenuissima Casey, 1893
Atheta marchii Dodero, 1922, syn. nov.
Pragensiella magnifica Machulka, 1941, syn. nov.
Thecturota marchii : Muona 1984 (as valid species)
Thecturota magnifica : Schülke and Smetana 2015 (as syn. of T.marchii)

##### Material

**(DNA-barcoded specimens). Germany**: Kobern-Gondorf, Ortslage/Weinberge, 50.308, 7.460, 21.V.2010, F. Koehler (1, ZSM); Edenkoben-Rhodt, Villastrasse, 49.279, 8.092, 20.X.2012, F. Koehler (1, ZSM). **Finland**: Oba: Oulu, Linnanmaa, 65.0633, 25.4712, botanical garden, compost heap, flight-intercept trap, 7.VI.2011, M. Pentinsaari (1, ZMUO).

##### Non-sequenced material.

Several males and females of *T.tenuissima* from Denmark (NMHD) were compared with illustrations from Klimaszewski et al. (2017).

##### Distribution.

**Origin.** Nearctic (adventive in West Palaearctic). **Canada**: ON, QC. **United States**: RI.

##### Bionomics.

Canadian specimens were collected by car-netting in mixedwood forests, while Palaearctic specimens are known from compost and other plant-based debris (Horion 1967).

##### Comments.

*Thecturotatenuissima* is native to the Nearctic region and has become accidentally introduced to the West Palaearctic, including the Canary Islands, where it was previously known under the synonym *T.marchii* (Newton 2019). We expect this species to be broadly distributed in eastern North America and has been overlooked over much of its range because car-netting, an effective method for collecting small, obscure staphylinids, is rarely used in the Nearctic region.

Nearctic and Palaearctic populations do not differ in male and female genitalia or in external morphology. Molecular data were unavailable for the Nearctic population, which was recently reported from Canada (Klimaszewski et al. 2017) but described from Rhode Island, USA in 1893 (Casey 1893). However, we are confident that these species are synonyms. Muona (1984) stated that *T.marchii* is a ‘recent’ introduction to Europe but from an unknown source. *Thecturota* is primarily a New World genus, with ten described species in North and South America (Newton 2019). *Thecturotamagnifica* (Machulka) is currently treated as a synonym of *T.marchii* (Newton 2019) and we simply transfer this name to synonymy with *T.tenuissima*. The only Palaearctic species remaining is poorly known *T.williamsi* (Bernhauer, 1936), known only from the type collected in Great Britain and probably a synonym of *T.tenuissima*. The characters Bernhauer (1936) gave to separate his species from *T.tenuissima* (as *T.marchii*) are slight differences in coloration and body proportions, which are both highly variable in the Palaearctic specimens of *T.tenuissima* studied. Therefore, we consider *T.tenuissima* to be a native Nearctic species that has become adventive in the West Palaearctic and suggest that genus *Thecturota* is naturally restricted to the Nearctic and Neotropical regions.

**Figure 27.**
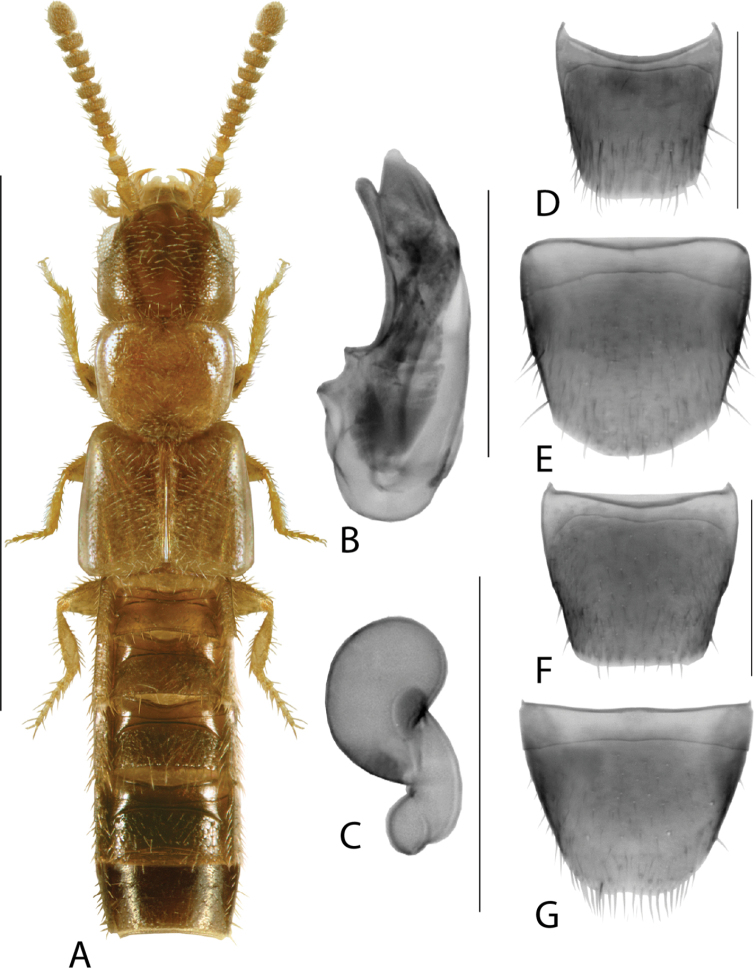
*Thecturotatenuissima* Casey **A** habitus **B** median lobe of aedeagus in lateral view **C** spermatheca **D** male tergite VIII **E** male sternite VIII **F** female tergite VIII **G** female sternite VIII. Scale bars: 1 mm (**A**); 0.2 mm (**B–G**). Illustrations after Klimaszewski et al. (2017).

### Geostibini Seevers, 1978

#### Aloconota pseudogregaria

Taxon classificationAnimaliaColeopteraStaphylinidae

Klimaszewski, Brunke & Pentinsaari sp. nov.

5EBBE4DD-F416-5D20-97E9-CB17191D5A7C

http://zoobank.org/A72E1E63-3D1B-4CC8-882C-6921E5FC3D33

BOLD:AAY6554

Fig. 28A–G

##### Type material.

***Holotype*** (male) (CNC): **Canada: ON**: Waterloo County, Cambridge, Rare Charitable Research Reserve, 43.390, -80.374, soybean field, pitfall trap, 29.VI.2010, A. Brunke [white printed label] / HOLOTYPE *Aloconotapseudogregaria* Klimaszewski, Brunke & Pentinsaari sp. nov., des A. Brunke 2020 [red printed label]. ***Paratypes* (1 NMNH, 6 DEBU, 1 CNC): Canada: ON**: Wellington County, Eramosa, 43.616, -80.215, soybean field, pitfall trap, 13.VII.2010, A. Brunke [white printed label] / PARATYPE *Aloconotapseudogregaria* Klimaszewski, Brunke & Pentinsaari sp. nov., des A. Brunke 2020 [yellow printed label] [CNC, DEBU, 7 specimens]. **United States: VA**: Arlington County, Marcey Creek, 38.9087, -77.1083, 70 m, suburban backyard, Malaise trap, 14–21.VI.2015, S. Miller [white printed label] / Barcode of life, DNA voucher specimen, Sample ID: BIOUG42376-E12, Process ID: GMUAF1698-18 [yellow printed label] / PARATYPE *Aloconotapseudogregaria* Klimaszewski, Brunke & Pentinsaari, sp. nov., des A. Brunke 2020 [yellow printed label] [NMNH, 1 specimen].

##### Non-type material

**(barcoded specimens). Canada: Ontario**: Guelph, 25 Division St., 43.554, -80.264, Malaise trap, 14.VII.2010, A. Smith (1, CBG); Guelph, John F. Ross CVI, 43.5621, -80.247, Malaise trap, 22.IV-03.V.2013, G. Staines (1, CBG); Milverton, Milverton Public School, 43.568, -80.928, Malaise trap, 22.IV-03.V.2013, J. Van Bakel (1, CBG); Collingwood, Collingwood Collegiate Institute, 44.489, -80.215, 188 m, Malaise trap, 22.IV-05.V.2014, A. Breton (1, CBG); Cambridge, rare Charitable Research Reserve, 43.3736, -80.3652, 304 m, 04–11.VI.2015, BIO Collections Staff (1, CBG).

##### Etymology.

The species epithet refers to the similarity to related species *A.gregaria* (Erichson), which was originally treated separately from other Aloconota under subgenus Glossola Fowler (e.g., Benick 1954) because it lacks obvious male secondary sexual characters.

##### Diagnosis.

**Figure 28.**
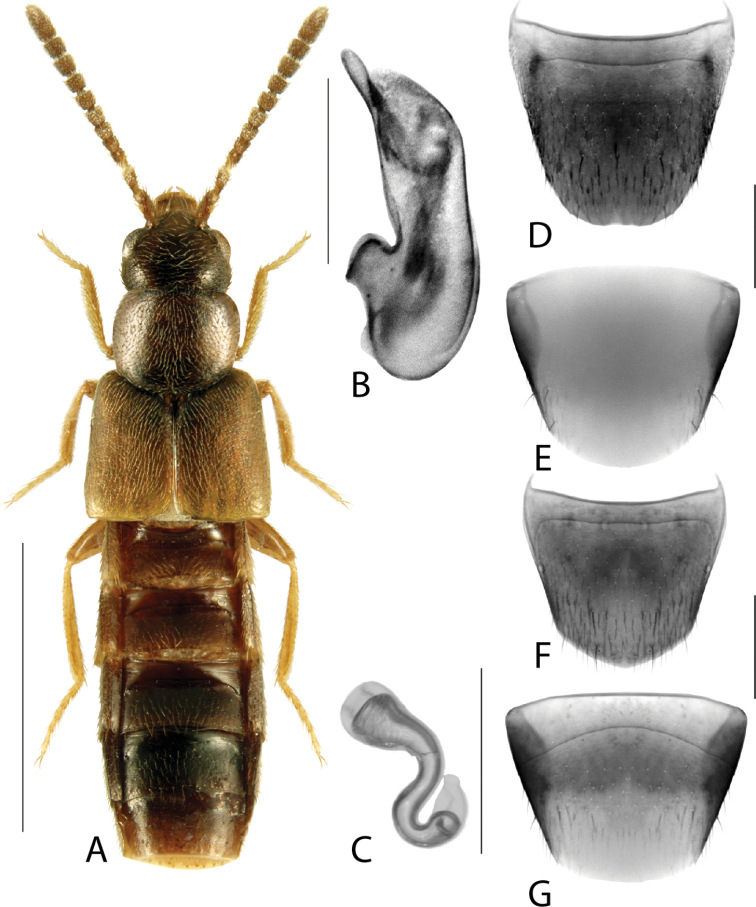
*Aloconotapseudogregaria* Klimaszewski, Brunke & Pentinsaari, sp. nov. **A** habitus **B** median lobe of aedeagus in lateral view **C** spermatheca **D** male tergite VIII **E** male sternite VIII (structure accidentally over-cleared in balsam preparation) **F** female tergite VIII **G** female sternite VIII. Scale bars: 1 mm (**A**); 0.2 mm (**B–G**).

*Aloconotapseudogregaria* can be easily distinguished from all other species of the genus occurring in eastern North America by the distinctly bicolored abdomen (Fig. 28A). Among Central European species, the spermatheca of *A.pseudogregaria* is most similar to that of Palaearctic *A.gregaria* but in the latter the apex is distally truncate, median lobe is distinctly sinuate and only weakly projected ventrad, the abdomen is darker and not distinctly bicolored, and the microsculpture of the forebody is much stronger, creating a dull reflection.

##### Description.

Body length 2.4–2.7 mm, moderately flattened (stronger so on elytra), narrowly subparallel, colour of head, pronotum, scutellar region of elytra, apical part of abdomen and antennomeres 5–11 dark brown to dark reddish brown, elytra and antennomeres 1–3 paler, red-brown and legs yellow; forebody finely and densely punctate, microsculpture shallow, consisting of meshes; head slightly elongate and with small, shallow impression medially, head slightly narrower than pronotum, postocular region elongate, ca. as long as maximum diameter of eye, tempora with carinae dorsally only; antennae slender, as long as pronotum and elytra combined, basal three antennomeres strongly elongate, 4 subquadrate, 5–10 subquadrate to slightly transverse, and terminal one strongly elongate and ca. as long as two preceding antennomeres combined; pronotum slightly transverse (width/length ratio 1.3), trapezoidal in shape, flattened, pubescence directed straight posteriad in central part of disc and obliquely posteriad laterally; elytra at suture ca. as long as pronotum along midline, flat, distinctly transverse (width/length ratio 1.5), ~ 1/3 broader than pronotum, humeri angular, posterior margins slightly sinuate laterally, pubescence directed straight posteriad forming slightly arcuate lines in sutural region of disc; abdomen subparallel, tergites III–VI distinctly impressed at base; basal metatarsomere ~ 1/3 longer than the following one. MALE. Tergite VIII rounded apically with minute median emargination, lacking apical teeth (Fig. 28D); sternite VIII rounded apically (Fig. 28E); tubus of median lobe of aedeagus long, ventrally ca. straight in basal two-thirds and moderately projecting ventrad apically in lateral view (Fig. 28B). FEMALE. Tergite VIII rounded apically and slightly pointed medially (Fig. 28F); sternite VIII rounded apically (Fig. 28G); spermatheca S-shaped, capsule pitcher-shaped with short neck, stem strongly sinuate and swollen apically (Fig. 28C).

##### Distribution.

**Origin**: Nearctic. **Canada**: ON. **United States**: VA.

##### Bionomics.

This species has only been collected by passive traps, including malaise and pitfall traps. All specimens have been collected from at least partly disturbed habitats, such as forest edges, agricultural fields, and suburban environments. This species corresponds to ‘Aleocharinae sp. 5’ in Brunke et al. (2014), which was collected in both soybean fields and adjacent forest edges by pitfall traps.

##### Comments.

*Aloconotapseudogregaria* is probably broadly distributed in northeastern North America. We have compared the male and female genitalia of *A.pseudogregaria* with all Central European and Nearctic species of *Aloconota*, and are confident that this taxon has not been previously described from Europe or North America, despite its occurrence in disturbed habitats in North America, which is typical for introduced species. Although *Aloconotapseudogregaria* clustered most closely with *A.gregaria* (BOLD:ABU6164) in our barcode dataset, its BIN is ~ 8% different from that of the latter. Based on morphology of the aedeagus and spermatheca, *Aloconotapseudogregaria* is probably even more closely related to East Palaearctic *Aloconota* described from Japan and Korea (e.g., Sawada 1970 [as *Tomoglossa*], Lee and Ahn 2017) rather than to *A.gregaria*. However, the described species all differ markedly in external morphology.

### Athetini Casey, 1910

#### Atheta (Datomicra) nigra

Taxon classificationAnimaliaColeopteraStaphylinidae

(Kraatz, 1856)

418FBDEF-289E-530A-8EFF-54B3793EA320

BOLD:ACO4408

Fig. 29A–G

##### Material

**(DNA-barcoded specimens). Canada: Ontario**: Peterborough, 44.253N, 78.415W, farm, malaise trap, 24–30.V.2015, B. McClenaghan (1, CBG). **Germany**: Koeln-Worringen, Worringer Bruch, 51.044, 6.87427, 01.VII.2010, F. Koehler & J. Koehler (1, ZSM).

##### Distribution.

**Origin.** Palaearctic (adventive in North America). **Canada**: ON [new record], SK.

##### Bionomics.

Canadian specimens have been collected on farmland and directly from horse manure.

##### Comments.

*Athetanigra* is a Palaearctic species reported from across Europe, European Russia, Kazakhstan, North Korea and southern China (Newton 2019). It is adventive in North America and New Zealand (Newton 2019) and is here newly reported from Ontario. The new record from Ontario indicates that this species is far more widely distributed in North America than previously known.

**Figure 29.**
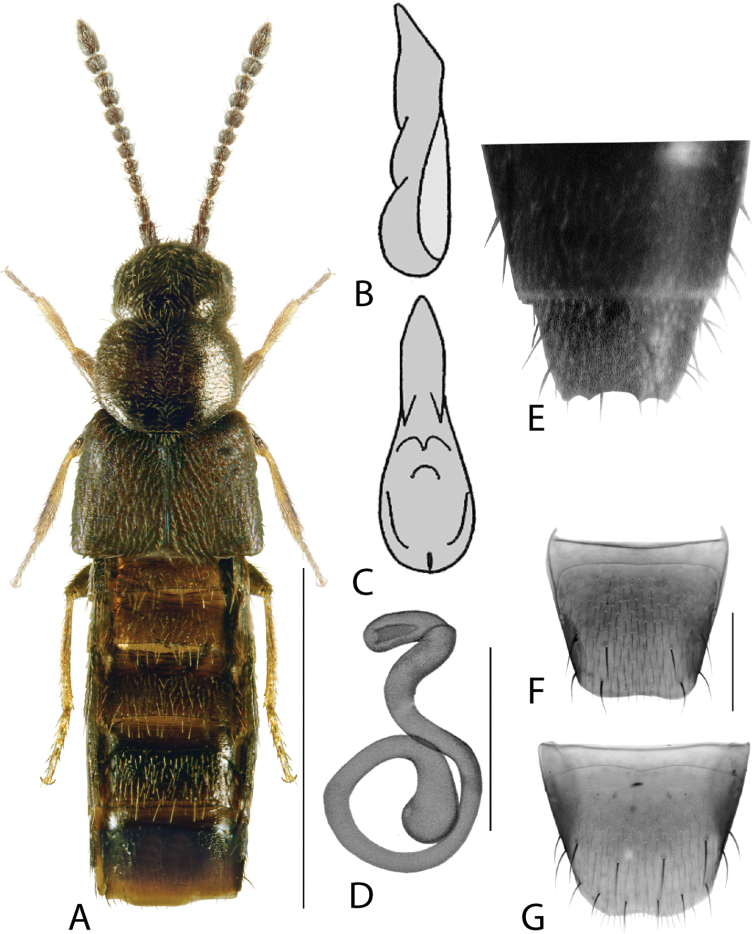
Atheta (Datomicra) nigra (Kraatz) **A** habitus **B** median lobe of aedeagus in lateral view **C** median lobe of aedeagus in ventral view **D** spermatheca **E** apical part of dorsal male abdomen showing 4 dents on tergite VIII **F** female tergite VIII **G** female sternite VIII; **A, D, F, G** after Klimaszewski et al. (2016a), based on female from Saskatchewan, Canada **B, C** after Benick and Lohse (1974)**E** after Klimaszewski et al. (2016a), based on a male from Germany. Scale bars: 1 mm (**A**); 0.2 mm (**B–G**).

#### Mocyta scopula

Taxon classificationAnimaliaColeopteraStaphylinidae

(Casey, 1893) comb. nov.

9906C34F-CC1D-54D1-B1AA-4848C04A897E

BOLD:ACH8720

Fig. 30A–F

Eurypronota scopula Casey, 1893
Pancota laetabilis Casey, 1906
Dolosota abundans Casey, 1910
Dolosota flaccida Casey, 1910
Dolosota redundans tergina Casey, 1910
Dolosota scopula : Casey (1910) (as type species of Dolosota)
Dolosota secunda Casey, 1910
Dolosota sequax Casey, 1910
Acrotona (Dolosota) abundans : Seevers (1978) (as valid species)
Acrotona (Dolosota) flaccida : Seevers (1978) (as valid species)
Acrotona (Dolosota) scopula : Seevers (1978) (as valid species) (Dolosota syn. of Acrotona, in part; some species moved to Pancota)
Acrotona (Dolosota) secunda : Seevers (1978) (as valid species)
Acrotona (Dolosota) sequax : Seevers (1978) (as valid species)
Pancota laetabilis : Seevers (1978) (as valid species)
Pancota redundans tegrina : Seevers (1978) (implied, subspecies not directly mentioned)
Acrotona abundans : Majka and Sikes (2009) (syn. of A.scopula following Gusarov (2003b))
Acrotona flaccida : Majka and Sikes (2009) (syn. of A.scopula following Gusarov (2003b))
Acrotona laetabilis : Majka and Sikes (2009) (syn. of A.scopula following Gusarov (2003b))
Acrotona redundans tergina : Majka and Sikes (2009) (syn. of A.scopula following Gusarov (2003b))
Acrotona scopula : Majka and Sikes (2009) (valid species following Gusarov (2003b))
Acrotona secunda : Majka and Sikes (2009) (syn. of A.scopula following Gusarov (2003b))
Acrotona sequax : Majka and Sikes (2009) (syn. of A.scopula following Gusarov (2003b))

##### Material

**(DNA-barcoded specimens). Canada: Ontario**: Georgian Bay Islands National Park, Fairy Lake, 44.8929, -79.8514, mostly conifer forest with moss, Berlese funnel, 5.VIII.2015, BIObus 2015 (1, CBG).

##### Distribution.

**Origin.** Nearctic. **Canada**: ON [new record]. **United States**: IA, MO, MS, NY, PA, RI.

##### Diagnosis.

**Figure 30.**
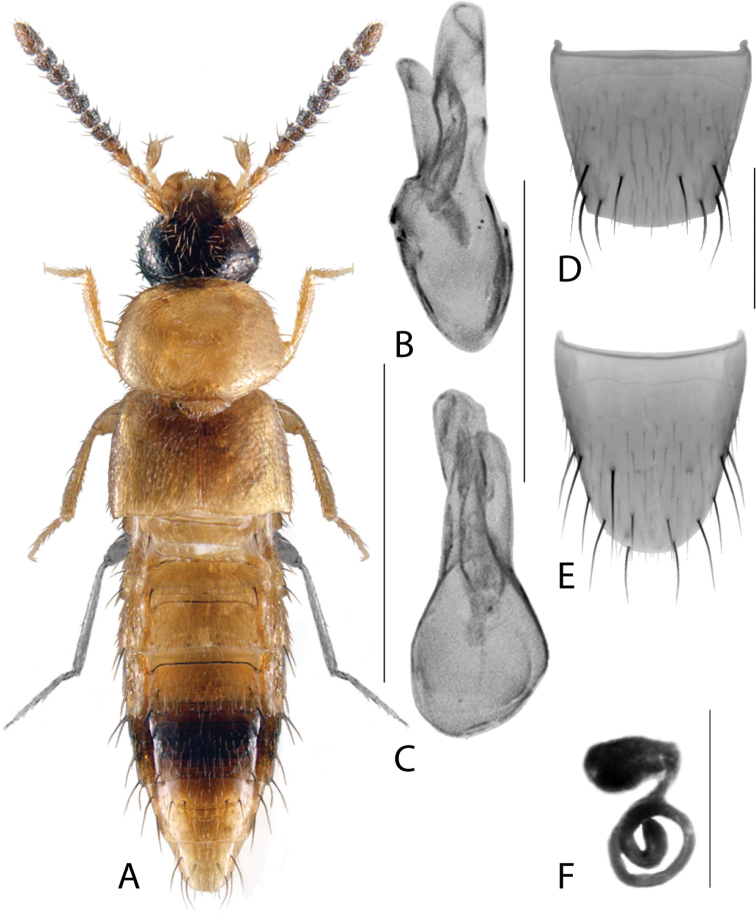
*Mocytascopula* (Casey) **A** habitus (hind legs missing on specimen, taken from related species) **B** median lobe of aedeagus in lateral view **C** median lobe of aedeagus in dorsal view **D** male tergite VIII **E** male sternite VIII **F** spermatheca (female syntype). Scale bars: 1 mm (**A**); 0.2 mm (**B–F**).

*Mocytascopula* can be distinguished from bicolored Canadian species and paler specimens of *M.fungi* by its finely punctate pronotum that is almost as wide as the elytra and ca. as long, and the distinctly transverse antennomeres 6–10 (Fig. 30A). The barcode sequences of *M.scopula* forms a sister cluster with *M.luteola* (BOLD:ABW2813), with a sequence divergence of ~ 7.5%. These species can be easily separated using the above diagnosis.

##### Bionomics.

The Canadian specimen was collected from forest litter with a Berlese funnel but nothing specific is known about this species’ microhabitat preferences.

##### Comments.

*Mocytascopula* is a native Nearctic species distributed in eastern North America. Here we newly report it from Canada based on one male specimen collected in southern Ontario. Its distribution in the United States is based on type material, including its putative synonyms, which should be verified.

*Mocytascopula* is the type species of Dolosota Casey, which has been treated as a subgenus of Acrotona since Seevers (1978). However, using the generic concepts of Klimaszewski et al. (2015), this species best fits in genus *Mocyta* based on the following character states: dorsal surface without fine white pubescence; broad tergite VIII with basal line not touching base of tergite; spermatheca with pear-shaped capsule and distinct but small invagination, and thin and irregularly shaped stem ending in a tightly deflexed apex (Fig. 30F) (based on images taken of female syntypes (NMNH). Further evidence comes from barcode sequences of this species, which cluster with the other species of *Mocyta*. Therefore, we synonymize *Dolosota* Casey syn. nov. with *Mocyta* Mulsant and Rey. The other species included in *Dolosota* by Seevers (1978) were treated as synonyms of *M.scopula* by Majka and Sikes (2009), in addition to two other Casey names (see above synonymy), following the unpublished results of a type revision by V. Gusarov (Gusarov 2003b). These synonyms and *M.scopula* are here comb. nov. in *Mocyta*.

The aedeagus, coloration and punctation of the Canadian specimen are consistent with type material of *M.scopula*, previously examined and imaged by JK. The two other members of the BIN BOLD:ACH8720 originate from a study by Elven et al. (2010), and were mined into BOLD from GenBank. They were collected in the USA and identified verbatim as *Mocytascopula* by V. Gusarov.

### The key to Canadian *Mocyta* in Klimaszewski et al. (2015) can be modified as follows (bicolored species)

**Table:** 

2a	Pronotum much broader than elytra; antennal articles 5–10 in specimens slightly elongate; spermatheca forming concentric circles posteriorly	***M.discreta* (Casey)**
–	Pronotum ca. as broad as elytra or slightly narrower (Fig. 30A); antennal articles 5–10 subquadrate to transverse (Fig. 30A); spermatheca forming irregular coils posteriorly	**2b**
2b	Pronotum coarsely punctate and extremely transverse with weakly rounded base and apex; antennal articles 5–10 subquadrate; median lobe in lateral view strongly produced ventrad	***M.luteola* (Erichson)**
–	Pronotum finely punctate and transverse, but more rounded at base and apex (Fig. 30A); antennal articles 5–10 distinctly transverse (Fig. 30A); median lobe in lateral view only weakly produced ventrad (Fig. 30B)	***M.scopula* (Casey)**

#### Philhygra angusticauda

Taxon classificationAnimaliaColeopteraStaphylinidae

(Bernhauer, 1909)

353C13CE-E3C2-5AD8-B9EC-0A6955E1EA3D

BOLD:ACG2845

Fig. 31A–G

Atheta (Metaxya) angusticauda Bernhauer, 1909
Atheta (Philhygra) pinegensis Muona, 1983, syn. nov.

##### Material

**(DNA barcoded specimens). Canada: Alberta**: Jasper National Park, Miette Hotsprings, 53.124, -117.7755, Malaise trap placed in valley with creek bed, sides rocky and mossy, 1439 m, 21.VII.2012, BIObus 2012 (1, CBG). **Finland**: Lkoc: Muonio, Sarvijärvi, 68.0909, 24.103, 11.VII.2012, M. Pentinsaari (2, ZMUO).

##### Distribution.

**Origin.** Holarctic. **Canada**: AB[new record], BC, NB. **United States**: AK, NH.

##### Bionomics.

As with other species of the genus, *P.angusticauda* is associated with riparian habitats.

##### Comments.

*Philhygraangusticauda* is a Holarctic species that was previously recognized in the Palaearctic (Finland, Norway, European Russia, Russian Far East) (Schülke and Smetana 2015; Newton 2019) under the synonym *P.pinegensis* (Muona). We newly report this species from Alberta and suggest that it broadly occurs across northern Canada. Specimens from the Nearctic and Palaearctic were found to have identical genitalia and their DNA barcodes form a cluster with only 0.3% divergence between Finnish and Canadian specimens.

**Figure 31.**
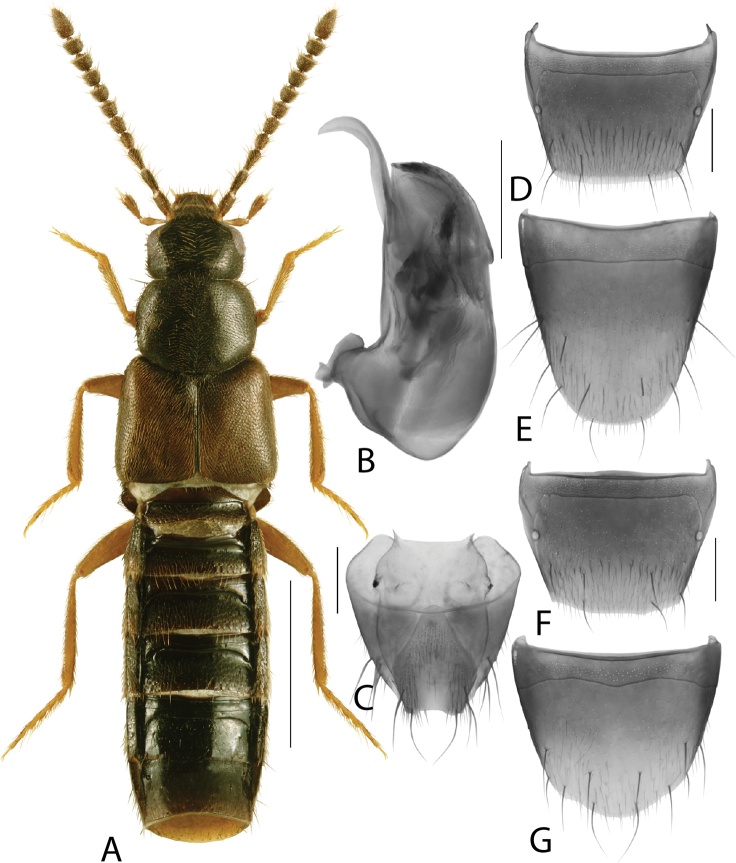
*Philhygraangusticauda* (Bernhauer) **A** habitus **B** median lobe of aedeagus in lateral view **C** female pygidium **D** male tergite VIII **E** male sternite VIII **F** female tergite VIII **G** female sternite VIII. Scale bars: 1 mm (**A**); 0.2 mm (**B–G**). Illustrations after Klimaszewski et al. (2018), reproduced with permission.

#### Philhygra finitima

Taxon classificationAnimaliaColeopteraStaphylinidae

(Casey, 1910)

9054FA71-A57B-50B4-A7F4-0F4AB4589FED

Fig. 32A–H

##### Material

**(non-sequenced specimens). Canada: Ontario**: Algonquin Park, ~45.87, -77.33, car net, 20.VII.2016, T. Struyve (10, CNC, LFC [4 males, 6 females])

##### Distribution.

**Origin.** Nearctic. **Canada**: ON [new record]. **United States**: MA, RI.

##### Diagnosis.

**Figure 32.**
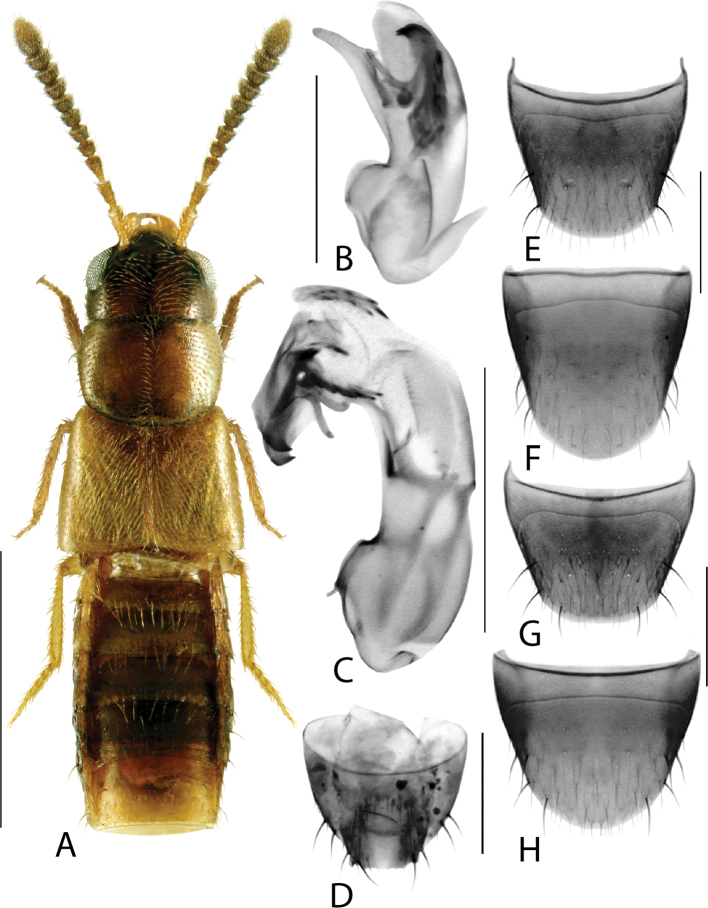
*Philhygrafinitima* (Casey) **A** habitus **B, C** median lobe of aedeagus in lateral view **C** with internal sac everted (unpublished lectotype) **D** female pygidium **E** male tergite VIII **F** male sternite VIII **G** female tergite VIII **H** female sternite VIII. Scale bars: 1 mm (**A**); 0.2 mm (**B–H**).

This species can be readily recognized by a combination of its small size, large eyes and relatively simple, ventrally projecting median lobe of the aedeagus in lateral view (Fig. 32B).

##### Bionomics.

Nothing specific is known about this species’ microhabitat preferences but it probably occurs near water as do other species of the genus. The series of Ontario specimens was collected using a car net, which is typically effective for collecting small staphylinids.

##### Comments.

*Philhygrafinitima* is a native Nearctic species distributed in northeastern North America. Here, we newly report it from Canada. Canadian specimens were identified based on comparison with images (Fig. 32C) of the unpublished lectotype of *P.finitima* in the Casey collection at NMNH.

#### Philhygra laevicollis

Taxon classificationAnimaliaColeopteraStaphylinidae

(Mäklin, 1852), sensu nov.

7178546D-E5F2-598E-BE22-1A01EA8C11B9

BOLD:ACU6301

Fig. 33A–I

##### Material

**(DNA-barcoded specimens). Canada. Alberta**: Waterton Lakes National Park, Highway 6 pulloff, 49.065, -113.779, 1569 m, intercept trap, montane forest, 21–27.VI.2012, BIOBus 2012 (1, CBG); same data except 06–11.VIII.2012 (1, CBG). **British Columbia**: Prince George, Nukko Lake Elementary School, EPQ-CLL-574, 54.083, -122.988, 764 m, 8.V.2015, H. Sapun (1, CBG). **United States. Alaska**: Dall Island, 54.998, -133.016, 15.VII.2011, D. S. Sikes (1, UAM); Prince of Wales Island, Luck Point, 55.98, -132.772, clear cut, berlese, 9.VIII.2011, J. Stockbridge and B. Wong (1, UAM).

##### Distribution.

**Origin.** Nearctic. **Canada**: AB [new record], BC. **United States**: AK, WA.

##### Diagnosis.

**Figure 33.**
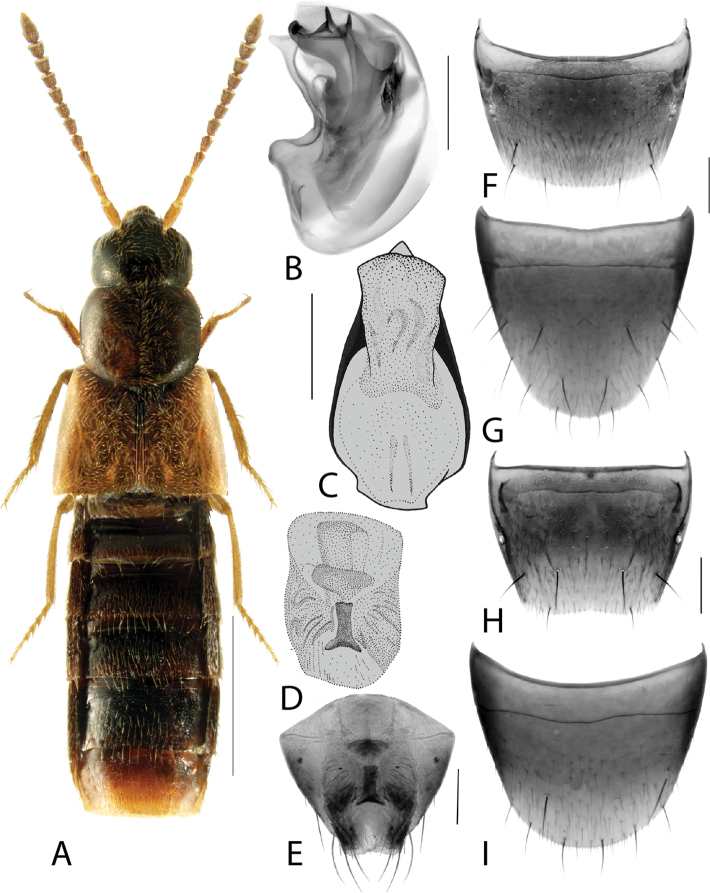
*Philhygralaevicollis* (Mäklin) **A** habitus **B** median lobe of aedeagus in lateral view **C** median lobe of aedeagus in ventral view **D, E** female pygidium **F** male tergite VIII **G** male sternite VIII **H** female tergite VIII **I** female sternite VIII. **A, B, E–H** after Klimaszewski et al. (2020), used with permission **C, D** after Klimaszewski and Winchester (2002).Scale bars: 1 mm (**A**); 0.2 mm (**B–I**).

*Philhygralaevicollis* can be distinguished from most species of the genus by the general shape of the median lobe in lateral view. It is most similar to *P.pseudolaevicollis* but has a sinuate ventral face of the median lobe in lateral view and large spines in the internal sac (Fig. 33B).

##### Bionomics.

Specimens have been collected from clear cut areas, transitional zone of a coniferous forest, seepages, and river and creek edges, from moss, leaf litter, gravel, dung, carrion and pitfall traps (Klimaszewski et al. 2020).

##### Comments.

*Philhygralaevicollis* is a western Nearctic species that was previously considered to include eastern populations that we here treat as *Philhygrapseudolaevicollis* sp. nov. that differs in male genitalia but also by the divergent DNA barcode sequence.

Neither this species nor *P.laevicollis* are known from MB, this error was corrected by Klimaszewski et al. (2020). We have observed some variation in the shape of the sclerotized structure present on the female pygidium between specimens collected in BC, but it is not yet clear whether additional species are overlooked within the present concept of *P.laevicollis*.

#### Philhygra palustris

Taxon classificationAnimaliaColeopteraStaphylinidae

(Kiesenwetter, 1844)

17854478-C200-53E7-B229-61B2137A9C2E

BOLD:AAN6150

Fig. 34A–H

##### Material

**(DNA-barcoded specimens). Canada: Ontario**: Puslinch, Hanner property, 43.4464, -80.2512, Malaise trap in hardwood forest, 21.VIII.2008, T. Terzin (1, CBG); Puslinch, concession 11/Hume Rd., 43.537, -80.134, Malaise trap in temperate mixed forest, 18–24.IV.2010, P. Hebert (1, CBG); Milverton, Milverton Public School, 43.568, -80.928, Malaise trap, 3.V.2013, J. Van Bakel (1, CBG); Cambridge, rare Charitable Research Reserve, Hogsback forest, 43.3729, -80.354, edge of hardwood forest, intercept trap, 31.V.2015, BIO collections staff (2, CBG); same except pan traps (1, CBG); Kawartha Lakes, 44.366, -78.478, farm, Malaise trap, 13.VI.2015, B. McClenaghan (2, vouchers not preserved); Guelph, Arkell Research Station, 43.5187, -80.1709, between corn and soy fields, w/ nearby pasture, Malaise trap, 8.V.2015, BIO collections staff (1, CBG); same except soy field, 43.5264, -80.1796, 4-headed SLAM trap, 17.V.2017 (1, CBG); Hamilton, Royal Botanical Gardens, Cootes Paradise, 43.281, -79.904, forest, deadwood and UV lights at night, 21.VII.2017, M. Pentinsaari (1, CBG); Markham, 43.9371, -79.2285, mixed habitat, Berlese funnel, 25.VI.2017, Rouge NUP BioBlitz Volunteers (1, CBG). **Belgium**: Sint-Genesius-Rode, BR Zonienwoud, 50.7505, 4.423, 16.VI.2010, F. Koehler (1, ZSM). **Estonia**: Piusa, 57.844, 27.466, 05.VII.2010, J. Salokannel (2, ZMUO). **Finland**: Ab: Nauvo, Sandö, 60.1747, 22.1338, 18.VI.2011, M. Pentinsaari (1, ZMUO); Ok: Vaala, Manamansalo, 64.3365, 27.0879, 21.VIII.2011, M. Pentinsaari (1, ZMUO); Ks: Kuusamo, Oulanka, 66.3686, 29.3188, 07.VIII.2011, M. Pentinsaari (1, ZMUO); Ka: Virolahti, Hailiniemi, 60.5259, 27.7366, 20.VII.2012, M. Pentinsaari (1, ZMUO). **Germany**: Riedlhuette, Diensthuettenstrasse, 48.937, 13.412, 09.VII.2011, F. Koehler and M. Koehler (2, ZSM); Spiegelau, Schwarzachstrasse, 48.9456, 13.3619, 09.VII.2011, F. Koehler and M. Koehler (2, ZSM); Waldhaeuser, Lusen- und Boehmstrasse, 48.93, 13.492, 09.VII.2011, F. Koehler and M. Koehler (2, ZSM); Arnsberg-Breitenbruch, NWZ Hellerberg, 51.4461, 8.13539, 30.V.2011, F. Koehler (2, ZSM); Bornheim-Hemmerich, Hellenmaar, 50.7402, 6.91803, 14.VIII.2012, F. Koehler (1, ZSM); Erftstadt-Bliesheim, NWZ Altwald Ville, 50.7917, 6.84384, 03.VI.2011, F. Koehler (1, ZSM); Kandel, Bienwald, 49.01, 8.103, 05.VI.2010, F. Koehler (1, ZSM); Eisenach, E, Rothenhof, Hoerselufer, 50.9643, 10.3644, 06.VII.2013, GBOL-Team ZFMK (2, ZFMK).

##### Distribution.

**Origin.** Palaearctic (adventive in North America). **Canada**: MB, ON [new record]. **USA**: CT, MA, ME, NH, NY, PA, RI, SC, VT, WI.

##### Diagnosis.

**Figure 34.**
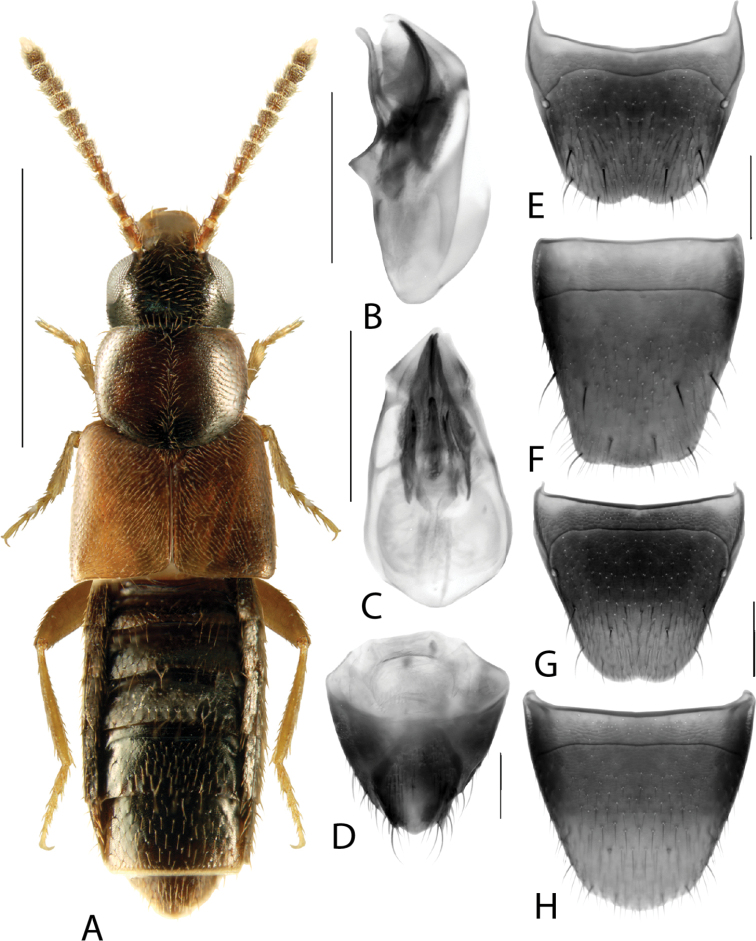
*Philhygrapalustris* (Kiesenwetter) **A** habitus **B** median lobe of aedeagus in lateral view **C** median lobe of aedeagus in ventral view **D** female pygidium **E** male tergite VIII **F** male sternite VIII **G** female tergite VIII **H** female sternite VIII. Scale bars: 1 mm (**A**); 0.2 mm (**B–H**).

Males of this species are easily recognized among other Canadian *Philhygra* by the simple, non-projecting median lobe in lateral view (Fig. 34B).

##### Bionomics.

Most specimens of this species were collected by passive traps in a variety of habitats. In Sweden, *P.palustris* is considered a eurytopic species that occurs in various types of decaying plant matter, including compost, seaweed and hay piles, and along muddy shores of water bodies (Palm 1970). It can be collected in very large numbers using a car net (V. Assing, *pers. comm.*).

##### Comments.

*Philhygrapalustris* is a Palaearctic species that has become adventive and widespread in eastern North America. In the Palaearctic, it is very broadly distributed and reported from Europe, North Africa (Morocco), Russia (European and Siberia), Mongolia, North and South Korea (Lee and Ahn 2012), Japan, and northern China (Newton 2019). It is also known from the Azores and the Canaries (Newton 2019), though it is likely introduced there as well.

This species was reported from Canada (Manitoba) for the first time in the checklist by Bousquet et al. (2013) but no specimens could be found in the CNC to support this record. It is likely that the species *P.tenuicula* (Casey, 1911) described from Manitoba and treated as a synonym of *P.palustris* (Newton 2019), is the basis of this record. *Philhygrapalustris* was first recorded from North America by Muona (1984) from New York, Maine and Pennsylvania but detailed specimen level data were not provided. Several specimens from various localities in southern Ontario have been sequenced, and their barcodes cluster with European specimens, with multiple haplotypes shared between Canada and Europe. This common European species is here confirmed to be adventive in Canada and is probably broadly distributed in at least eastern North America.

#### Philhygra pseudolaevicollis

Taxon classificationAnimaliaColeopteraStaphylinidae

Klimaszewski, Brunke & Pentinsaari sp. nov.

3C47DBC7-7186-5657-8D36-0DA42F3B1270

http://zoobank.org/7D087111-AC74-4D0C-851D-801BA3206497

Fig. 35A–G

##### Type material.

***Holotype* (male) (CNC)**: NEW BRUNSWICK. YORK CO: New Maryland, Charters Settlement, 45.8341°N, 66.7445°W, 22 April 2005, R.P. Webster coll. / mature spruce and cedar forest, seepage area, in saturated sphagnum and leaf litter / HOLOTYPE *Philhygrapseudolaevicollis* Klimaszewski, Brunke & Pentinsaari sp. nov., des. Klimaszewski 2021 [red printed label]. ***Paratypes* (12: LFC, CNC)**: same data as holotype (1 male, CNC). **Canada, New Brunswick**, York Co., New Maryland, Charters Settlement, 45.8331°N, 66.7410°W, 14.04.2005, mixed forest in litter and sphagnum, R.P. Webster (1 male, LFC); York Co., New Maryland, Charters Settlement, 45.8390°N, 66.7308°W, 18.04.2005, mixed forest under bark, R.P. Webster (1 male, LFC); York Co., New Maryland, Charters Settlement, 45.8428°N, 66.7279°W, 20.04.2005, mixed forest small sedge marsh in moist grass litter and sphagnum, R.P. Webster (2 females, LFC, 1 female CNC); York Co., New Maryland, Charters Settlement, mixed forest, near small shaded brook, in leaf litter and moss, 9.05.2005, R.P. Webster (1 female, CNC); York Co., Canterbury Trail to Browns Mtn. Fen, 45.8978°N, 67.6273°W, mature cedar forest near stream, sifting leaf litter, 02.05.2005, M. Giguere and R. Webster (1 male, CNC); Northumberland Co., Goodfellow Brook Protected Area, 46.8943°N, 65.3796°W, old growth, wet eastern cedar swamp, in litter and moss on hummocks, near water, 23.05.2007, R.P. Webster (1 female, CNC). **Quebec**, Scotstown, 28.04.2008, C. Levesque, Barcode sample, BCO1 vial #X16, 26.05.2010, R. Civade (1 male, LFC). **Ontario**, Nipissing Co., Algonquin Prov. Park near Brent, 19.08.1980, R. Baranowski (1 female, LFC); same except: 21.08.1980 (1 male, LFC).

##### Non-types

**(DNA-barcoded specimens). Canada: New Brunswick**: Restigouche Co., 9 km S of Saint Arthur, 47.818, -66.756, eastern white cedar swamp, in moss and litter near small ponds, 14.VI.2006, R.P. Webster (1, cRW).

##### Etymology.

Prefix -*pseudo* meaning false/not genuine, added to the sibling species name *P.laevicollis* (Mäklin).

##### Diagnosis.

**Figure 35.**
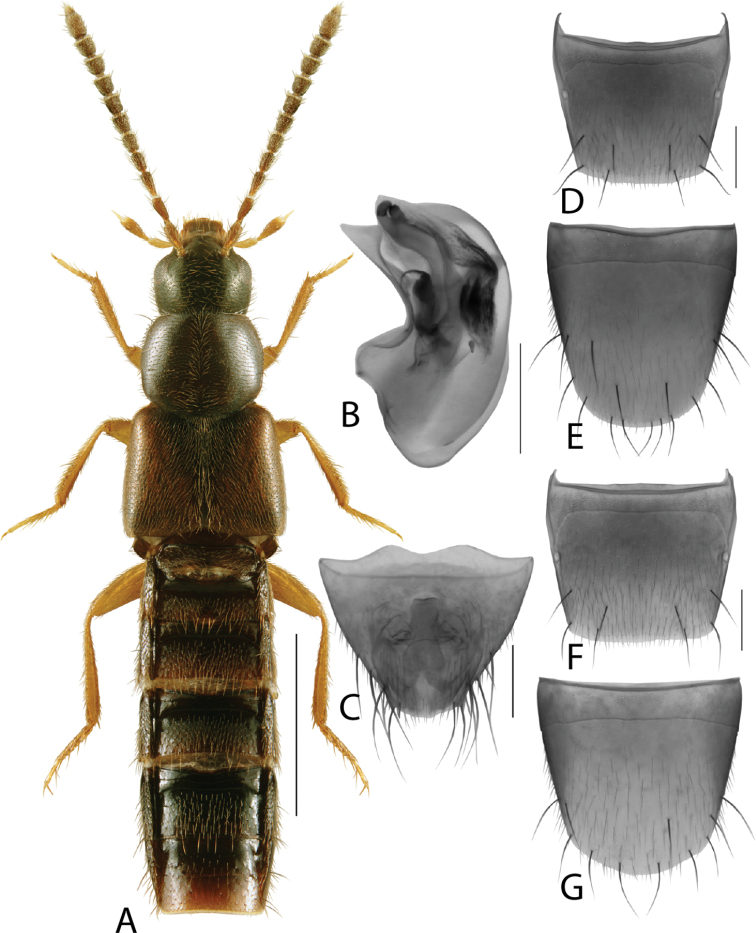
*Philhygrapseudolaevicollis* Klimaszewski, Brunke & Pentinsaari, sp. nov. **A** habitus **B** median lobe of aedeagus in lateral view **C** female pygidium **D** male tergite VIII **E** male sternite VIII **F** female tergite VIII **G** female sternite VIII. Scale bars: 1 mm (**A**); 0.2 mm (**B–G**). Illustrations after Klimaszewski et al. (2018), reproduced with permission.

This species is similar externally and genitally to *P.laevicollis* but may be distinguished from it by the following combination of characters: body on average narrower, antennomeres 6–7 more elongate (Fig. 35A), ventral margin of tubus of the median lobe of aedeagus straight apically (Fig. 35B) (sinuate in *P.laevicollis*, Fig. 33B), apical sclerites of internal sac without large spike-like projections (Fig. 35B).

##### Description.

Body narrowly subparallel, moderately flattened, length 3.0–4.2 mm; colour dark brown, elytra dark brownish to brownish yellow, except for darker scutellar area and paler legs, basal antennomeres rust-brown (Fig. 35A); integument moderately glossy, forebody sparsely punctate and pubescent, with pubescence long, punctation fine, microsculpture distinct and consisting of round and slightly convex meshes; head slightly elongate, round, ca. as wide as pronotum, eyes as long as genae in dorsal view, postocular carina strong basally, diffuse apically; antennae slender, at least as long as pronotum and elytra combined, antennomeres 1–3 strongly elongate, 6 and 7 slightly elongate, 8 and 9 slightly elongate or subquadrate, and terminal one as long as two preceding antennomeres combined; pronotum slightly transverse, impressed medially, arcuate laterally and basally, pubescence sparse, hypomeron visible almost for entire length of pronotum; elytra transverse, broader than pronotum; abdomen subparallel. MALE. Tergite VIII slightly transverse, arcuate apically (Fig. 35D); sternite VIII highly elongate, rounded apically and with wide distance between antecostal suture and base of disc (Fig. 35E); median lobe of aedeagus with moderate-sized bulbus, tubus short, ventral margin arcuate basally and straight apically, tubus narrowly triangular at apex in lateral view (Fig. 35B); internal sac sclerites without spike-like projections, complex as illustrated (Fig. 35B); in dorsal view bulbus roughly oval, tubus short, triangular apically. FEMALE. Tergite VIII transverse and truncate apically (Fig. 35F); sternite VIII rounded apically, apex slightly produced, distance between antecostal suture and base of disc wide (Fig. 35G); pygidium as illustrated, with weakly sclerotized central plate, slightly broader than in *P.laevicollis* (Fig. 35C); spermatheca not illustrated, minute with short sac-shaped capsule without apical invagination and with short narrow stem.

##### Distribution.

**Origin.** Nearctic. **Canada**: NB, NS, ON, QC.

##### Bionomics.

This species has been recorded from various wetland and riparian habitats in NB: in moss and leaf litter near brook and in litter, grasses, and moss on hummocks in old-growth eastern white cedar swamps and a wet alder swamp, in moist leaves along vernal pond margins in various mixed forests, and a red oak/red maple forest; also from pitfall traps in regenerating red spruce forests (NB) and from vernal pool litter in ON (summarized by Klimaszewski et al. 2018). **Collecting period**: IV-V, VIII. **Collecting method**: sifting leaf litter, grasses, and moss, under bark (probably overwintering).

##### Comments.

Although they were not re-examined here, the specimens reported by Majka and Klimaszewski (2008) as *P.laevicollis*, certainly belong to *P.pseudolaevicollis.* This species is very similar externally and genitally to *P.laevicollis* occurring in western North America (AK, AB, BC, WA). Previously, it was tentatively identified as *P.laevicollis* pending additional study (e.g., Klimaszewski et al. 2005; Klimaszewski et al. 2020). The present evidence from DNA barcodes (8.5% divergence between the eastern and western specimens) and morphology of the aedeagus revealed that eastern and western populations represent two distinct, cryptic species. The single barcoded specimen of *P.pseudolaevicollis* produced a 407 bp sequence and therefore, no BIN has been generated.

#### Trichiusa robustula

Taxon classificationAnimaliaColeopteraStaphylinidae

Casey, 1893

3DA98EAA-0B4A-5E7E-80FB-E1B0A447C7B9

BOLD:AAY6555

Fig. 36A–I

Trichiusa robustula Casey, 1893
Trichiusa immigrata Lohse, 1984, syn. nov.

##### Material

**(DNA-barcoded specimens). Austria**: Innervillgraten, Arntal, 46.8362, 12.3348, mountain forest and alpine pastures, car net, 25.VIII.2013, GBOL-Team ZFMK (1, ZFMK). **Germany**: Nuernberg, N Flughafen, 49.5006, 11.0789, sifting compost, date not provided, GBOL-Team ZFMK (1, ZFMK); Schoenau/Hoersel, W, Gewerbegebiet, 50.947, 10.4214, sifting compost, 25.VIII.2012, GBOL-Team ZFMK (2, ZFMK); Kahlenberg/Eisenach, Pferdekoppel, 50.9469, 10.4287, in horse dung, 16.IX.2013, GBOL-Team ZFMK (1, ZFMK). **Finland**: Ka: Hamina, Meltti, 60.5798, 27.2016, 12.X.2011, M. Pentinsaari (1, ZMUO); Al: Mariehamn, Dalen, 60.0703, 19.9595, 9.X.2011, M. Pentinsaari (2, ZMUO). **Canada: Ontario**: Guelph, Division Street, 43.5544, -80.2644, malaise trap, 14.VII.2010, A. Smith (1, CBG); Chelsey, Chelsey District Community School, EQP-CLL-581, 44.3028, -81.0967, 281 m, malaise trap, 22.IX–3.X.2014, A. Grieve (1, CBG); Guelph, Arboretum, Urban Organic Farm, 43.5381, -80.222, compost heaps/mouldy hay pile, 17.IX.2017, M. Pentinsaari (6, CBG).

##### Additional material

**(non-barcoded).** Numerous dissected specimens from Denmark were examined in the collection of NHMD.

##### Distribution.

**Origin.** Nearctic (adventive in Europe). **Canada**: ON, NB. **United States**: IA.

##### Bionomics.

In its native range, this species has been collected in a variety of decaying plant matter, especially near water. This species was also common in compost in NB. In Europe, this species has been collected from similar microhabitats including grass clippings and compost (Denton 1998; Anderson and Bryan 2012).

##### Comments.

*Trichiusarobustula* is a Nearctic species that is broadly distributed in eastern North America but not well collected. It was previously recognized under the synonym *T.immigrata* Lohse in the West Palaearctic (Europe, Canary Islands, Madeira; Newton 2019), where it is adventive.

**Figure 36.**
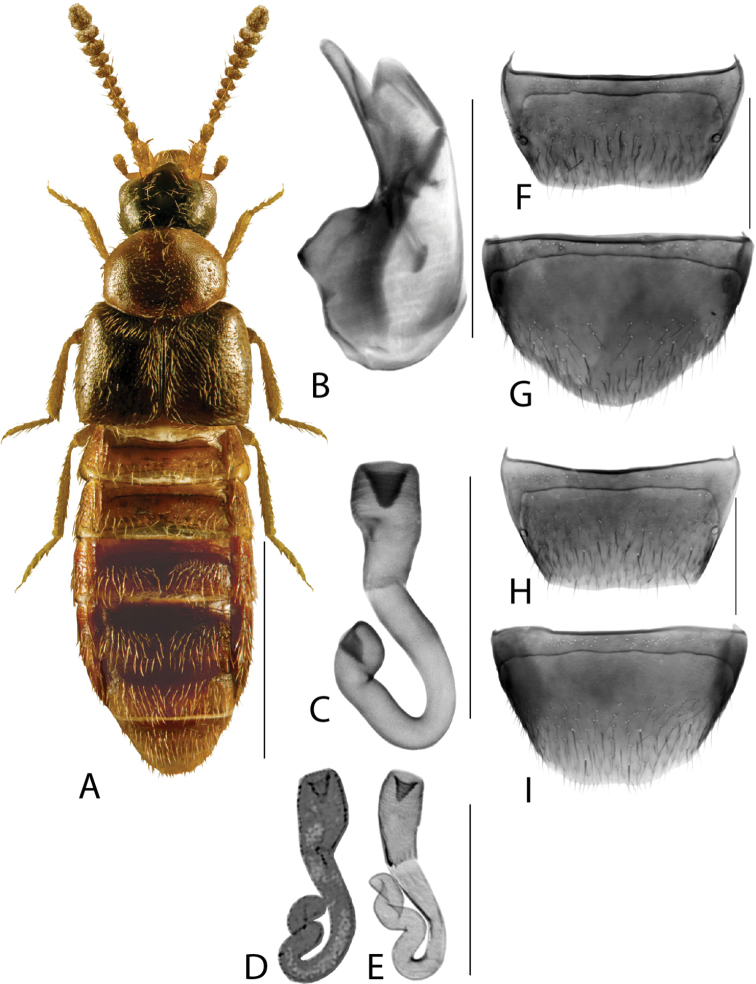
*Trichiusarobustula* Casey **A** habitus **B** median lobe of aedeagus in lateral view **C–E** spermatheca **F** male tergite VIII **G** male sternite VIII **H** female tergite VIII **I** female sternite VIII. **A–C, F–I** after Webster et al. (2016)**D, E** after Brunke et al. 2012. Scale bars: 1 mm (**A**); 0.2 mm (**B–I**).

When describing his new species, Lohse (1984) noted that it must have originated from North America, since *Trichiusa* is otherwise endemic to that region. Lohse (1984) stated that *T.immigrata* was compared with types of North American species described by Casey (1893), but this taxon is a morphological and molecular match to *T.robustula*. Although most of the specimens collected in North America are bicolored (reddish/dark), study of extensive material from Denmark (NMHD) revealed a grade between fully dark brown to reddish/dark bicolored. *Trichiusarobustula* is distinctive for the shape of its spermatheca, which bears a rectangular capsule, and stem that has a single 180-degree bend followed by a twisted apical portion (Fig. 36C). The figure of the spermatheca in Klimaszewski et al. (2018) is atypical (Fig. 36C) and the original illustrations in Brunke et al. (2012), reproduced here (Fig. 36D, E), better show these features.

## Supplementary Material

XML Treatment for Amarochara forticornis

XML Treatment for Haploglossa nebulosa

XML Treatment for Hylota cryptica

XML Treatment for Hylota ochracea

XML Treatment for Isoglossa

XML Treatment for Isoglossa triangularis

XML Treatment for Parocyusa rubicunda

XML Treatment for Paradilacra densissima

XML Treatment for Dasygnypeta baranowskii

XML Treatment for Dasygnypeta nigrella

XML Treatment for Dasygnypeta velata

XML Treatment for Gnypeta impressicollis

XML Treatment for Oligota parva

XML Treatment for Oligota pumilio

XML Treatment for Oligota pusillima

XML Treatment for Anomognathus athabascensis

XML Treatment for Anomognathus cuspidatus

XML Treatment for Cyphea wallisi

XML Treatment for Gyrophaena affinis

XML Treatment for Gyrophaena gracilis

XML Treatment for Gyrophaena simulans

XML Treatment for Homalota plana

XML Treatment for Thecturota tenuissima

XML Treatment for Aloconota pseudogregaria

XML Treatment for Atheta (Datomicra) nigra

XML Treatment for Mocyta scopula

XML Treatment for Philhygra angusticauda

XML Treatment for Philhygra finitima

XML Treatment for Philhygra laevicollis

XML Treatment for Philhygra palustris

XML Treatment for Philhygra pseudolaevicollis

XML Treatment for Trichiusa robustula

## Supplementary materials

Supplementary material 1 Specimen voucher data and corresponding DNA barcode accession numbers Data type specimen data Explanation note All specimen data and sequence accession numbers associated with the DNA barcode dataset used in this study. https://binary.pensoft.net/file/550302This dataset is made available under the Open Database License (http://opendatacommons.org/licenses/odbl/1.0/). The Open Database License (ODbL) is a license agreement intended to allow users to freely share, modify, and use this Dataset while maintaining this same freedom for others, provided that the original source and author(s) are credited.Adam J. Brunke, Mikko Pentinsaari, Jan Klimaszewski

## References

[B1] Anderson R Bryan MD (2012) *Trichiusaimmigrata* Lohse (Staphylinidae) in Ireland. The Coleopterist 21: e94.

[B2] Assing V (2002) A taxonomic and phylogenetic revision of *Amarochara* Thomson. I. The species of the Holarctic region. Beiträge zur Entomologie 52: 111–204. 10.21248/contrib.entomol.52.1.111-204

[B3] Assing V (2021) On the taxonomy of *Parocyusa*, *Tectusa*, and miscellaneous genera of Oxypodina (Insecta: Coleoptera: Staphylinidae: Aleocharinae: Oxypodini). Annalen des Naturhistorischen Museums in Wien 123: 99–218. https://www.jstor.org/stable/26993242?seq=1

[B4] Benick G (1952) Revision der Untergattung *Aloconota* C.G. Thoms. (Gattung *Atheta*, *Staph.*). Entomologische Blatter 50: 133–174.

[B5] Bernhauer M (1936) Neuheiten der paläarktischen Staphylinidenfauna III. Koleopterologische Rundschau 22: 50–58. https://www.zobodat.at/pdf/KOR_22_1936_0050-0058.pdf

[B6] Bousquet Y Bouchard P Davies A Sikes DS (2013) Checklist of Beetles (Coleoptera) of Canada and Alaska. Pensoft, Sofia, 402 pp. 10.3897/zookeys.360.4742 PMC3867111 24363590

[B7] Blackwelder RE (1952) The generic names of the beetle family Staphylinidae, with an essay on genotypy. United States National Museum Bulletin 200: 1–483. https://www.biodiversitylibrary.org/part/85283

[B8] Brunke AJ Buffam J (2018) A Review of Nearctic rove beetles (Staphylinidae) specialized on the burrows and nests of vertebrates. In: Betz O, Irmler U, Klimaszewski J (Eds) Biology of Rove Beetles (Staphylinidae), Life History, Evolution, Ecology and Distribution. Springer, Cham, Switzerland, 295 pp. [145–159.] 10.1007/978-3-319-70257-5_8

[B9] Brunke A Klimaszewski J Dorval J-A Bourdon C Paiero S Marshall S (2012) New species and distributional records of Aleocharinae (Coleoptera, Staphylinidae) from Ontario, Canada, with a checklist of recorded species. ZooKeys 186: 119–206. 10.3897/zookeys.186.2947 PMC3349194 22577320

[B10] Brunke AJ Bahlai CA Klimaszewski J Hallett RH (2014) Rove beetles (Coleoptera: Staphylinidae) in Ontario, Canada soybean agroecosystems: assemblage diversity, composition, seasonality, and habitat use. The Canadian Entomologist 146: 652–670. 10.4039/tce.2014.19

[B11] Brunke AJ Salnitska M Hansen AK Zmudzinska A Smetana A Buffam J Solodovnikov A (2020) Are subcortical rove beetles truly Holarctic? An integrative taxonomic revision of north temperate *Quedionuchus* (Coleoptera: Staphylinidae: Staphylininae). Organisms Diversity & Evolution 20: 77–116. 10.1007/s13127-019-00422-2

[B12] Casey TL (1893) Coleopterological notices. V. Annals of the New York Academy of Sciences 7: 281–606. 10.1111/j.1749-6632.1893.tb55411.x

[B13] Casey TL (1906) Observations on the staphylinid groups Aleocharinae and Xantholinini, chiefly of America. Transactions of the Academy of Science of St. Louis 16: 125–434. https://www.biodiversitylibrary.org/page/33076327

[B14] Casey TL (1910) New Species of the Staphylinid Tribe Myrmedoniini. Memoirs on the Coleoptera 1. New Era Printing Co., Lancaster, Pennsylvania, 184 pp. https://www.biodiversitylibrary.org/page/962781

[B15] Denton M (1998) *Trichiusaimmigrata* Lohse (Staphylinidae) in Yorkshire. Coleopterist 4: 1–14.

[B16] deWaard JR Ratnasingham S Zakharov EV Borisenko AV Steinke D Telfer AC Perez KHJ Sones JE Young MR Levesque-Beaudin V Sobel CN Abrahamyan A Bessonov K Blagoev G deWaard SL Ho C Ivanova NV Layton KKS Lu L Manjunath R McKeown JTA Milton MA Miskie R Monkhouse N Naik S Nikolova N Pentinsaari M Prosser SWJ Radulovici AE Steinke C Warne CP Hebert PDN (2019) . A reference library for Canadian invertebrates with 1.5 million barcodes, voucher specimens, and DNA samples. Scientific Data 6: e308. 10.1038/s41597-019-0320-2 PMC6897906 31811161

[B17] Elven H Bachmann L Gusarov VI (2010) Phylogeny of the tribe Athetini (Coleoptera: Staphylinidae) inferred from mitochondrial and nuclear sequence data. Molecular Phylogenetics and Evolution 57: 84–100. 10.1016/j.ympev.2010.05.023 20554052

[B18] Enushchenko IV Semenov VB (2016) A review of the genus *Gyrophaena* Mannerheim 1830 (Coleoptera: Staphylinidae: Aleocharinae: Gyrophaenina) of the Caucasus and adjacent territories. Zootaxa 4126: 301–337. 10.11646/zootaxa.4126.3.1 27395591

[B19] Erichson WF (1839) Genera et species Staphylinorum insectorum coleopterorum familiae. F.H. Morin, Berlin, 400 pp. https://www.biodiversitylibrary.org/page/39356602

[B20] Fenyes A (1918) Coleoptera: Fam Staphylinidae, subfam. Aleocharinae. In: Wytsman P (Ed.) Genera Insectorum, Fasc 173 A. L. Desmet-Verteneuil, Bruxelles, 110 pp.

[B21] Fenyes A (1921) New genera and species of Aleocharinae with polytomic synopsis of the tribes. Bulletin of the Museum of Comparative Zoology 65: 17–35.

[B22] Gouix N Klimaszewski J (2007) Catalogue of Aleocharine Rove Beetles of Canada and Alaska (Coleoptera, Staphylinidae, Aleocharinae). Pensoft Publishers, Sofia-Moscow, 168 pp.

[B23] Gusarov VI (2003a) Revision of some types of North American aleocharines (Coleoptera: Staphylinidae: Aleocharinae), with synonymic notes. Zootaxa 353: 1–134. 10.11646/zootaxa.353.1.1

[B24] Gusarov VI (2003b) A catalogue of the athetine species of America north of Mexico (Coleoptera: Staphylinidae: Aleocharinae: Athetini). https://web.archive.org/web/20100613213828/http://www.nhm.ku.edu/ksem/peet/catalogs/cataweb.htm

[B25] Hansen A Bogri A Solodovnikov A (2017) The Danish Beetle Bank. www.BilleBank.dk [last accessed on January 19, 2021]

[B26] Hendrich L Morinière J Haszprunar G Hebert PDN Hausmann A Köhler F Balke M (2015) A comprehensive DNA barcode database for Central European beetles with a focus on Germany: Adding more than 3,500 identified species to BOLD. Molecular Ecology Resources 15: 795–818. 10.1111/1755-0998.12354 25469559

[B27] Horion AD (1967) Faunistik der Mitteleuropaïschen Käfer (Vol. II). Staphylinidae, part 3 Habrocerinae bis Aleocharinae (Ohne Subtribus Athetae). P.C.W. Schmidt, Überlingen-Bodensee, 4190 pp.

[B28] Kapp A (2019) Revision der westpaläarktischen Arten der Gattungen Oligota Mannerheim, 1830 und Holobus Solier, 1849 (Coleoptera, Staphylinidae, Aleocharinae, Hypocyphtini). Linzer biologische Beiträge 51: 587–698. 10.5281/zenodo.3754300

[B29] Klimaszewski J (1979) A revision of the Gymnusini and Deinopsini of the world (Coleoptera: Staphylinidae: Aleocharinae). Agriculture Canada Monograph 25: 1–169.

[B30] Klimaszewski J Ashe JS (1991) The oxypodine genus *Haploglossa* Kraatz in North America (Coleoptera: Staphylinidae: Aleocharinae). Giornale Italiano di Entomologia 5: 409–416.

[B31] Klimaszewski J Pelletier G (2004) Review of the *Ocalea* group of genera (Coleoptera, Staphylinidae, Aleocharinae) in Canada and Alaska: new taxa, bionomics and distribution. The Canadian Entomologist 136: 443–500. 10.4039/n03-069

[B32] Klimaszewski J Assing V Majka CG Pelletier G Webster RP Langor DW (2007) Records of adventive aleocharine beetles (Coleoptera: Staphylinidae: Aleocharinae) found in Canada. The Canadian Entomologist 139: 54–79. 10.4039/n05-105

[B33] Klimaszewski J Savard K Pelletier G Webster RP (2008) Species review of the genus *Gnypeta* Thomson from Canada, Alaska and Greenland (Coleoptera, Staphylinidae, Aleocharinae): systematics, bionomics and distribution. ZooKeys 2: 11–84. 10.3897/zookeys.2.4

[B34] Klimaszewski J Webster RP Bourdon C Pelletier G Godin B Langor D (2015) Review of Canadian species of the genus *Mocyta* Mulsant & Rey (Coleoptera, Staphylinidae, Aleocharinae), with the description of a new species and a new synonymy. ZooKeys 487: 111–139. 10.3897/zookeys.487.9151 PMC4366688 25829852

[B35] Klimaszewski J Larson DJ Labrecque M Bourdon C (2016a) Twelve new species and fifty-three new provincial distribution records of Aleocharinae rove beetles of Saskatchewan, Canada (Coleoptera, Staphylinidae). ZooKeys 610: 45–112. 10.3897/zookeys.610.9361 PMC4992812 27587977

[B36] Klimaszewski J Langor D Hammond HE Bourdon C (2016b) A new species of *Anomognathus* and new Canadian and provincial records of aleocharine rove beetles from Alberta, Canada (Coleoptera, Staphylinidae, Aleocharinae). ZooKeys 581: 141–164. 10.3897/zookeys.581.8014 PMC4857044 27199584

[B37] Klimaszewski J Langor D Bourdon C Gilbert A Labrecque M (2016c) Two new species and new provincial records of aleocharine rove beetles from Newfoundland and Labrador, Canada (Coleoptera, Staphylinidae, Aleocharinae). ZooKeys 593: 49–89. 10.3897/zookeys.593.8412 PMC4926630 27408552

[B38] Klimaszewski J Struyve T Bourdon C Dorval J-A (2017) First record of *Thecturotatenuissima* Casey from Canada (Coleoptera, Staphylinidae, Aleocharinae). ZooKeys 702: 19–25. 10.3897/zookeys.702.19963 PMC5673942 29118598

[B39] Klimaszewski J Webster R Langor D Brunke AJ Davies A Bourdon C Labrecque M Newton AF Dorval J-A Frank JH (2018) Aleocharine rove beetles of Eastern Canada (Coleoptera, Staphylinidae, Aleocharinae): a glimpse of megadiversity. Springer Nature, Cham, 902 pp. 10.1007/978-3-319-77344-5

[B40] Klimaszewski J Sikes DS Brunke AJ Bourdon C (2019) Species review of the genus *Boreophilia* Benick from North America (Coleoptera, Staphylinidae, Aleocharinae, Athetini): Systematics, habitat, and distribution. ZooKeys 848: 57–102. 10.3897/zookeys.848.34846 31160880 PMC6536487

[B41] Klimaszewski J Hoebeke ER Godin B Davies A Perry KI Bourdon C Winchester N (2020) Aleocharine rove beetles of British Columbia: a hotspot of Canadian biodiversity (Coleoptera, Staphylinidae). Springer, Cham, 631 pp. 10.1007/978-3-030-36174-7

[B42] Klimaszewski J Brunke AJ Sikes DS Pentinsaari M Godin B Webster RP Davies A Bourdon C Newton AF (in press) A faunal review of aleocharine rove beetles in the rapidly changing Arctic and Subarctic regions of North America (Coleoptera: Staphylinidae). Springer Nature, Cham.

[B43] LeConte JL (1863) List of the Coleoptera of North America. Prepared for the Smithsonian Institution. Smithsonian Miscellaneous Collections 6: 1–56. https://www.biodiversitylibrary.org/page/18078314

[B44] Lee S-G Ahn KJ (2017) A taxonomic study of Korean *Aloconota* Thomson (Coleoptera: Staphylinidae: Aleocharinae) with descriptions of five new species. Journal of Natural History 51: 1–26. 10.1080/00222933.2017.1347298

[B45] Lohse GA (1974) Staphylinidae II (Hypocyphtinae und Aleocharinae). In: Freude H, Harde KW, Lohse GA (Eds) Die Käfer Mitteleuropas, Band 5. Goecke & Evers, Krefeld, 381 pp.

[B46] Lohse GA (1984) *Trichiusaimmigrata* n. sp. eine neue Adventivart aus Mitteleuropa. Entomologische Blatter 80: 163–165.

[B47] Lohse GA Klimaszewski J Smetana A (1990) Revision of Arctic Aleocharinae of North America (Coleoptera: Staphylinidae). The Coleopterists Bulletin 44: 121–202. https://www.jstor.org/stable/4008713

[B48] Lundberg S (2006) Nytillkomna och strukna skalbaggsarter sedan 1995 års Catalogus Coleopterorum Sueciae [New and excluded beetle species since 1995 year’s Catalogus Coleopterorum Sueciae]. Entomologisk Tidskrift 127: 101–111. http://coleoptera.se/CCS1995/ET2006%20101-111.pdf

[B49] Machulka V (1941) Einige neuen Staphyliniden aus Böhmen. I. Sborník Entomologického Oddělení při Zoologických Sbírkách Národního Musea v Praze 19: 98–102.

[B50] Majka CG Klimaszewski J (2008) New records of Canadian Aleocharinae (Coleoptera: Staphylinidae). In: Majka CG Klimaszewski J (Eds) Biodiversity, biosystematics, and ecology of Canadian Coleoptera.ZooKeys 2: 85–114. 10.3897/zookeys.2.7

[B51] Majka CG Sikes DS (2009) Thomas L. Casey and Rhode Island’s precinctive beetles: Taxonomic lessons and the utility of distributional checklists. ZooKeys 22: 267–283. 10.3897/zookeys.22.93

[B52] McClenaghan B Nol E Kerr KCR (2019) DNA metabarcoding reveals the broad and flexible diet of a declining aerial insectivore. The Auk 136: uky003. 10.1093/auk/uky003

[B53] Muona J (1983) Two new Palaearctic *Atheta* species (Coleoptera, Staphylinidae). Annales Entomologici Fennici 49: 57–58.

[B54] Muona J (1984) Review of the Palearctic Aleocharinae also occurring in North America (Coleoptera: Staphylinidae). Entomologica Scandinavica 15: 227–231. 10.1163/187631284X00190

[B55] Newton AF (2019) StaphBase: Staphyliniformia world catalog database (version Nov 2018). In: Roskov Y, Ower G, Orrell T, Nicolson D, Bailly N, Kirk PM, Bourgoin T, DeWalt RE, Decock W, Nieukerken E van, Zarucchi J, Penev L (Eds) Species 2000 & ITIS Catalogue of Life, 2019 Annual Checklist. Species 2000: Naturalis, Leiden. ISSN 2405-884X. www.catalogueoflife.org/annual-checklist/2019

[B56] Palm T (1968) Skalbaggar. Coleoptera, Kortvingar: Fam. Staphylinidae, Underfam. Aleocharinae (Deinopsis-Trichomicra). 5. Svensk Insektfauna 51: 1–113.

[B57] Palm T (1970) Skalbaggar. Coleoptera, Kortvingar: Fam. Staphylinidae, Underfam. Aleocharinae (Atheta). 6. Svensk Insektfauna 52: 117–296.

[B58] Paśnik G (2010) Phylogeny and Generic Classification of Tachyusini (Coleoptera, Staphylinidae: Aleocharinae). Institute of Systematics and Evolution of Animals, Polish Academy of Sciences, Kraków, 129 pp.

[B59] Pentinsaari M Vos R Mutanen M (2017) Algorithmic single-locus species delimitation: effects of sampling effort, variation and nonmonophyly in four methods and 1870 species of beetles. Molecular Ecology Resources 17: 393–404. 10.1111/1755-0998.12557 27292571

[B60] Pentinsaari M Anderson R Borowiec L Bouchard P Brunke AJ Douglas H Smith ABT Hebert PDN (2019) DNA barcodes reveal 63 overlooked species of Canadian beetles (Insecta, Coleoptera). ZooKeys 894: 53–150. 10.3897/zookeys.894.37862 31844409 PMC6906170

[B61] Ratnasingham S Hebert PDN (2013) A DNA-based registry for all animal species: the Barcode Index Number (BIN) system. PLoS ONE 8: e66213. 10.1371/journal.pone.0066213 PMC3704603 23861743

[B62] Rulik B Eberle J von der Mark L Thormann J Jung M Köhler F Apfel W Weigel A Kopetz A Köhler J Fritzlar F Hartmann M Hadulla K Schmidt J Hörren T Krebs D Theves F Eulitz U Skale A Rohwedder D Kleeberg A Astrin JJ Geiger MF Wägele JW Grobe P Ahrens D (2017) Using taxonomic consistency with semi-automated data pre-processing for high quality DNA barcodes. Methods in Ecology and Evolution 8: 1878–1887. 10.1111/2041-210X.12824

[B63] Sawada K (1970) Aleocharinae (Staphylinidae, Coleoptera) of the IBP-Station in the Shiga Heights, Central Japan, II. Contributions from the Biological Laboratory of Kyoto University 23: 33–60. https://repository.kulib.kyoto-u.ac.jp/dspace/bitstream/2433/155945/1/cbl02301_033.pdf

[B64] Schülke M Smetana A (2015) Staphylinidae. In: Löbl I, Löbl D (Eds) Catalogue of Palaearctic Coleoptera (Vol. 2). Hydrophiloidea-Staphylinoidea. Revised and updated edition. Koninklijke Brill, Leiden, 1702 pp. [304–1134.]

[B65] Seevers CH (1978) A generic and tribal revision of the North American Aleocharinae (Coleoptera: Staphylinidae) [with additions and annotations by Lee H. Herman]. Fieldiana Zoology 71: [vi +] 289 pp. https://www.biodiversitylibrary.org/page/2787293

[B66] Sikes DS Bowser M Morton JM Bickford C Meierotto S Hildebrandt K (2017a) Building a DNA barcode library of Alaska’s non-marine arthropods. Genome 60: 248–259. 10.1139/gen-2015-0203 28106469

[B67] Smetana A (1995) Rove beetles of the subtribe Philonthina of America north of Mexico (Coleoptera: Staphylinidae). Classification, phylogeny and taxonomic revision. Memoirs on Entomology, International 3: 1–945.

[B68] Staniec B Pietrykowska-Tudruj E Zagaja M (2010) Description of the larva and pupa of *Haploglossapicipennis* (Gyllenhal, 1827) and larva of *H.nidicola* (Fairmaire, 1852) (Coleoptera: Staphylinidae: Aleocharinae) with taxonomic remarks. Entomologica Fennica 21: 151–167. 10.33338/ef.84526

[B69] Webster R Klimaszewski J Bourdon C Sweeney J Hughes CC Labrecque M (2016) Further contributions to the Aleocharinae (Coleoptera, Staphylinidae) fauna of New Brunswick and Canada including descriptions of 27 new species. ZooKeys 573: 85–216. 10.3897/zookeys.573.7016 PMC4829927 27110168

